# Supplementary material for: Oxidative stress from DGAT1 oncoprotein inhibition in melanoma suppresses tumor growth when ROS defenses are also breached
Source: Cell Rep. 2022 Jun 21;39(12):110995. doi: 10.1016/j.celrep.2022.110995 (PMC9638004; doi:10.1016/j.celrep.2022.110995)
Supplement: Document S1. Figures S1–S6 and Table S8 [file mmc1.pdf]

Cell Reports, Volume *39*

Supplemental information

Oxidative stress from DGAT1 oncoprotein inhibition in melanoma suppresses tumor growth when ROS defenses are also breached

Daniel J. Wilcock, Andrew P. Badrock, Chun W. Wong, Rhys Owen, Melissa Guerin, Andrew D. Southam, Hannah Johnston, Brian A. Telfer, Paul Fullwood, Joanne Watson, Harriet Ferguson, Jennifer Ferguson, Gavin R. Lloyd, Andris Jankevics, Warwick B. Dunn, Claudia Wellbrock, Paul Lorigan, Craig Ceol, Chiara Francavilla, Michael P. Smith, and Adam F.L. Hurlstone

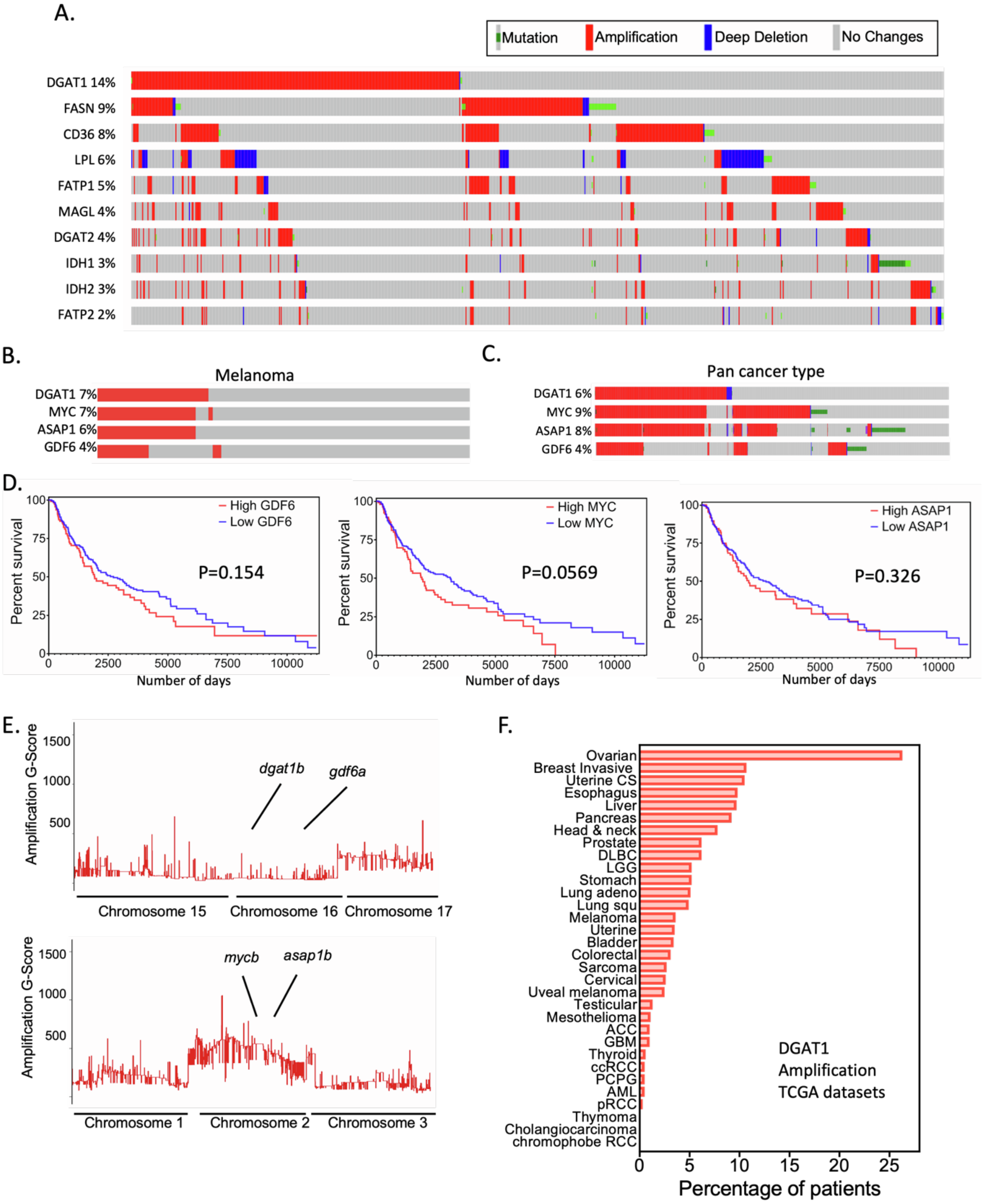
A.
Mutation
Amplification
Deep Deletion
No Changes
DGAT1 14%
FASN 9%
CD36 8%
LPL 6%
FATP1 5%
MAGL 4%
DGAT2 4%
IDH1 3%
IDH2 3%
FATP2 2%
B.
Melanoma
DGAT1 7%
MYC 7%
ASAP1 6%
GDF6 4%
C.
Pan cancer type
DGAT1 6%
MYC 9%
ASAP1 8%
GDF6 4%
D.
High GDF6
Low GDF6
P=0.154
High MYC
Low MYC
P=0.0569
High ASAP1
Low ASAP1
P=0.326
Percent survival
Number of days
E.
Amplification G-Score
dgat1b
gdf6a
Chromosome 15
Chromosome 16
Chromosome 17
mycb
asap1b
Chromosome 1
Chromosome 2
Chromosome 3
F.
Ovarian
Breast Invasive
Uterine CS
Esophagus
Liver
Pancreas
Head & neck
Prostate
DLBC
LGG
Stomach
Lung adeno
Lung squ
Melanoma
Uterine
Bladder
Colorectal
Sarcoma
Cervical
Uveal melanoma
Testicular
Mesothelioma
ACC
GBM
Thyroid
ccRCC
PCPG
AML
pRCC
Thymoma
Cholangiocarcinoma
chromophobe RCC
DGAT1
Amplification
TCGA datasets
Percentage of patients

Figure S1. *DGAT1* amplification and up-regulation associates with poor prognosis in melanoma (related to Figure 1)

(A) Alterations in indicated genes in the TGCA pan-cancer data set accessed via cBioPortal.

(B) Alterations in indicated genes in the TCGA firehose legacy melanoma data set accessed via cBioPortal.

(C) TGCA pan-cancer data set accessed via cBioPortal quantifying alterations in indicated genes.

(D) Kaplan-Meier survival plots for co-amplified genes found on chromosome 8 in melanoma (top 25% *vs* bottom 75%, TCGA database).

(E) G-Score of amplified regions of zebrafish chromosomes found in $BRAF^{V600E}$-positive; *tp53* mutant tumors, locations of human chromosome 8 zebrafish paralogues identified.

(F) TGCA pan-cancer data set accessed via cBioPortal quantifying amplification of *DGAT1* per cancer type.

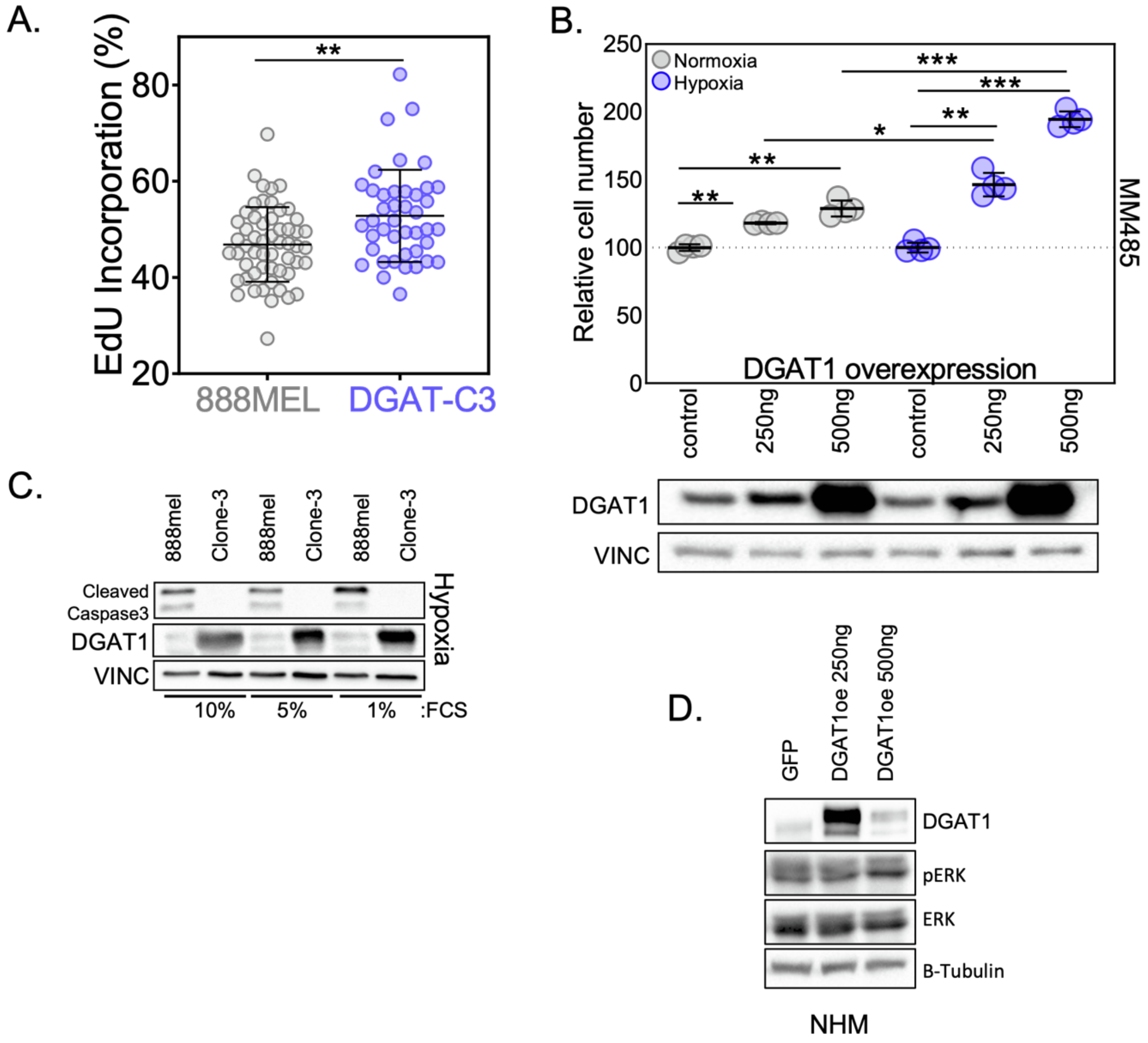

Figure S2. DGAT1 functions as an oncoprotein in zebrafish and human melanocytes (related to Figure 2)

(A) Quantification of the percentage of 888MEL parental cells or Clone 3 DGAT1 over-expressing cells in S-phase following EdU incorporation (Mean ± SD, n>40).

(B) Relative cell number determined by crystal violet following transfection with DGAT1 overexpression vector or an empty vector control under normoxic or hypoxic conditions for 48 h. (1 % O_2) (upper) (Mean, n>3). Corresponding protein expression of DGAT1 (lower).

(C) Protein expression in 888mel parental or Clone 3 DGAT1 overexpressing cells of DGAT1 and cleaved caspase 3 in indicated FCS serum levels under hypoxic (1% O2) conditions.

(D) Protein expression of DGAT1, phospho-ERK and ERK in NHM following overexpression of DGAT1 or GFP.

(A and B) For significance: $*p < 0.05$, $**p < 0.01$, and $***p < 0.001$

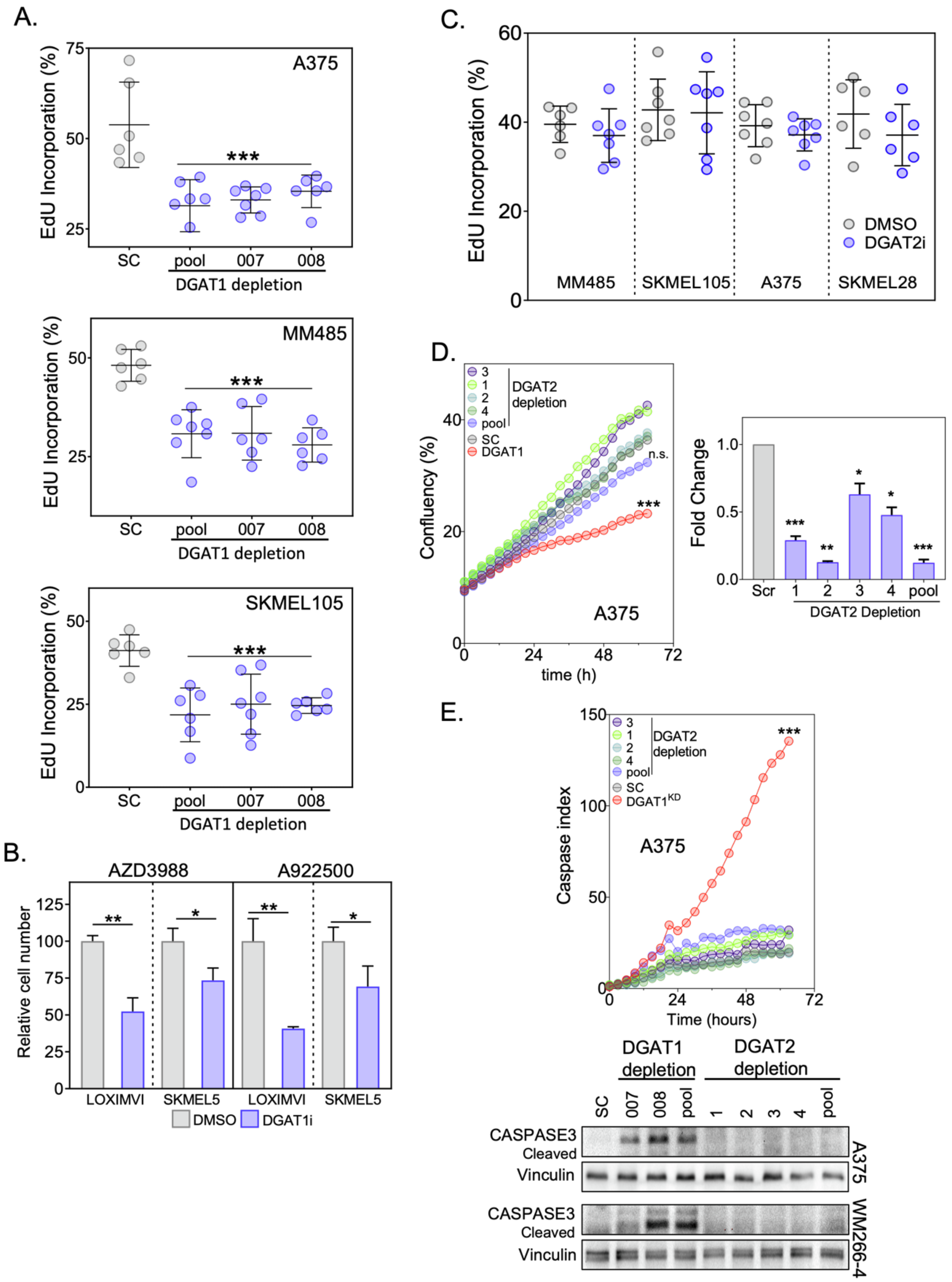

A.
A375
EdU Incorporation (%)
***
SC
pool
007
008
DGAT1 depletion
MM485
SKMEL105
B.
AZD3988
A922500
Relative cell number
LOXIMVI
SKMEL5
DMSO
DGAT1i
C.
DMSO
DGAT2i
MM485
SKMEL105
A375
SKMEL28
D.
DGAT2 depletion
pool
SC
DGAT1
n.s.
Confluency (%)
time (h)
Fold Change
Scr
DGAT2 Depletion
E.
Caspase index
DGAT1KD
Time (hours)
DGAT1 depletion
DGAT2 depletion
CASPASE3
Cleaved
Vinculin
WM266-4

Figure S3. DGAT1 antagonism decreases melanoma cell proliferation and survival (related to Figure 3)

(A) Quantification of the population of cells in S-phase using EdU incorporation following transfection with DGAT1 targeting siRNA (007, 008, pool) or scrambled control for 48 h (Mean ± SD, n>5).

(B) Relative cell number determined by crystal violet staining following 72 h treatment with/without AZD3988 or A922500 (Mean ± SD, n>3).

(C) Quantification of the percentage of cells in S-phase using EdU incorporation following 24 h treatment with/without DGAT2 inhibitor (Mean ± SD, n>5).

(D) Confluency curves determined by time-lapse microscopy in A375 cells following transfection with DGAT2 (001, 002, 003, 004, pool) or DGAT1 targeting scrambled siRNA using an Incucyte zoom system (Mean, n>3) (left). RT-qPCR analysis of DGAT2 gene expression in A375 cells following transfection with DGAT2 targeting siRNA (001, 002, 003, 004, pool) or scrambled control for 48 h (Mean ± SD, n=3) (right).

(E) Cleaved caspase index in A375 cells following transfection with either a DGAT2 targeting (001, 002, 003, 004, pool), DGAT1 targeting or a scrambled siRNA determined by time-lapse microscopy using an Incucyte zoom system (Mean, n=3) (upper). Corresponding protein expression of cleaved caspase3 following transfection with either a DGAT2 targeting siRNA (001, 002, 003, 004, pool), DGAT1 targeting siRNA (007, 008, pool) or a scrambled control (lower).

For significance: $*p < 0.05$, $**p < 0.01$, and $***p < 0.001$

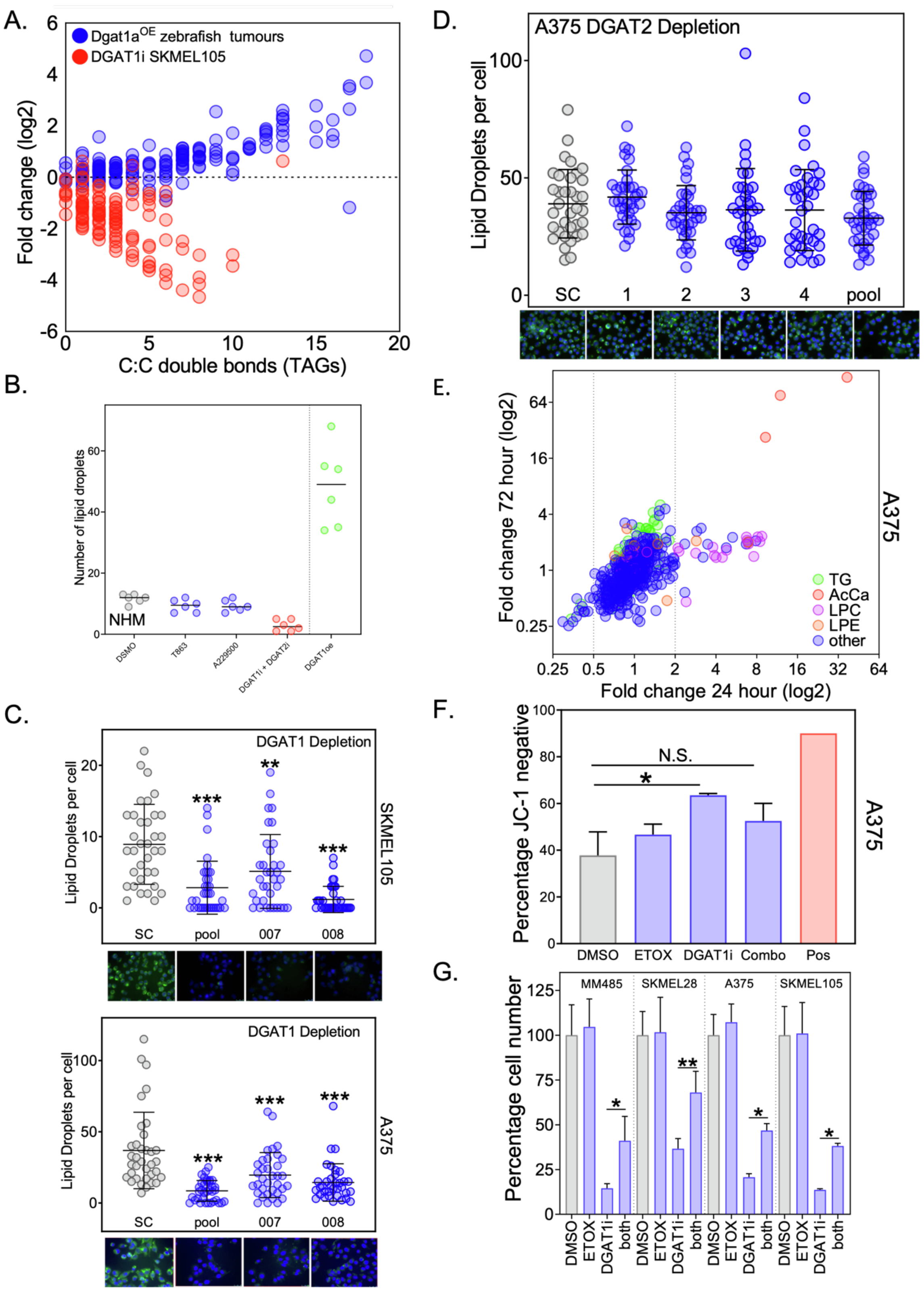
A.
Dgat1a^OE zebrafish tumours
DGAT1i SKMEL105
Fold change (log2)
C:C double bonds (TAGs)
B.
Number of lipid droplets
NHM
DSMO
T863
A229500
DGAT1i + DGAT2i
DGAT1oe
C.
DGAT1 Depletion
Lipid Droplets per cell
SC
pool
007
008
SKMEL105
A375
D.
A375 DGAT2 Depletion
Lipid Droplets per cell
SC
1
2
3
4
pool
E.
Fold change 72 hour (log2)
Fold change 24 hour (log2)
TG
AcCa
LPC
LPE
other
A375
F.
Percentage JC-1 negative
N.S.
DMSO
ETOX
DGAT1i
Combo
Pos
A375
G.
Percentage cell number
MM485
SKMEL28
A375
SKMEL105
DMSO
ETOX
DGAT1i
both

Figure S4. DGAT1-formed lipid droplets act as caretakers of mitochondrial health (related to Figure 4)

(A) UHPLC- lipidomic analysis of NRASG12D-positive Dgat1a-over-expressing (n=6) tumors and SKMEL105 cells following treatment with A922500 for 72 h and showing the number of carbon-carbon double bonds in TAG species compared to the fold increase relative to control samples.

(B) Number of lipid droplets per cell in NHM treated with DGAT1 inhibitors alone or combined with DGAT2 inhibitor or in NHM overexpressing DGAT1 quantified using BODIPY staining (Mean ± SD, n = 6 replicates).

(C) Representative images and quantification of the number of lipid droplets per cell following BODIPY staining. Indicated cell lines were transfected with either DGAT1 targeting siRNA (007, 008, pool) or scrambled control for 48 h (Mean ± SD, n>30).

(D) Representative images and quantification of the number of lipid droplets per cell following BODIPY staining. A375 cells were transfected with either DGAT2 targeting siRNA (001, 002, 003, 004, pool) or a scrambled control for 48 h (Mean ± SD, n>30).

(E) UHPLC- lipidomic analysis of A375 cells following treatment with/without A922500 for 24 or 72 h (showing lipid species that were annotated using MS/MS). Fold-change calculated relative to DMSO treated control (All conditions n=3).

(F) A375 cells were stained with JC-1 dye following treatment with/without A922500 for 48 h and with/without Etomoxir for 4 h. The percentage of cells that lost red J-aggregates was calculated by using 1 μM CCP as a positive control for loss of mitochondrial membrane potential and comparing this to untreated cells to create two populations of cells in the flow cytometry analysis (Mean± SD, n>3).

(G) Relative cell number as determined by crystal violet staining, indicted cell 24 lines were treated with/without A922500 for 48 h and with/without Etomoxir for 24 h (Mean± SD, n=3).

(C, F and G) For significance: $*p < 0.05$, $**p < 0.01$, and $***p < 0.001$

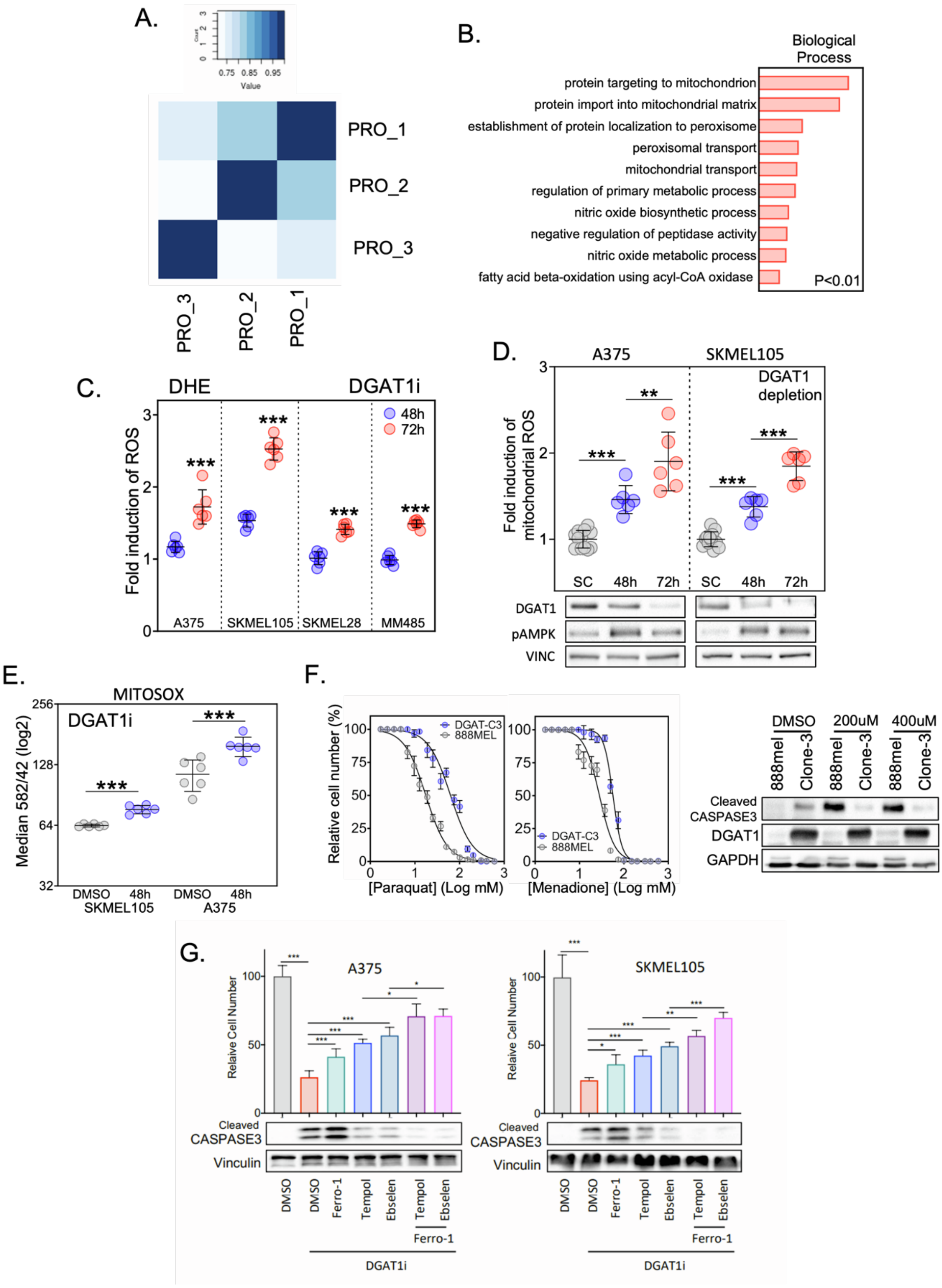

A.
B.
Biological Process
protein targeting to mitochondrion
protein import into mitochondrial matrix
establishment of protein localization to peroxisome
peroxisomal transport
mitochondrial transport
regulation of primary metabolic process
nitric oxide biosynthetic process
negative regulation of peptidase activity
nitric oxide metabolic process
fatty acid beta-oxidation using acyl-CoA oxidase
P<0.01
PRO_1
PRO_2
PRO_3
C.
DHE
DGAT1i
Fold induction of ROS
48h
72h
A375
SKMEL105
SKMEL28
MM485
D.
A375
SKMEL105
DGAT1 depletion
Fold induction of mitochondrial ROS
SC
48h
72h
DGAT1
pAMPK
VINC
E.
MITOSOX
DGAT1i
Median 582/42 (log2)
DMSO
48h
SKMEL105
A375
F.
Relative cell number (%)
DGAT-C3
888MEL
[Paraquat] (Log mM)
[Menadione] (Log mM)
DMSO
200uM
400uM
888mel
Clone-3
Cleaved CASPASE3
DGAT1
GAPDH
G.
A375
SKMEL105
Relaive Cell Number
Cleaved CASPASE3
Vinculin
DMSO
Ferro-1
Tempol
Ebselen
DGAT1i

Figure S5. DGAT1 promotes survival of melanoma cells through suppressing ROS generation (related to Figure 5)

(A) Pearson correlation between total proteome samples following treatment of A375 cells with/without A922500 for 72 h (n=3).

(B) Gene Ontology analysis of up-regulated genes (114) in A375 cells following treatment with A922500 for 72 h compared to DMSO treated control. Gene Ontology analysis was carried out using biological processes (enrichR) ranked by combined score.

(C) ROS levels quantified using dihydroethidium (DHE) fluorescence following treatment with/without A922500 for 48-72 h. Fold change calculated relative to DMSO treated control (Mean, n>4).

(D) ROS levels quantified using mitosox fluorescence following transfection with either DGAT1 targeting siRNA or a scrambled control for 48-72 h. Fold-change calculated relative to scrambled control (Mean ± SD, n=3) (upper). Corresponding protein expression of DGAT1 and phospho-AMPK (lower).

(E) ROS levels quantified using mitosox fluorescence following treatment with A922500 for 48 h (Mean ± SD, n=6).

(F) Drug-dose response curve after 72 h treatment with ROS inducers (left) (Mean ± SD, n=3). Protein expression of cleaved-caspase3 and DGAT1 following 72 h paraquat treatment (right).

(G) Relative cell number following crystal violet staining, indicated cells were either with/without A922500, Ferrsostatin-1 (Ferro-1), Ebselen and Tempol (Mean ± SD, n=3) (Upper). Corresponding protein expression of cleaved caspase-3 in indicated cell lines.

For significance: $*p < 0.05$, $**p < 0.01$, and $***p < 0.001$

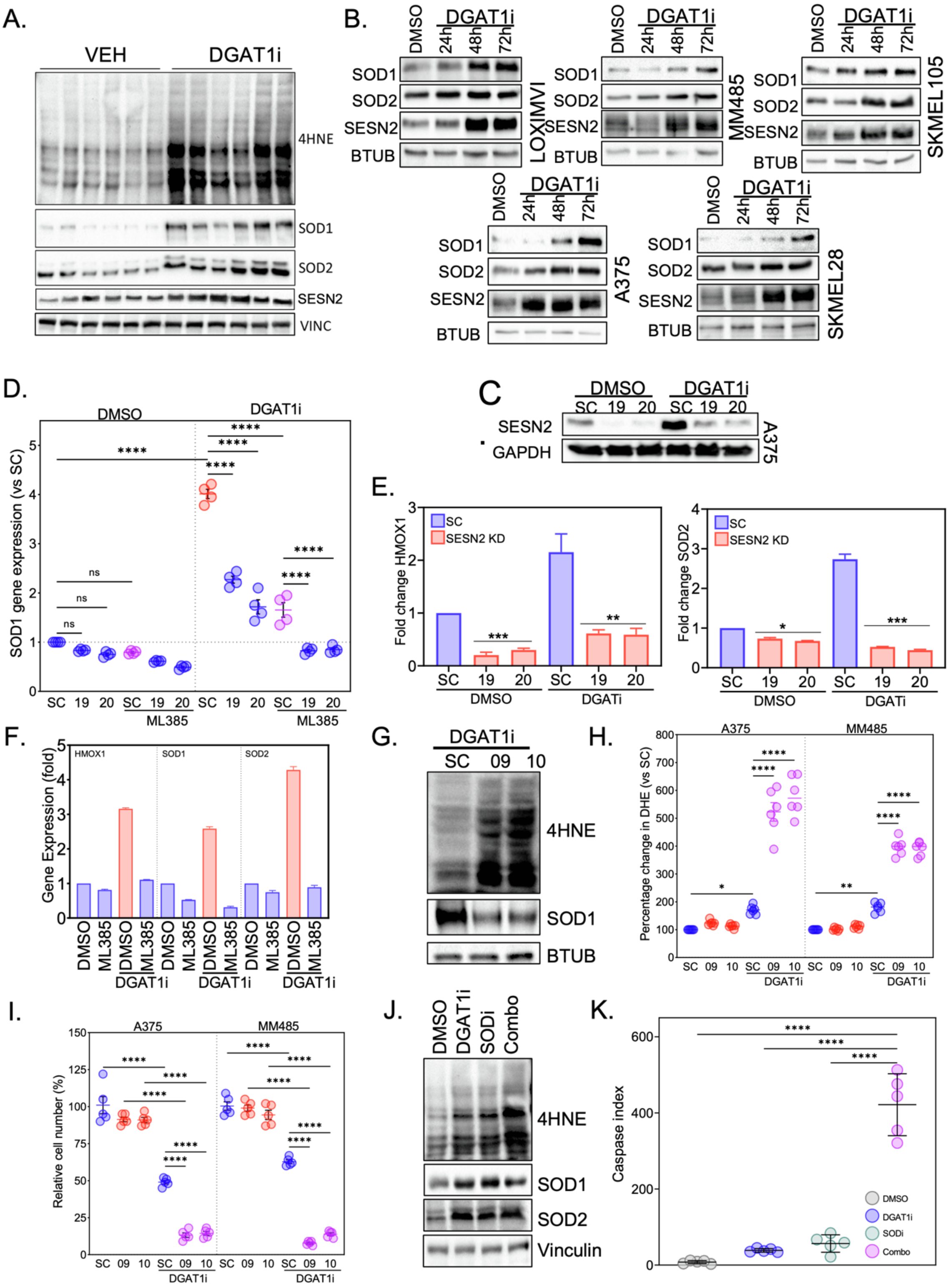

A.
VEH
DGAT1i
4HNE
SOD1
SOD2
SESN2
VINC
B.
DMSO
DGAT1i
24h
48h
72h
SOD1
SOD2
SESN2
BTUB
LOXIMVI
MM485
SKMEL105
A375
SKMEL28
C
DMSO
DGAT1i
SC 19 20 SC 19 20
SESN2
GAPDH
A375
D.
DMSO
DGAT1i
SOD1 gene expression (vs SC)
ns
****
SC 19 20 SC 19 20 SC 19 20 SC 19 20
ML385
ML385
E.
Fold change HMOX1
Fold change SOD2
SC
SESN2 KD
***
**
*
DMSO
DGATi
F.
Gene Expression (fold)
HMOX1
SOD1
SOD2
DMSO
ML385
DGAT1i
G.
DGAT1i
SC 09 10
4HNE
SOD1
BTUB
H.
A375
MM485
Percentage change in DHE (vs SC)
I.
Relative cell number (%)
J.
DMSO
DGAT1i
SODi
Combo
4HNE
SOD1
SOD2
Vinculin
K.
Caspase index
DMSO
DGAT1i
SODi
Combo

Figure S6 Combined DGAT1 and SOD1 inhibition halts tumor growth (related to Figure 6)

(A) 4-Hydroxynonenal (4HNE) protein conjugate abundance and protein expression of SOD1, SOD2 and SESN2 in A375 tumors treated with either vehicle or A922500 (DGAT1i).

(B) Protein expression of SOD1, SOD2 and SESN2 following A922500 treatment for 24-72 hours in a panel of melanoma cell lines.

(C) Protein expression of SESN2 in A375 cells following treatment with/without A922500 and with either scrambled control (SC) or SESN2 targeting siRNA (19 or 20) for 48 hours.

(D) SOD1 gene expression in A375 cells following treatment with/without A922500 and ML385 and treatment with either scrambled control (SC) or SESN2 targeting siRNA (19 or 20) for 48 hours. (Mean ± SD, n=3)

(E) HMOX (left) & SOD2 (right) gene expression in A375 cells following treatment with/without A922500 and with either scrambled control (SC) or SESN2 targeting siRNA (19 or 20) for 48 hours. (Mean ± SD, n=3)

(F) HMOX, SOD1 & SOD2 gene expression in A375 cells following treatment with/without A922500 and with/without ML385 for 48 hours. (Mean ± SD, n=3)

(G) 4HNE protein conjugate abundance and protein expression of SESN2 in A375 cells following treatment with A922500 and with either scrambled control (SC) or SOD1 targeting siRNA (09 or 10) for 48 hours.

(H) Quantification of ROS levels using dihydroethidium (DHE) fluorescence in A375 cells following treatment with/without A922500 and with either scrambled control (SC) or SOD1 targeting siRNA (09 or 10) for 48 hours. (Mean ± SD, n=6)

(I) Relative cell number following crystal violet staining in A375 cells following treatment with/without A922500 and with either scrambled control (SC) or SOD1 targeting siRNA (09 or 10) for 48 hours. (Mean ± SD, n=5)

(J) 4HNE protein conjugate abundance and protein expression of SOD1 and SOD2 in A375 cells following treatment with either A922500 or ATN-224 (SODi) alone or in combination.

(K) Cleaved-caspase index in A375 cells following treatment with either A922500 or ATN-224 alone or in combination (combo). Fold-change relative to DMSO (Mean ± SD, n=5). For significance: $*p < 0.05$, $**p < 0.01$, and $***p < 0.001$

Cell line	Genetic drivers	DGAT1 status
888MEL	$BRAF^{V600E}$	DGAT1 low
SKMEL28	$BRAF^{V600E}$	DGAT1 medium
SKMEL2	$NRAS^{Q61R}$	DGAT1 over expressed
MM485	$NRAS^{Q61R}$	DGAT1 over expressed
A375	$BRAF^{V600E}$	DGAT1 over expressed
SKMEL105	$BRAF^{V600E}$	DGAT1 over expressed
LOXIMVI	$BRAF^{V600E}$	DGAT1 amplified
SKMEL5	$BRAF^{V600E}$	DGAT1 amplified

Supplementary Table 8 DGAT1 status of cell lines used in this study (related to STAR Methods)
