# Supplementary material for: Oxidative stress from DGAT1 oncoprotein inhibition in melanoma suppresses tumor growth when ROS defenses are also breached
Source: Cell Rep. 2022 Jun 21;39(12):110995. doi: 10.1016/j.celrep.2022.110995 (PMC9638004; doi:10.1016/j.celrep.2022.110995)
Supplement: Document S2. Article plus supplemental information [file mmc9.pdf]

Cell Reports

Article

Oxidative stress from DGAT1 oncoprotein inhibition in melanoma suppresses tumor growth when ROS defenses are also breached

Graphical abstract

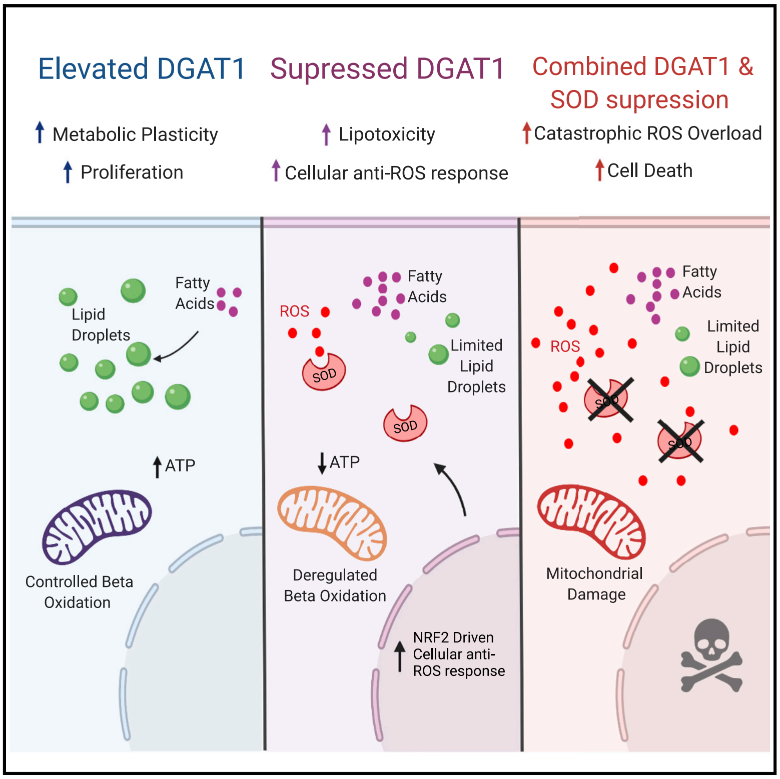

Highlights

- DGAT1 is up-regulated in melanoma cells and is an oncoprotein
- DGAT1 induced lipid droplet formation creates a reservoir for excess fatty acid
- DGAT1 inhibition causes excess fatty acid oxidation, ROS generation, and NRF2 activation
- Suppression of tumor growth requires inhibiting both DGAT1 and the NRF2 target SOD1

Authors

Daniel J. Wilcock, Andrew P. Badrock, Chun W. Wong, ..., Chiara Francavilla, Michael P. Smith, Adam F.L. Hurlstone

Correspondence

michael.smith-8@manchester.ac.uk (M.P.S.), adam.hurlstone@manchester.ac.uk (A.F.L.H.)

In brief

Wilcock et al. find that DGAT1, which stimulates triglyceride and lipid droplet formation, is a melanoma oncoprotein. Lipid droplets are a fatty acid reservoir, controlling fatty acid oxidation. With DGAT1 inhibited, deregulated fatty acid oxidation results in ROS accumulation that proves lethal when the NRF2 target SOD1 is also inhibited.

Wilcock et al., 2022, Cell Reports *39*, 110995
June 21, 2022 © 2022 The Authors.
https://doi.org/10.1016/j.celrep.2022.110995

CellPress

Cell Reports

CellPress
OPEN ACCESS

Article

Oxidative stress from DGAT1 oncoprotein inhibition in melanoma suppresses tumor growth when ROS defenses are also breached

Daniel J. Wilcock,[1,10] Andrew P. Badrock,[2,10,11] Chun W. Wong,[3] Rhys Owen,[1] Melissa Guerin,[4] Andrew D. Southam,[5,6] Hannah Johnston,[1] Brian A. Telfer,[1] Paul Fullwood,[2] Joanne Watson,[2] Harriet Ferguson,[2] Jennifer Ferguson,[2] Gavin R. Lloyd,[5,6] Andris Jankevics,[5,6] Warwick B. Dunn,[5,6,7] Claudia Wellbrock,[1] Paul Lorigan,[1,8] Craig Ceol,[4] Chiara Francavilla,[2,11] Michael P. Smith,[2,*] and Adam F.L. Hurlstone[3,9,12,*]

[1]Division of Cancer Studies, School of Medical Sciences, Faculty of Biology, Medicine and Health, The University of Manchester, Manchester M13 9PT, UK

[2]Division of Molecular and Cellular Function, School of Biological Sciences, Faculty of Biology, Medicine and Health, The University of Manchester, Dover Street, Manchester M13 9PT, UK

[3]Division of Infection Immunity and Respiratory Medicine, School of Biological Sciences, Faculty of Biology, Medicine and Health, The University of Manchester, Dover Street, Manchester M13 9PT, UK

[4]Program in Molecular Medicine, Department of Molecular, Cell and Cancer Biology, University of Massachusetts Medical School, Worcester, MA 01605, USA

[5]School of Biosciences, Edgbaston, University of Birmingham, Birmingham B15 2TT, UK

[6]Phenome Centre Birmingham, University of Birmingham, Edgbaston, Birmingham B15 2TT, UK

[7]Institute of Metabolism and Systems Research, University of Birmingham, Edgbaston, Birmingham B15 2TT, UK

[8]Department of Medical Oncology, The Christie NHS Foundation Trust, Wilmslow Road, Withington, Manchester M20 4BX, UK

[9]Lydia Becker Institute of Immunology, The University of Manchester, Dover Street, Manchester M13 9PT, UK

[10]These authors contributed equally

[11]Senior author

[12]Lead contact

*Correspondence: michael.smith-8@manchester.ac.uk (M.P.S.), adam.hurlstone@manchester.ac.uk (A.F.L.H.)

https://doi.org/10.1016/j.celrep.2022.110995

SUMMARY

Dysregulated cellular metabolism is a cancer hallmark for which few druggable oncoprotein targets have been identified. Increased fatty acid (FA) acquisition allows cancer cells to meet their heightened membrane biogenesis, bioenergy, and signaling needs. Excess FAs are toxic to non-transformed cells but surprisingly not to cancer cells. Molecules underlying this cancer adaptation may provide alternative drug targets. Here, we demonstrate that diacylglycerol *O*-acyltransferase 1 (DGAT1), an enzyme integral to triacylglyceride synthesis and lipid droplet formation, is frequently up-regulated in melanoma, allowing melanoma cells to tolerate excess FA. DGAT1 over-expression alone transforms p53-mutant zebrafish melanocytes and co-operates with oncogenic BRAF or NRAS for more rapid melanoma formation. Antagonism of DGAT1 induces oxidative stress in melanoma cells, which adapt by up-regulating cellular reactive oxygen species defenses. We show that inhibiting both DGAT1 and superoxide dismutase 1 profoundly suppress tumor growth through eliciting intolerable oxidative stress.

INTRODUCTION

The growth and survival of cancer cells is underpinned by dysregulated cellular metabolism (DeBerardinis and Chandel, 2016), which further promotes cancer progression by reprogramming stromal cells, mediating evasion of immune responses, and promoting metastasis (Andrejeva and Rathmell, 2017; Lee et al., 2018; Lyssiotis and Kimmelman, 2017). A central facet of cancer cell metabolism is a shift in glucose usage away from oxidative phosphorylation toward biosynthetic reactions. Without compensation by fatty acid oxidation (FAO) and glutaminolysis, this shift would result in deficiencies in citric acid cycle intermediates needed for ATP production (DeBerardinis and Chandel, 2016).

Although catabolism of fatty acids (FA) maintains ATP production, increased lipogenesis (lipid generation) is simultaneously required for cell membrane synthesis, for which FA are principal building blocks (Koundouros and Poulogiannis, 2020). Furthermore, lipid signaling molecules such as phosphatidylinositides, diacylglycerides (DAG), lysophosphatidic acid, and prostaglandins, implicated in multiple cancer hallmarks (Chen and Huang, 2019), are also derived from FA. To satisfy these competing demands, cancer cells increase FA supply. This can be achieved by initiating *de novo* FA synthesis, normally a function of adipocytes

This is an open access article under the CC BY license (http://creativecommons.org/licenses/by/4.0/).

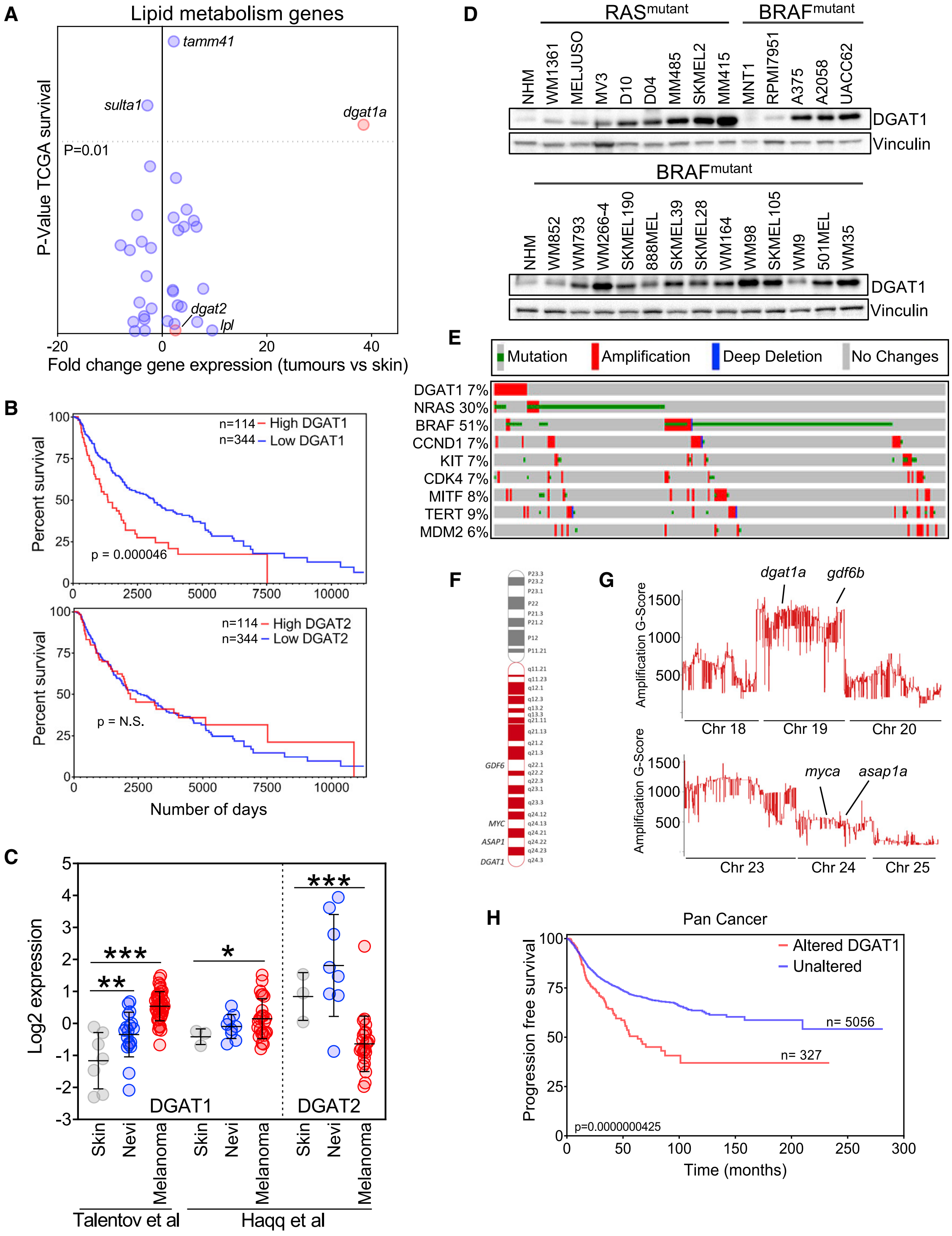

Figure 1. DGAT1 amplification and up-regulation is associated with poor prognosis in melanoma

(A) Patient survival from TCGA melanoma cohort (25% top versus 75% bottom by mRNA abundance, y axis) versus fold change in mRNA expression of lipid metabolism genes in zebrafish tumors (x axis).

(B) Kaplan-Meier survival plot comparing melanoma patients based on expression of *DGAT1* or *DGAT2* (top 25% versus bottom 75%, TCGA dataset).

(C) *DGAT1* and *DGAT2* relative gene expression in skin, nevi, and melanoma tumors from indicated studies. Mean ± SD, n > 3. *p < 0.05, **p < 0.01, and ***p < 0.001.

(legend continued on next page)

and hepatocytes but an acquired characteristic of cancer cells driven by elevated FA synthase (FASN) expression (Koundouros and Poulogiannis, 2020). Alternatively, FA can be acquired from the diet, directly from the circulation or indirectly from adipose tissue. Cancer cells utilize secreted lipases like lipoprotein lipase (LPL) to hydrolyze FA from triglycerides, and FA transporter proteins (FATP) like CD36 and SLC27 family members and FA-binding proteins (FABP) to facilitate uptake (Lengyel et al., 2018). Enhanced FA uptake by cancer cells promotes tumor growth and dissemination (Nieman et al., 2011; Pascual et al., 2017; Watt et al., 2019). Intracellular lipases also liberate FA from intracellular lipid stores (Maan et al., 2018). One such lipase, monoacylglycerol lipase (MAGL or MGLL), is upregulated in aggressive cancers, wherein its suppression impedes tumor growth and metastasis (Nomura et al., 2010).

Whereas adipocytes are adapted to store FA, non-adipose, non-transformed cells that become overloaded with FA suffer from lipotoxicity, characterized by reduced insulin signaling (insulin resistance) and increased cell death (Brookheart et al., 2009). Multiple processes underlie lipotoxicity, including increased ceramide synthesis, dysregulation of phospholipid production compromising mitochondrial and ER membrane integrity, impaired ATP generation, and induction of reactive oxygen species (ROS) (Brookheart et al., 2009). How dysregulated metabolism favoring FA acquisition is tolerated by cancer cells and how the toxic by-products of rampant FAO (principally ROS, including lipid peroxides) are suppressed or neutralized, are incompletely understood. Targeting the ability of cancer cells to manage potentially cytotoxic metabolites that arise from the rewiring of metabolic pathways is an intriguing therapeutic avenue warranting further exploration.

Diacylglycerol *O*-acyltransferase 1 (DGAT1) is an ER-resident enzyme that catalyzes the final step in triacylglyceride (TAG) synthesis from DAG and FA. DGAT1 is required for lipid droplets (LD), cytosolic organelles comprising a core of neutral lipids (mainly triglycerides and sterol esters) delimited by a monolayer of phospholipids (Wilfling et al., 2013). The signficance of DGAT1 up-regulation and subsequent formation of LD to cancer has recently been demonstrated in glioblastoma models, in which DGAT1 inhibition led to tumor cell death through induction of lipotoxicity and oxidative stress (Cheng et al., 2020). Here, we demonstrate the oncogenic ability of DGAT1 in melanoma using zebrafish and find that DGAT1 again exerts this effect primarily through shielding melanoma cells from lipotoxicity. Consequently, DGAT1 inhibition results in oxidative stress in melanoma cells but this is countered by adaptive up-regulation of ROS-neutralizing enzymes such as superoxide dismutase 1 (SOD1). However, simultaneous DGAT1 and SOD1 inhibition leads to catastrophic levels of ROS accumulating in tumor cells, triggering their death.

RESULTS

DGAT1 amplification and up-regulation associates with poor prognosis in melanoma

Few oncoproteins directly dysregulating cellular metabolism have been identified. Point mutations in the isocitrate dehydrogenases IDH1 and IDH2 have been confirmed as oncogenic drivers in only 0.9%–3% of all cancers (Molenaar et al., 2018) (Figure S1A). The gene encoding FASN is frequently amplified in cancer (Koundouros and Poulogiannis, 2020) (Figure S1A); however, FASN inhibitors have failed to gain clinical approval (Flavin et al., 2010). LPL, CD36, FATP1 (SLC27A1), and FATP2 (SLC27A2), implicated in increased FA uptake into melanoma cells (Alicea et al., 2020; Henderson et al., 2019; Pascual et al., 2017; Zhang et al., 2018), are unaffected by point mutation in cancer and display gene amplification in 2%–8% of cases (Figure S1A).

Given this dearth of therapeutic targets but also the significance of lipid metabolism for melanoma development (Fischer et al., 2018), we interrogated genes that we previously identified as accompanying progression of oncogenic RAS-driven melanoma in zebrafish (Henderson et al., 2019). Plotting expression fold change against association with patient survival, *dgat1a*/*DGAT1* emerged as an outlier, being both highly up-regulated in zebrafish melanoma and significantly associated with reduced patient survival (Figures 1A and 1B). In contrast, expression of the gene encoding the functionally related, although structurally distinct, *Dgat2*/DGAT2 did not change significantly, nor was it associated with patient survival (Figures 1A and 1B). Furthermore, we confirmed elevated expression of human *DGAT1* but not *DGAT2* mRNA in melanoma tumors relative to both skin and nevi (Figure 1C) and elevated DGAT1 protein in human melanoma cell lines relative to primary melanocytes irrespective of NRAS or BRAF mutational status (Figure 1D).

We next considered what might underlie DGAT1 up-regulation in melanoma. Visualization of structural alterations of the *DGAT1* gene in the Cancer Genome Atlas firehose legacy cutaneous melanoma dataset using cBioPortal revealed significant focal amplification (as defined by the stringent GISTIC 2.0 algorithm) in up to 7% of melanoma cases with available copy number variation (CNV) data (Figure 1E), comparable to other recognized melanoma oncogenes (namely *CCND1*, *KIT*, *CDK4*, *MITF*, *TERT*, and *MDM2*). An extra copy of chromosome 8q, containing the *DGAT1* locus but also other putative melanoma oncogenes *ASAP1*/*DDEF*, *MYC*, and *GDF6* (Ehlers et al., 2005; Schlagbauer-Wadl et al., 1999; Venkatesan et al., 2018) (Figure 1F), has been observed in approximately 30% of melanoma cases (Bastian et al., 1998). Consistently, *DGAT1*, *ASAP1*, *MYC*, and *GDF6* are co-amplified in melanoma and other cancers (Figures S1B and S1C). However, of these four, *DGAT1* mRNA expression displayed the strongest association with reduced patient survival

(D) Protein expression of DGAT1 and vinculin (loading control). NHM, normal human melanocytes.

(E) Genetic alterations in the TCGA firehose legacy melanoma dataset (counting only samples with CNV data) obtained from cBioPortal.

(F) Schematic depicting human chromosome 8, the amplified arm (red), and known/putative melanoma oncogenes within this region.

(G) G-score of amplified regions of zebrafish chromosomes found in *BRAF*^{V600E}-positive; *tp53* mutant tumors indicating the position of presumed melanoma oncogene homologs.

(H) Kaplan-Meier progression free-survival plot comparing patients across multiple cancer types based on *DGAT1* amplification.

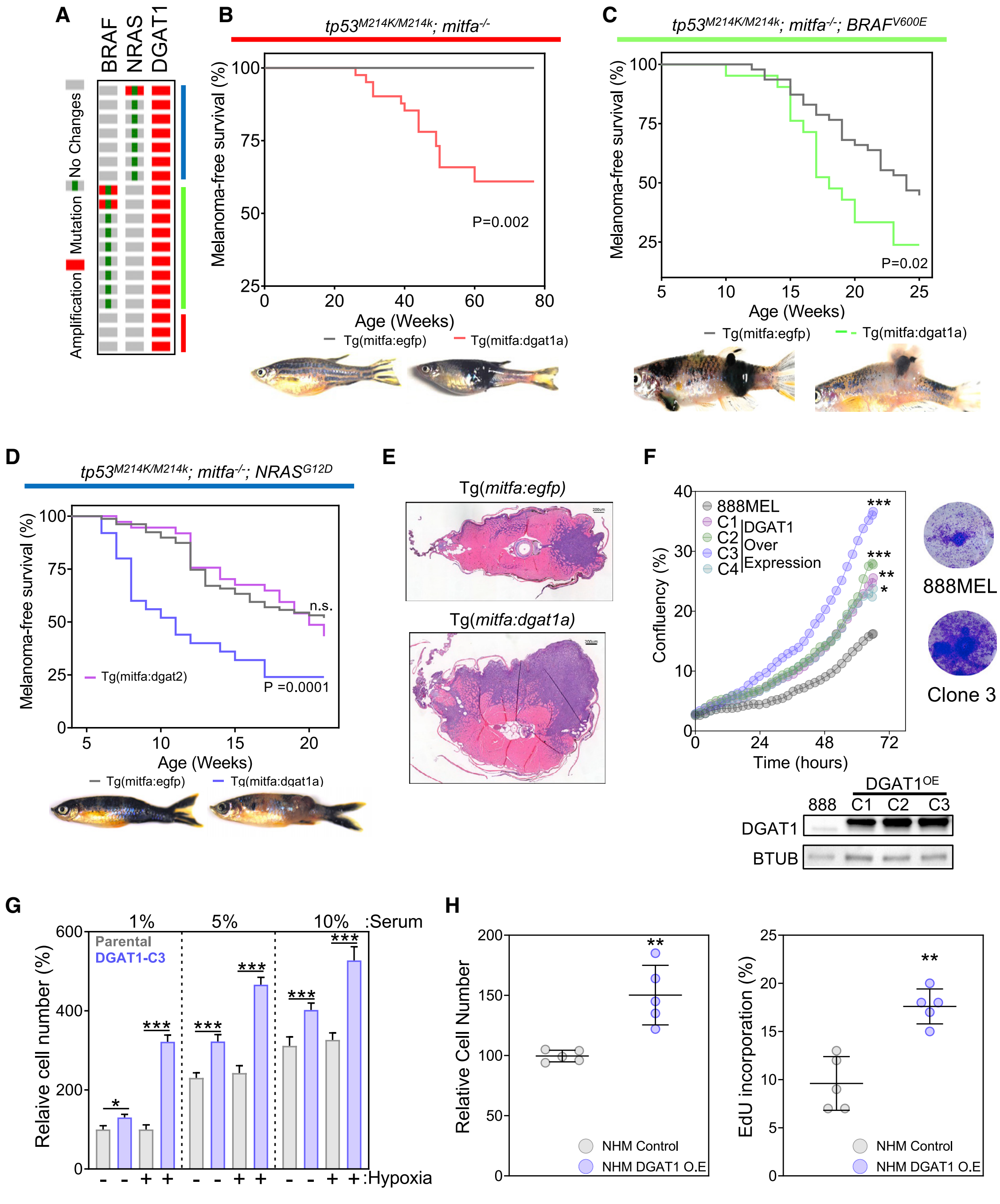

Figure 2. DGAT1 functions as an oncoprotein in zebrafish and human melanocytes

(A) *DGAT1* amplification distribution in TCGA melanoma samples.

(B) Kaplan-Meier plot of melanoma tumor nodule incidence in EGFP control or Dgat1a over-expressing animals on the *tp53* mutant;*nacre* genetic background. Representative images shown for EGFP- and Dgat1a-positive animals at 54 and 76 weeks post-fertilization, respectively (mitfa:egfp n = 20; mitfa:dgat1a n = 20).

(C) As for (B) but on the transgenic *mitfa:BRAF*^{V600E};*tp53* mutant;*nacre* genetic background. Representative images are shown at 12 weeks post-fertilization (mitfa:egfp n = 46; mitfa:dgat1a n = 21).

(D) As for (C) but on the transgenic *mitfa:NRAS*^{G12D};*tp53* mutant;*nacre* genetic background, also shown the effect of Dgat2 over-expression (mitfa:egfp n = 120; mitfa:dgat1a n = 69; mitfa:dgat2 n = 35).

(E) Hematoxylin and eosin-stained transverse sections of EGFP-expressing or Dgat1a-over-expressing melanoma on the transgenic *mitfa:NRAS*^{G12D};*tp53* mutant;*nacre* genetic background. Scale bars, 200 μm

(F) Confluence of parental 888MEL cells and cells following lentiviral transduction with a DGAT1 over-expression vector and clonal selection (mean, n = 3, top left). Corresponding crystal violet staining after 72-h growth (top right). Corresponding protein expression of DGAT1 (bottom).

(legend continued on next page)

CellPress
OPEN ACCESS

(Figures 1B and S1D). Furthermore, our previous comparative oncogenomic analysis (Venkatesan et al., 2018) uncovered amplification of *dgat1a* together with *gdf6b* (both on chromosome 19) in oncogenic-BRAF-driven zebrafish melanoma, concomitant with up-regulation of *dgat1a* and *gdf6b* mRNA (Figure 1G and Table S1). In contrast, neither *dgat1b* nor *gdf6a* on chromosome 16, nor any *myc* or *asap1* paralogs (on chromosomes 2 and 24) were amplified or up-regulated (Figures 1G and S1E, and Table S1). Finally, we found *DGAT1* to be frequently amplified in other human cancers, a feature of many well-characterized oncogenes, most notably in up to 26% of cases of ovarian cancer (Figures S1A and S1F). Strikingly, *DGAT1* amplification was associated with significantly poorer progression-free survival across multiple cancer types (Figure 1H). Thus, from a cancer genomics perspective, *DGAT1* exhibits the hallmarks of an oncogene.

DGAT1 functions as an oncoprotein in zebrafish and human melanocytes

In human melanoma, *DGAT1* amplification was observed to co-occur with *BRAF* and *NRAS* mutation but also independently of either (Figure 2A). To elucidate further the oncogenic potential of *DGAT1*, we utilized a melanocyte rescue and lineage-restricted expression system as described previously (Ceol et al., 2011; Venkatesan et al., 2018). Remarkably, we found that forcing Dgat1a expression in zebrafish melanocytes lacking functional p53 was sufficient to induce melanoma (Figure 2B). Moreover, Dgat1a cooperated with both oncogenic BRAF and NRAS to accelerate the development of nodular tumors (Figures 2C–2E). Thus, in zebrafish, Dgat1 behaves as a melanoma oncoprotein. Significantly, effects of forcing Dgat2 expression were indistinguishable from the non-oncogenic EGFP control (Figure 2D), indicating that Dgat1 stimulation of tumorigenesis cannot be replicated by the functionally related Dgat2.

In human melanoma models, *DGAT1* also exhibited oncogenic characteristics. Forced expression of *DGAT1* in the $DGAT1^{Low}$ melanoma cell line 888MEL led to an increase in cellular proliferation in all four clones tested (Figures 2F and S2A). Cancer cells can sustain their growth despite transient or limited nutrient availability in the tumor microenvironment (Ackerman and Simon, 2014). We therefore investigated the role of DGAT1 over-expression in allowing melanoma cells to tolerate nutrient and oxygen deprivation in culture. DGAT1-overexpressing melanoma cells exhibited a 100%–200% increase in cell number compared with parental $DGAT1^{Low}$ cells under conditions of greatest stress: 1% serum and hypoxia (1% O_2) (Figures 2G, S2B, and S2C). A growth advantage conferred by DGAT1 over-expression was also observed in normal human melanocytes (NHM) (Figures 2H and S2D). Our zebrafish and human models demonstrate that in melanocytes DGAT1 has the hallmarks of an oncoprotein, conferring a growth advantage especially under stress conditions likely encountered in the tumor microenvironment.

DGAT1 antagonism decreases melanoma cell proliferation and survival

Having uncovered DGAT1 as an oncoprotein, we next assessed the impact of DGAT1 antagonism in human melanoma cell lines. DGAT1 depletion using siRNA reduced cell proliferation and decreased the fractions of cells in S-phase (Figures 3A and S3A), in contrast to the increased proliferation and cell cycle progression observed with stable DGAT1 over-expression (Figures 2F and S2A). As DGAT1 has been mooted as a clinical target for combating obesity, several potent and selective small-molecule inhibitors are already available for repurposing (DeVita and Pinto, 2013). To determine whether DGAT1 depletion effects were due to the enzymatic function of DGAT1, we utilized four selective DGAT1 inhibitors (AZD3988, AZD7687, A922500, or T863). Similarly to siRNA-mediated depletion, pharmacological antagonism of DGAT1 suppressed growth of $DGAT1^{High}$ (A375, MM485, and SKMEL105) and $DGAT1^{Amplified}$ (LOXIMVI and SKMEL5) melanoma cells over 96 h (Figures 3B and S3B), accompanied by decreased cell cycle progression (Figure 3B). In contrast, no changes in cell growth or cell cycle progression were observed following DGAT2 depletion or inhibition (Figures S3C–S3E).

Given that DGAT1 over-expression exhibited the largest relative effect on cell growth under conditions of cellular stress, we hypothesized that, conversely, the impact of DGAT1 inhibition would be exacerbated when cells were exposed to nutrient and hypoxic stress. Indeed, we found that DGAT1 antagonism significantly impaired proliferation when external lipid sources were restricted, with the greatest reduction in cell number observed at the lowest levels of serum (Figure 3C). Moreover, exposing cells to hypoxic conditions (1% O_2) led to a further decrease in cell number upon DGAT1 inhibition (Figure 3D). Further highlighting the key role of DGAT1 up-regulation in melanoma cells, even under standard tissue culture conditions, siRNA knockdown or pharmacological inhibition of DGAT1 led to apoptosis, quantified through an increase in cleaved caspase-3 (Figures 3E and 3F). The significant impact of DGAT1 antagonism on melanoma cell proliferation and survival, exacerbated under conditions mimicking tumor microenvironmental stresses, highlights DGAT1 as a possible therapeutic target in melanoma.

DGAT1 is essential for LD formation and acts as a caretaker of mitochondrial health

To address the effect of DGAT1 on melanoma lipid metabolism, we performed ultra-high-performance liquid chromatography-mass spectrometry (UHPLC-MS) to identify and contrast lipid species extracted from $NRAS^{G12D}$ Dgat1a-over-expressing or

(G) Relative cell numberof parental 888MEL cells and cells following lentiviral transduction with a DGAT1 over-expression vector, determined using crystal violet staining following 48-h culture in indicated serum levels under hypoxic (1% O_2) or normoxic conditions (mean ± SEM, n > 3).
(H) Relative cell number of NHM and NHM following lentiviral transduction with a DGAT1 over-expression vector determined using crystal violet staining (left). Percentage of NHM and NHM following lentiviral transduction with a DGAT1 over-expression vector in S-phase by using EdU incorporation (right) (mean ± SEM, n = 5). (F–H) For significance: $^{*}p < 0.05$, $^{**}p < 0.01$, and $^{***}p < 0.001$.

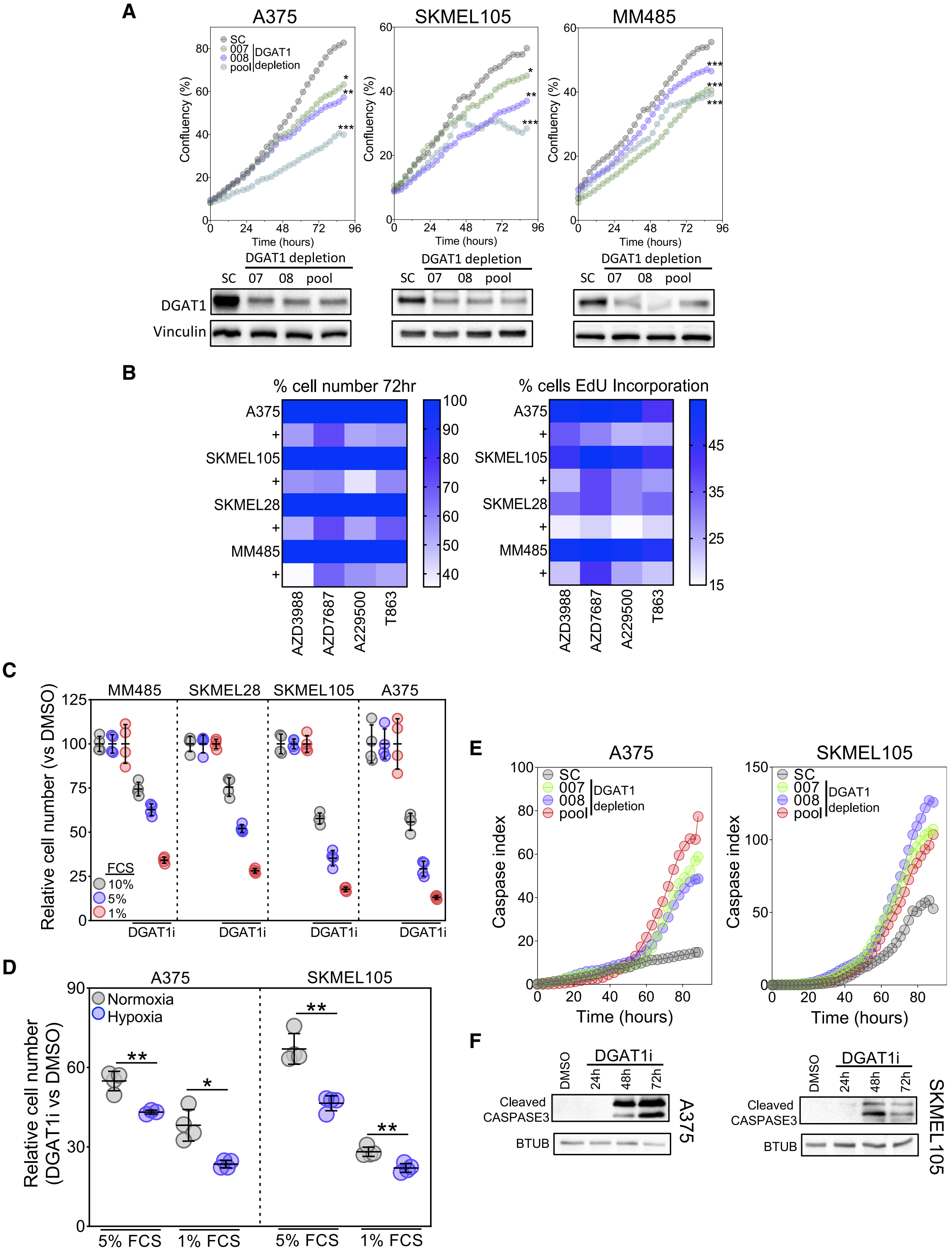

Figure 3. DGAT1 antagonism decreases melanoma cell proliferation and survival

(A) Confluence of cell lines transfected with DGAT1 targeting (007, 008, pool) or scrambled (sc) siRNAs (mean, n = 3, top). Corresponding protein expression of DGAT1 (bottom).

(B) Left: relative cell number (mean, n > 3) in indicated cell lines determined by crystal violet following 72-h DGAT1 inhibitor treatment (50 μM AZD3988, 30 μM A922500, 50 μM AZD7687, or 70 μM T863). Right: percentage of cells in S-phase by using EdU incorporation following 24-h DGAT1 inhibitor treatment (mean, n > 3).

(legend continued on next page)

NRAS^{G12D} EGFP-expressing zebrafish tumors. This analysis revealed increased concentrations of almost all TAG species detected in tumors with forced Dgat1a expression, with longer polyunsaturated FA (PUFA) chains showing the greatest increases (Figures 4A and S4A and Table S2), although changes in individual species were not deemed statistically significant after correction for multiple testing. Several factors confounded the detection of lipid species changes in Dgat1a-over-expressing tumors. First, EGFP-expressing tumors also over-express Dgat1a and have ample LD (Henderson et al., 2019). Second, lipids originating from associated stromal cells dilute the lipids derived from tumor cells.

In order to overcome the complexity of whole-tumor analysis, we turned to melanoma cell lines. Over-expression of DGAT1 in human melanoma cells resulted in a striking increase in LD (Figure 4B), consistent with cells adopting an engorged morphology (Figure 4B). Moreover, this was also observed in NHM upon DGAT1 over-expression (Figure S4B). In parallel, UHPLC-MS lipidomic analysis revealed an increase in almost all TAG species (Figure 4B and Tables S3 and S4), corroborating the trend in TAG seen in zebrafish tumors over-expressing Dgat1a (Figure 4A). Conversely, antagonism or depletion of DGAT1 in melanoma cell lines over-expressing endogenous DGAT1 led to a reduction in the amount of LD beginning between 12 and 24 h and continuing until 72 h (Figures 4C and S4C), as previously reported for glioblastoma cells (Cheng et al., 2020) and other cell types (Nguyen et al., 2017). In contrast, LD were not affected by DGAT2 depletion (Figure S4D). As expected, UHPLC-MS lipidomic analysis revealed a reduction of multiple TAG species already at 24 h that was maintained until 72 h, (Figures 4D and S4E and Tables S5 and S6), with TAG-containing PUFA particularly affected (Figure S4A).

Alongside the anticipated changes in TAG levels following manipulation of DGAT1 activity, we also observed a decrease in several acylcarnitine (AcCa) species upon DGAT1 over-expression (Figure 4B and Tables S3 and S4) and a reciprocal increase in AcCa species following DGAT1 inhibition (Figures 4D, 4E, and S4E and Tables S5 and S6). AcCa, formed by esterification of FA (typically long chain) to L-carnitine, are transported into mitochondria to be processed into acetyl-CoA through β-oxidation. The production of AcCa determines the rate of FAO (Falomir-Lockhart et al., 2019). Moreover, excessive FAO can result in mitochondrial dysfunction (Koves et al., 2008). Indeed, we observed impaired ATP production following DGAT1 inhibition, implied by the increased phosphorylation of both AMPK and RAPTOR (Figure 4F). Furthermore, we observed reduced oxygen consumption in DGAT1-inhibited cells after 48 h (Figure 4G), implying decreased mitochondrial respiratory function despite levels of AcCa remaining elevated (Figures 4D and S5E). Additionally, post-24-h DGAT1 inhibition, we observed loss of mitochondrial membrane potential, decreased PINK1 cleavage, and increased levels of mitophagy factor PARKIN (Figure 4H). Moreover, by blocking AcCa synthesis using etomoxir (a carnitine palmitoyltransferase inhibitor), we could partially restore mitochondrial membrane potential and cell proliferation (Figures S4F and S4G). Taking these results together, we conclude that DGAT1 maintains mitochondrial function in melanoma cells by regulating the availability of FA for FAO.

DGAT1 promotes survival of melanoma cells through suppressing ROS generation

To better understand the effects of DGAT1 inhibition on cellular signaling and metabolism, we performed MS-based whole-proteome analysis in SILAC-labeled A375 cells. Analysis of differentially expressed proteins highlighted three key areas: (1) FAO (consistent with increased AcCa availability), (2) peroxisome proliferator-activated receptor (PPAR) signaling, presumably indicating the increased availability of lipid regulators of PPAR transcription factors, and (3) nuclear regulatory factor 2 (NRF2) signaling, which is a well-established response to ROS production that dampens ROS-mediated cellular damage (Sajadimajd and Khazaei, 2018) (Figures 5A, S5A, and S5B). Quantitative gene expression analysis further corroborated the effects of DGAT1 inhibition on these three key biological processes (Figure 5B).

To measure the impact of DGAT1 suppression on ROS generation, we stained cells with fluorescent probes whose emission is changed by ROS. We observed increasing ROS production over time upon DGAT1 inhibition in multiple melanoma cell lines (Figures 5C and S5C). We also observed an increase specifically in mitochondrial ROS upon both DGAT1 inhibition and depletion (Figures S5D and S5E), a predicted consequence of excessive FAO (Koves et al., 2008). Through a chain reaction, oxygen-centered ROS can yield highly reactive and cytotoxic lipid peroxides responsible for ferroptosis (Conrad and Pratt, 2019), a form of programmed cell death. Accordingly, DGAT1 inhibition led to an increase in lipid peroxidation from 24 h, with further increases by 48 h both in the cytoplasm (Figure 5D) and in the mitochondria specifically (Figure 5E). Additionally, depletion of DGAT1 resulted in increased protein conjugation with 4-hydroxynonenal (4HNE) (Figure 5F), a lipid peroxidation by-product (Dalleau et al., 2013). Conversely, we observed decreased 4HNE protein conjugation in DGAT1-over-expressing melanoma cells (Figure 5G) and in zebrafish tumors over-expressing Dgat1a (Figure 5H), indicating that lipid peroxidation and its suppression by DGAT1 occurs not only in cell lines but also within tumors.

We next investigated whether ROS induction upon DGAT1 suppression affected melanoma cell survival. Introduction of the ROS-scavenging agents Tempol and Ebselen partially

(C) Relative cell number determined by crystal violet staining following 72-h A922500 treatment with cells grown in varying concentrations of fetal calf serum (FCS; mean ± SD, n > 3).
(D) Relative cell number determined by crystal violet staining following 48-h A922500 treatment under normoxic or hypoxic conditions (1% O_2) with cells grown in varying concentrations of FCS (relative to DMSO control for each condition; mean ± SD, n > 3).
(E) Cleaved caspase index in indicated cell lines following transfection with either a DGAT1-targeting siRNA (007, 008, pool) or a sc control (mean, n = 3).
(F) Protein expression of cleaved caspase-3 following treatment with/without A922500 for 24–72 h.
(A) and (D) For significance: *p < 0.05, **p < 0.01, and ***p < 0.001.

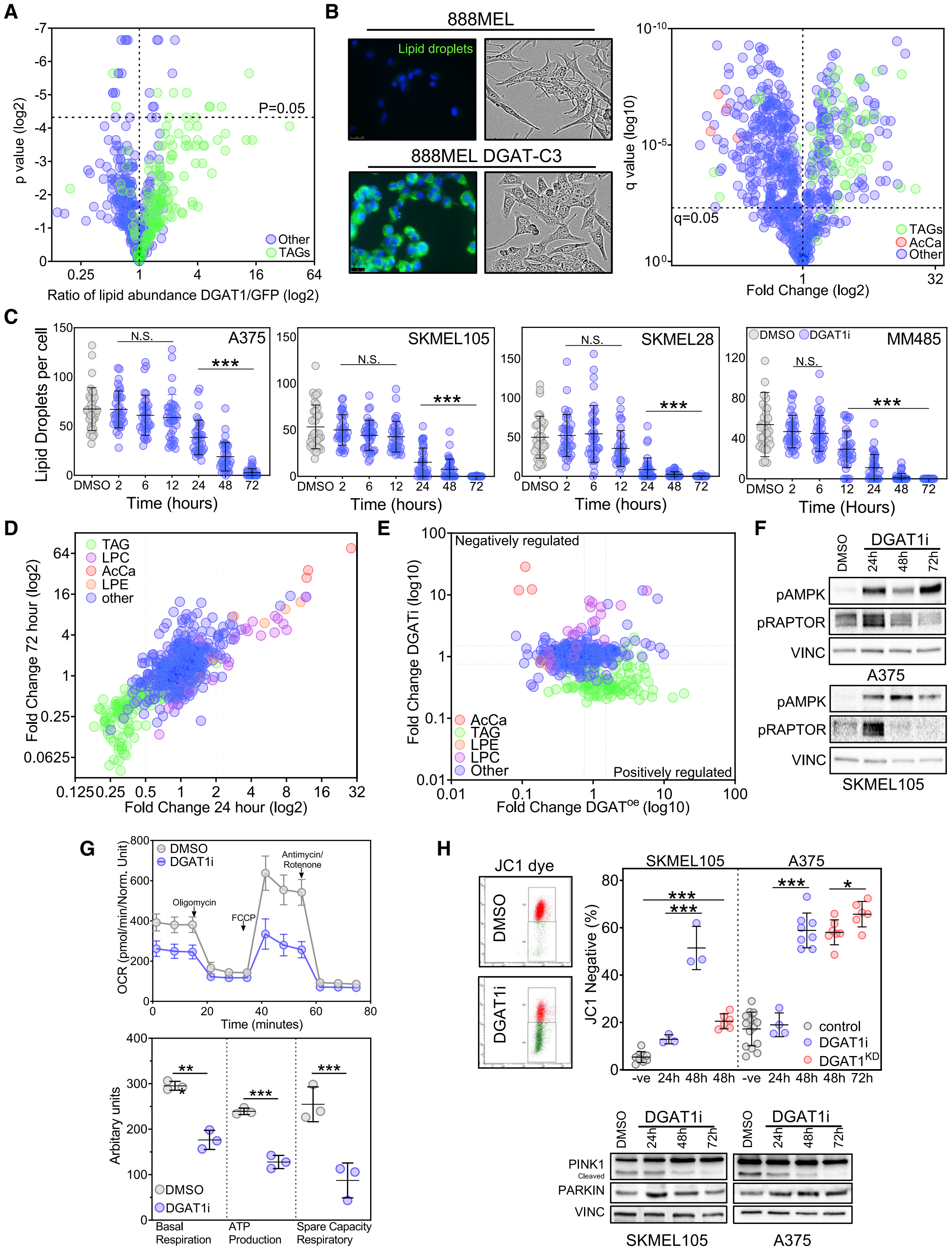

Figure 4. DGAT1-formed lipid droplets act as caretakers of mitochondrial health

(A) Lipidomic profiling using UHPLC-MS of NRASG12D-positive EGFP-expressing (n = 6) and NRASG12D-positive Dgat1a-over-expressing (n = 6) tumors showing the ratio of individual lipid species annotated by MS/MS.

(B) Representative images of 888MEL and Clone 3 cells stained with BODIPY (left; scale bar, 10 μm). Brightfield images of 888MEL parental cells and Clone 3 DGAT1-over-expressing cells (middle). UHPLC-lipidomic analysis of 888MEL parental and Clone 3 DGAT1-over-expressing cells. Fold change relative to 888MEL parental cells (right) (all conditions n = 3). TAG, triacylglycerides; AcCa, acylcarnitine.

(C) Number of lipid droplets per cell visualized using BODIPY staining following AZD3988 (DGAT1i) treatment (mean ± SD, n > 30).

(legend continued on next page)

suppressed apoptosis induced by DGAT1 depletion (Figure 5I). Conversely, stable over-expression of DGAT1 was protective against ROS-mediated cell death triggered by chemical ROS inducers (Figure S5F). Moreover, ferrostatin-1, a potent ferroptosis inhibitor (Skouta et al., 2014), rescued the reduction in cell number observed upon DGAT1 inhibition, an effect also observed with Tempol and Ebselen. Combining ferrostatin-1 with either Tempol or Ebselen led to the greatest rescue of cell number and further suppressed apoptosis driven by DGAT1 inhibition (Figure S5G). Thus, DGAT1 promotes the survival of melanoma cells by suppressing the ROS production and lipid peroxidation that would otherwise occur as a result of their increased FA acquisition.

Combined DGAT1 and SOD1 inhibition halts tumor growth

There are few pan-cancer-druggable metabolic oncoproteins whose suppression is well tolerated. We wished to establish whether pharmacological inhibition of DGAT1 would be a viable strategy to halt tumor growth in mice. We implanted DGAT1^{High} A375 melanoma cells in mouse flanks and commenced daily oral treatment with the DGAT1 inhibitor A922500 (90 mg/kg/day) when tumors reached approximately 100 mm^3. Surprisingly, A922500 treatment had no significant impact upon tumor growth *in vivo* (Figure 6A) despite an increase in tumor ROS levels revealed by increased 4HNE conjugation to protein (Figure S6A). We hypothesized that this might be due to more effective ROS neutralization *in vivo*, which would need to be antagonized in order to suppress tumor growth. We therefore investigated expression of the ROS-detoxifying enzymes SOD1 and -2, two known NRF2 target genes (Dreger et al., 2010; Ma et al., 2021; Park and Rho, 2002; Taylor et al., 2008). Indeed, inhibition of DGAT1 in melanoma cell lines led to increased SOD1 and -2 as well as SESTRIN2 (SESN2) (Figure S6B), a NRF2 activator induced upon mitochondrial respiratory malfunction (Bae et al., 2013; Garaeva et al., 2016), in agreement with our earlier MS-based whole-proteome and quantitative PCR analyses demonstrating activation of NRF2 signaling following DGAT1 inhibition (Figures 5A and 5B). To confirm the role of SESN2/NRF2 signaling in inducing SOD expression following DGAT1 inhibition, we depleted SESN2 using siRNA and in parallel treated cells with NRF2 inhibitor ML385 (Singh et al., 2016). These treatments prevented the induction of SOD1, SOD2 and an additional established NRF2 target, HMOX1, by DGAT1 inhibition (Figures S6C–S6F), consistent with SOD induction being driven through a SESN2/NRF2-signaling axis. Moreover, further analysis of the A375 tumors treated with A922500 revealed increased SESN2, SOD1, and SOD2 expression (Figure S6A), concomitant with increased lipid peroxidation, confirming that our A922500 administration had been effective in inducing ROS and NRF2 signaling in tumor cells *in vivo*.

Assuming that ROS-neutralizing factors such as SOD enzymes were limiting the tumor inhibitory effects of DGAT1 antagonism, we next examined whether blocking the induction of SOD1 would augment the impact of DGAT1 inhibition on melanoma cell growth and survival. Indeed, we found that combining DGAT1 inhibition with SOD1 knockdown led to increased ROS generation concomitant with a dramatic reduction in cell number when compared with inhibition of DGAT1 alone (Figures S6G–S6I). In the clinic, tetrathiomolybdate (TTM) anions are used to chelate cupric cations and have been shown to potently inhibit SOD1, which uses cupric cations as a co-factor, leading to clinical trials of TTM as anti-cancer agents (Juarez et al., 2006; Lowndes et al., 2008; Redman et al., 2003). Again, compared with DGAT1 inhibitor treatment alone, the combination of DGAT1 inhibitor and TTM led to a significant increase in ROS generation and 4HNE protein conjugates (Figures 6B and S6J), concomitant with a marked decrease in cell growth and induction of apoptosis (Figures 6B and S6K). We next assessed the impact of combining inhibition of DGAT1 and SOD1 on tumor growth by using not only the BRAF^{V600E}-mutant A375 xenograft model but also an NRAS^{Q61R}-mutant MM485 xenograft model. In both models, similarly to DGAT1 inhibition alone, SOD1 inhibitor treatment alone had no significant impact on tumor growth (Figures 6C and 6D). Strikingly, combined DGAT1 and SOD1 inhibition led to a profound reduction in tumor growth, with most animals demonstrating tumor regression in the MM485 xenograft model (Figures 6C and 6D). Western blotting of protein extracts from tumors revealed the expected increase in 4HNE protein conjugates in tumors treated with DGAT1 inhibitor or SOD1 inhibitor alone, and elevated SOD1 expression following treatment with DGAT1 inhibitor alone. Significantly, 4HNE protein conjugates were most elevated in tumors treated with a combination of the DGAT1 and SOD1 inhibitors (Figures 6E and 6F). Thus, combinatorial DGAT1 and SOD1 inhibition synergized to create a toxic overload of ROS in tumors, severely retarding the growth of melanoma cells *in vivo*.

DISCUSSION

The role of dysregulated lipid metabolism, encompassing more than just *de novo* FA synthesis, in promoting neoplasia is increasingly apparent (Guri et al., 2017; Nomura et al., 2010; Watt et al., 2019). Previously, we demonstrated that enhanced FA uptake accompanies neoplastic progression in melanocytes (Henderson et al., 2019), and this was shown to be a metabolic

(D) UHPLC-lipidomic analysis of SKMEL105 cells following A922500 treatment. Fold change relative to DMSO (all conditions n = 3). TAG, triacylglycerides; LPC, lysophosphatidycholine; LPE, lysophosphatidylethanolamine.

(E) Lipid species fold changes in SKMEL105 following 72-h A922500 treatment plotted versus lipid species fold changes observed in Clone 3 cells.

(F) Protein expression of phospho-AMPK and phospho-RAPTOR following A922500 (DGAT1i) treatment.

(G) Oxygen consumption rate in A375 cells following 48-h A922500 treatment (top). Basal respiration, ATP production, and spare respiratory capacity were calculated (bottom) (mean ± SD, n = 3).

(H) Staining with JC-1 dye following A922500 treatment or following transfection with DGAT1 targeting siRNA. The percentage of cells that lost red aggregates was calculated by using 1 μM CCP as a positive control and comparing this with untreated cells to create two populations of cells for flow cytometry analysis (top; mean ± SD, n > 3). Protein expression of PINK1 and PARKIN following A922500 treatment (bottom).

(C, G, and H) For significance: *p < 0.05, **p < 0.01, and ***p < 0.001.

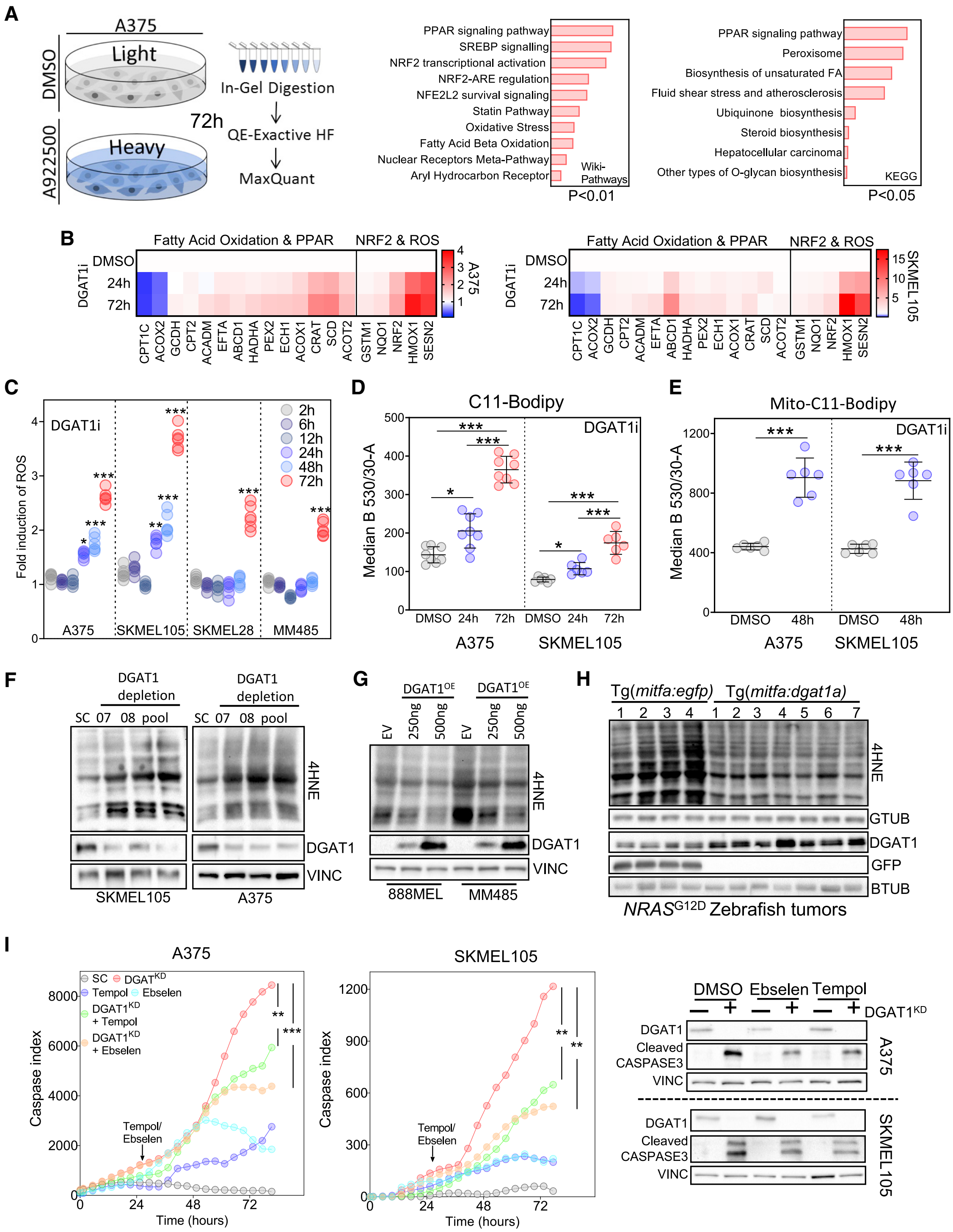

Figure 5. DGAT1 promotes survival of melanoma cells through suppressing ROS generation

(A) Total proteomics workflow (left). GEO of up-regulated proteins (114) ranked by combined score (wikipathways) or log-adjusted p values (metascape, right). (DMSO n = 3, A922500 n = 3).

(B) RT-qPCR analysis following A922500 treatment. Fold change relative to DMSO (mean, n = 3).

(C) ROS levels quantified using dihydroethidium (DHE) fluorescence following A922500 (DGAT1i) treatment. Fold change relative to DMSO (n > 4).

(D) C11-Bodipy staining following A922500 treatment. Mean fluorescence determined using FACS (mean ± SD, n > 6).

(E) Mito-C11-Bodipy staining following A922500 treatment. Mean fluorescence determined using FACS (mean ± SD, n > 6).

(legend continued on next page)

feature of tumor-initiating cells in particular (Pascual et al., 2017) and to drive melanoma tumor development, dissemination, and resistance to targeted therapy (Alicea et al., 2020; Zhang et al., 2018). However, the adaptations allowing melanoma cells to tolerate exposure to excess FA had been overlooked. We now demonstrate that DGAT1 is a *bona fide* metabolism oncoprotein that stimulates melanoma tumorigenesis by conferring protection against ROS, including lipid peroxidation, through inducing LD formation (see model; Figure 7).

DGAT2, a functionally related albeit structurally distinct enzyme, appeared less important than DGAT1 for melanoma LD formation, cell growth, and survival, as was shown previously to be the case for glioblastoma cells (Cheng et al., 2020). This distinction in oncopotency may reflect the distinct roles performed by DGAT1 and DGAT2 in LD formation and adipocyte biology: DGAT1 is exclusively ER resident and responsible for initiating LD formation (Wilfling et al., 2013) and for protecting ER membranes in adipocytes against lipotoxicity (Chitraju et al., 2019); DGAT2 can relocate to LD from the ER and is responsible for further enlarging LD when FA are abundant (Wilfling et al., 2013). Moreover, whereas *Dgat1*-null mice are viable, *Dgat2*-null mice die at birth lacking >90% of TAG (Stone et al., 2004).

We uncovered frequent *DGAT1* amplification and up-regulation in melanoma but also in many other cancers, notably ovarian, breast, uterine, esophageal, liver, pancreatic, head and neck, prostate, stomach, and lung cancers. Abundant LD have been observed in a range of cancers, consistent with widespread up-regulation of DGAT1 (Cheng et al., 2020; Cruz et al., 2020; Petan et al., 2018), indicating that DGAT1 is likely to perform an oncogenic role in these other cancers too. The co-occurrence of *DGAT1* amplification with *FASN*, *CD36*, or *MAGL* amplification in human cancers was significant, arguing for these co-aberrations being synergistic, in keeping with DGAT1 facilitating safe accumulation of FA in cancer cells.

We propose that FA sequestration as TAG in LD allows melanoma cells to accumulate and utilize FA safely, thereby avoiding cell death. As previously described in non-transformed embryonic fibroblasts (Nguyen et al., 2017) and glioblastoma cells (Cheng et al., 2020), we observed that DGAT1 suppression resulted in overloading of mitochondria with AcCa, driving excessive FAO and ROS production. Thus, the pathophysiological role of DGAT1 is to regulate the supply of FA to mitochondria within tolerable limits. Evidently, cancer cells, having elevated FA, require more DGAT1 to achieve this. Lipid peroxides are especially cytotoxic and are generated by the action of oxygen-centered radicals on PUFA preferentially (Gaschler and Stockwell, 2017). As PUFA are concentrated in LD, where they are shielded from peroxidation (Jarc et al., 2018), enhanced LD levels mediated by DGAT1 up-regulation confer dual protection from cell death, both by moderating FAO-triggered ROS production and by simultaneously suppressing PUFA peroxidation.

LD are known to protect non-transformed and transformed cells against a range of cellular stresses typically encountered in the tumor microenvironment, including nutrient deprivation and hypoxia (Cruz et al., 2020; Petan et al., 2018). Indeed, these conditions induce LD formation downstream of autophagy (Nguyen et al., 2017). Additionally, low pH up-regulates DGAT1 and consequently increases LD formation. In turn, LD are required for acidosis-induced metastasis (Corbet et al., 2020). Nutrient deprivation and hypoxia compromise *de novo* FA synthesis and desaturation. Unsaturated FA are essential for membrane fluidity and cell viability, and in the absence of desaturation reactions or the ability to scavenge unsaturated FA from the circulation, cancer cells must draw on LD for unsaturated FA in order to survive (Ackerman et al., 2018). The above roles for LD are consistent with our observation that the effect of DGAT1 suppression on cell growth was exacerbated by serum withdrawal and hypoxia and explains why conversely DGAT1 over-expression conferred its greatest growth advantage under these conditions.

The ability of DGAT1 inhibitor alone to suppress tumor growth was recently shown in a glioblastoma xenograft model (Cheng et al., 2020). However, we did not observe efficacy of DGAT1 inhibition alone in our mouse melanoma xenograft models, most likely due to a striking upregulation of the anti-ROS response. In order to antagonize this adaptation, we employed TTM, a clinically relevant SOD1 inhibitor. Combinatorial DGAT1 and SOD1 inhibition led to a profound impairment of melanoma growth *in vivo*. DGAT1 is pan-cancer amplified, and it remains to be determined to what extent other cancers can mount the anti-ROS responses we observed in our melanoma xenograft models. Thus, future studies should focus on evaluating the efficacy of DGAT1 inhibition alone, or in combination with inhibition of anti-ROS mechanisms, to induce the most well-tolerated and beneficial outcomes for cancer patients.

Limitations of the study

Our study relied on established human melanoma cell lines to explore DGAT1 function, and these may have adapted to *ex vivo* culture in ways that make them less representative than primary cells. To antagonize DGAT1 in cell cultures, we mostly used 30 μM A922500 to ensure complete inhibition over the 24–72 h required to fully deplete LD and to suppress cell viability. Although in excess of doses required to inhibit transiently DGAT1 catalytic activity, this concentration is still beneath that required

(F) 4-Hydroxynonenal (4HNE) protein conjugate abundance and protein expression of DGAT1 following transfection with either DGAT1-targeting siRNA or a scrambled control for 48 h.

(G) 4HNE protein conjugate abundance and protein expression of DGAT1 following transfection with either DGAT1 over-expression vector or an empty vector control for 48 h.

(H) 4HNE protein conjugate abundance and protein expression of Dgat1 and GFP in NRAS^{G12D}-positive EGFP-expressing (n = 4) and NRAS^{G12D}-positive Dgat1a-over-expressing (n = 7) tumors.

(I) Cleaved-caspase index following transfection of a DGAT1-targeting or scrambled siRNA. At 24 h, cells were treated with/without Tempol or Ebselen (mean, n = 3, outer). Corresponding protein expression of DGAT1 and cleaved caspase-3 (inner).

(C–E and I) For significance: *p < 0.05, **p < 0.01, and ***p < 0.001.

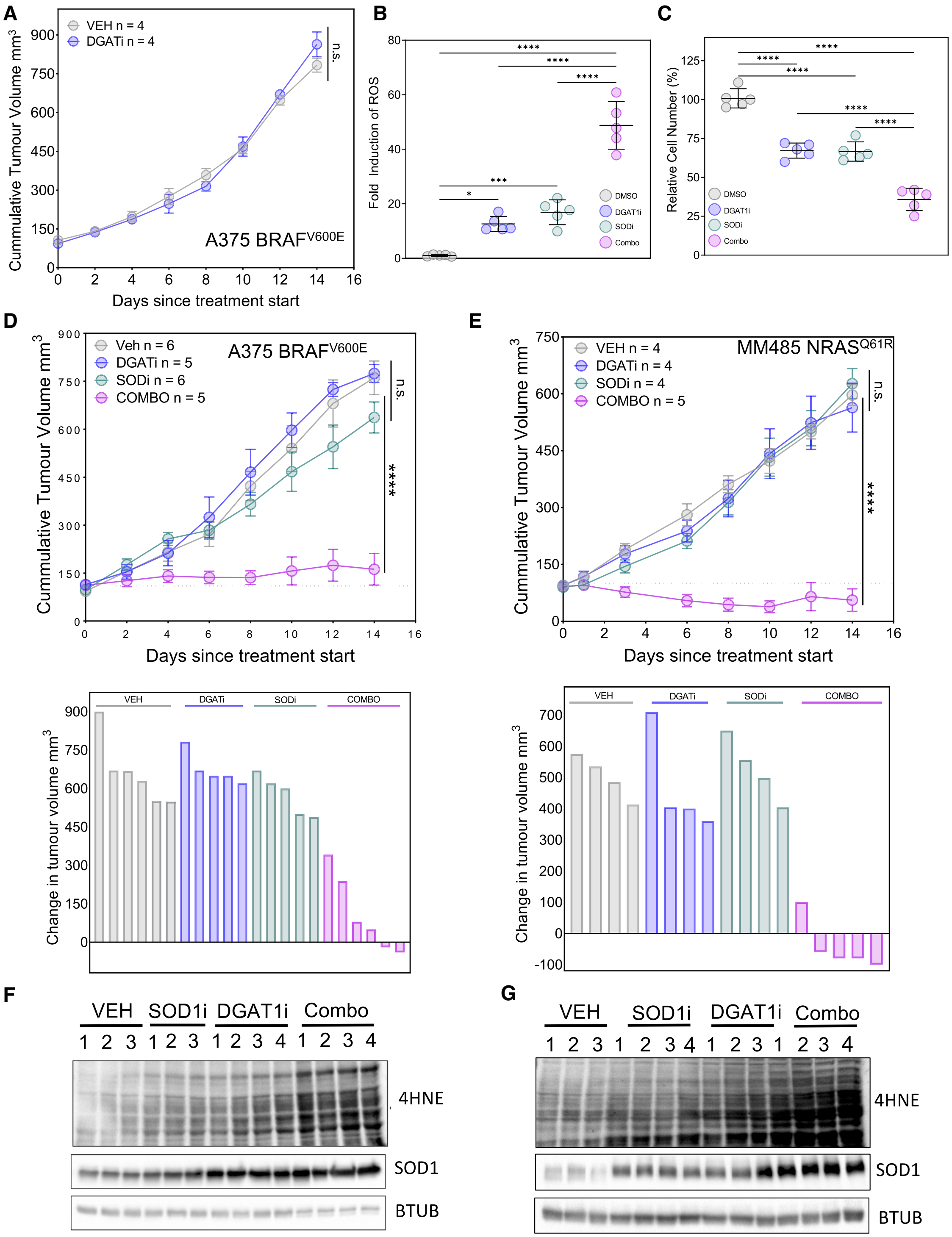

Figure 6. Combined DGAT1 and SOD1 inhibition halts tumor growth

(A) Nude mice bearing A375 tumors were treated (*n* = number of mice per group) with vehicle or A922500 (DGAT1i; 90 mg/kg/day) for 14 days. Data are presented as mean tumor volumes ±SD.

(B) Quantification of ROS levels by DHE fluorescence in A375 cells following treatment with either A922500 or ATN-224 (SOD1i) alone or in combination (combo). Fold change relative to DMSO (mean ± SD, n = 5).

(C) Relative number of A375 cells following treatment with either A922500 or ATN-224 alone or in combination. Fold change relative to DMSO (mean ± SD, n = 5).

(legend continued on next page)

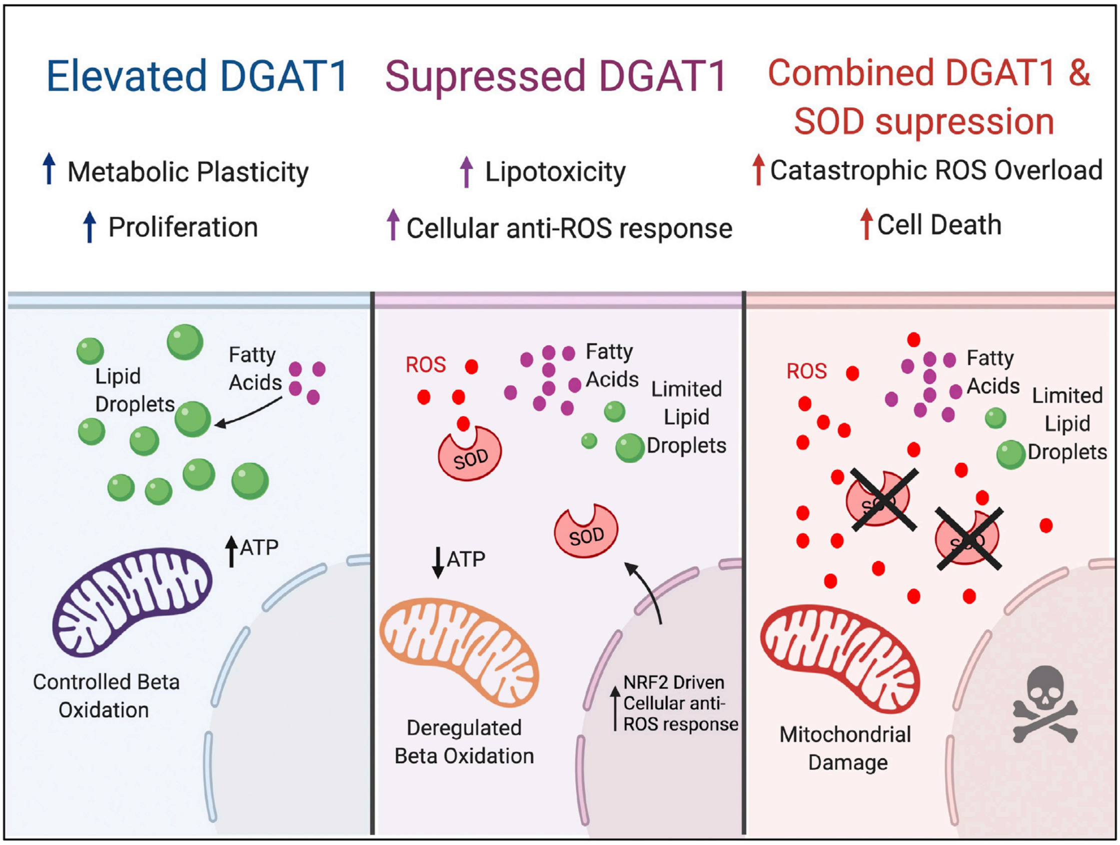

Figure 7. Model

Elevation of DGAT1 allows sequestration of fatty acids as triacylglycerides in lipid droplets and a controlled rate of fatty acid oxidation in mitochondria. If DGAT1 is suppressed, then fatty acids are oxidized, and cytotoxic reactive oxygen species are generated. However, these are neutralized through elevation of SOD1. However, if SOD1 is also suppressed, then the build-up of ROS is intolerable, and cells die.

to suppress the activity of related acyltransferases including DGAT2 (King et al., 2009). Moreover, an even higher dose of A922500 (47 μM) was unable to suppress LD dependent on DGAT2 or to markedly suppress viability in glioblastoma cells over-expressing DGAT2 (Cheng et al., 2020), consistent with A922500 being a selective DGAT1 inhibitor at this concentration. That we can reproduce the effects of A922500 on LD depletion and cell viability with other DGAT1 inhibitors and with siRNA targeting DGAT1 and observe the converse upon over-expressing DGAT1 further corroborate that the effects of A922500 are DGAT1 dependent. To demonstrate the interaction between DGAT1 and SOD1 on tumor growth *in vivo*, we have again relied on pharmacological agents that may have generated off-target effects that contributed to tumor growth inhibition. Additionally, durability of tumor growth suppression was not established in our study, nor host tolerance to chronic drug treatment, albeit that treatment was well tolerated by mice over the time course it was evaluated. As a further limitation, xenograft models do not fully recapitulate features of spontaneous tumors in notably developing in the absence of adaptive immunity. Potentially, PDX models, autochthonous genetically engineered mouse melanoma models, or *ex vivo* tumor explant culture might have allowed us to maintain more features of melanoma in which to assess the impact of DGAT1 depletion or antagonism.

STAR★METHODS

Detailed methods are provided in the online version of this paper and include the following:

- KEY RESOURCE TABLE
- RESOURCE AVAILABILITY
  - Lead contact
  - Materials availability

(D and E) Nude mice bearing A375 tumors (D) or MM485 tumors (E) were treated (n = number of mice per group) with vehicle, A922500 (90 mg/kg/day) or TTM (SODi; 5 mg/kg/day) alone or in combination for 14 days. Data are presented as mean tumor volumes ±SD (top) or change in individual tumor volume (bottom).
(F and G) 4HNE protein conjugate abundance and protein expression of SOD1 in A375 tumors (F) or MM485 tumors (G) treated with vehicle, A922500, or TTM alone or in combination.
(B–E) For significance: *$p < 0.05$, ***$p < 0.001$, and ****$p < 0.0001$.

- Data and code availability
- EXPERIMENTAL MODEL AND SUBJECT DETAILS
  - Zebrafish models
  - Mouse xenograft assays
  - Cell lines
- METHOD DETAILS
  - Compounds and antibodies
  - Plasmid cloning and transfection & siRNA transfection
  - Viral transduction
  - Protein lysate preparation and western blotting
  - Lipid droplet staining and image analysis
  - RNA isolation and real-time PCR analysis
  - Flow cytometry
  - Proliferation assays
  - Dihydroethidium assay
  - Cancer bioinformatics
- QUANTIFICATION AND STATISTICAL ANALYSIS
  - Proteomics
  - Lipidomics

SUPPLEMENTAL INFORMATION

Supplemental information can be found online at https://doi.org/10.1016/j.celrep.2022.110995.

ACKNOWLEDGMENTS

We thank staff of the Biological Mass Spectrometry facility, Bio-imaging facility, Biological Services facility, and Flow facility (all FBMH, UoM) for experimental support. We thank Olivia Sloss and Angeliki Malliri for critically reading the manuscript. A.H. was funded by grant ERC-2011-StG-282059 from the ERC, grant 610262 from the Melanoma Research Alliance, and a PhD studentship A23251 from Cancer Research UK to support D.J.W. C.F. was supported by the Wellcome Trust (Sir Henry Dale fellowship grant number 107636/Z/15/Z) and Biotechnology and Biological Sciences Research Council (grant number BB/R015864/1). The Medical Research Council (MRC) funded the Phenome Centre Birmingham (MR/M009157/1).

AUTHOR CONTRIBUTIONS

Cell-line-based experimentation: D.J.W., M.P.S., R.O., and J.H. Zebrafish models: A.P.B., D.J.W., H.J., M.G., and C.C. Mouse experiments: D.J.W., C.W.W., and B.A.T. Proteomics: M.P.S., D.J.W., P.F., and C.F. Lipidomics: D.J.W., M.P.S., A.D.S., A.J., G.R.L., and W.B.D. Bio-informatics analysis: D.J.W., J.W., H.F., C.F., and M.P.S. Experimental design: D.J.W., A.P.B., C.F., M.P.S, and A.H. Sample acquisition and cell lines: P.L., C.W., M.P.S., and A.H. Conceptualization: A.P.B., M.P.S., and A.H. Drafting of manuscript: D.J.W., A.P.B., M.P.S., and A.H. Revisions by all authors. Acquisition of funds: C.W., C.F., and A.H. Study supervision: M.P.S. and A.H.

DECLARATION OF INTERESTS

The authors declare no competing interests.

Received: September 19, 2021
Revised: March 30, 2022
Accepted: June 1, 2022
Published: June 21, 2022

REFERENCES

Ackerman, D., and Simon, M.C. (2014). Hypoxia, lipids, and cancer: surviving the harsh tumor microenvironment. Trends Cell Biol. *24*, 472–478. https://doi.org/10.1016/j.tcb.2014.06.001.

Ackerman, D., Tumanov, S., Qiu, B., Michalopoulou, E., Spata, M., Azzam, A., Xie, H., Simon, M.C., and Kamphorst, J.J. (2018). Triglycerides promote lipid homeostasis during hypoxic stress by balancing fatty acid saturation. Cell Rep. *24*, 2596–2605.e5. https://doi.org/10.1016/j.celrep.2018.08.015.

Alicea, G.M., Rebecca, V.W., Goldman, A.R., Fane, M.E., Douglass, S.M., Behera, R., Webster, M.R., Kugel, C.H., 3rd, Ecker, B.L., Caino, M.C., et al. (2020). Changes in aged fibroblast lipid metabolism induce age-dependent melanoma cell resistance to targeted therapy via the fatty acid transporter FATP2. Cancer Discov. *10*, 1282–1295. https://doi.org/10.1158/2159-8290.Cd-20-0329.

Anaya, J. (2016). OncoLnc: linking TCGA survival data to mRNAs, miRNAs, and lncRNAs. Peerj Comput. Sci. *2*, e67. https://doi.org/10.7717/peerj-cs.67.

Andrejeva, G., and Rathmell, J.C. (2017). Similarities and distinctions of cancer and immune metabolism in inflammation and tumors. Cell Metabol. *26*, 49–70. https://doi.org/10.1016/j.cmet.2017.06.004.

Bae, S.H., Sung, S.H., Oh, S.Y., Lim, J.M., Lee, S.K., Park, Y.N., Lee, H.E., Kang, D., and Rhee, S.G. (2013). Sestrins activate Nrf2 by promoting p62-dependent autophagic degradation of Keap1 and prevent oxidative liver damage. Cell Metabol. *17*, 73–84. https://doi.org/10.1016/j.cmet.2012.12.002.

Bastian, B.C., LeBoit, P.E., Hamm, H., Bröcker, E.B., and Pinkel, D. (1998). Chromosomal gains and losses in primary cutaneous melanomas detected by comparative genomic hybridization. Cancer Res. *58*, 2170–2175.

Brookheart, R.T., Michel, C.I., and Schaffer, J.E. (2009). As a matter of fat. Cell Metabol. *10*, 9–12. https://doi.org/10.1016/j.cmet.2009.03.011.

Casar, B., Badrock, A.P., Jiménez, I., Arozarena, I., Colón-Bolea, P., Lorenzo-Martín, L.F., Barinaga-Rementería, I., Barriuso, J., Cappitelli, V., Donoghue, D.J., et al. (2018). RAS at the Golgi antagonizes malignant transformation through PTPRκ-mediated inhibition of ERK activation. Nat. Commun. *9*, 3595. https://doi.org/10.1038/s41467-018-05941-8.

Ceol, C.J., Houvras, Y., Jane-Valbuena, J., Bilodeau, S., Orlando, D.A., Battisti, V., Fritsch, L., Lin, W.M., Hollmann, T.J., Ferré, F., et al. (2011). The histone methyltransferase SETDB1 is recurrently amplified in melanoma and accelerates its onset. Nature *471*, 513–517. https://doi.org/10.1038/nature09806.

Chambers, M.C., Maclean, B., Burke, R., Amodei, D., Ruderman, D.L., Neumann, S., Gatto, L., Fischer, B., Pratt, B., Egertson, J., et al. (2012). A cross-platform toolkit for mass spectrometry and proteomics. Nat. Biotechnol. *30*, 918–920. https://doi.org/10.1038/nbt.2377.

Chen, E.Y., Tan, C.M., Kou, Y., Duan, Q., Wang, Z., Meirelles, G.V., Clark, N.R., and Ma'ayan, A. (2013). Enrichr: interactive and collaborative HTML5 gene list enrichment analysis tool. BMC Bioinf. *14*, 128. https://doi.org/10.1186/1471-2105-14-128.

Chen, M., and Huang, J. (2019). The expanded role of fatty acid metabolism in cancer: new aspects and targets. Precis. Clin. Med. *2*, 183–191. https://doi.org/10.1093/pcmedi/pbz017.

Cheng, X., Geng, F., Pan, M., Wu, X., Zhong, Y., Wang, C., Tian, Z., Cheng, C., Zhang, R., Puduvalli, V., et al. (2020). Targeting DGAT1 ameliorates glioblastoma by increasing fat catabolism and oxidative stress. Cell Metabol. *32*, 229–242.e8. https://doi.org/10.1016/j.cmet.2020.06.002.

Chitraju, C., Walther, T.C., and Farese, R.V., Jr. (2019). The triglyceride synthesis enzymes DGAT1 and DGAT2 have distinct and overlapping functions in adipocytes. J. Lipid Res. *60*, 1112–1120. https://doi.org/10.1194/jlr.M093112.

Conrad, M., and Pratt, D.A. (2019). The chemical basis of ferroptosis. Nat. Chem. Biol. *15*, 1137–1147. https://doi.org/10.1038/s41589-019-0408-1.

Corbet, C., Bastien, E., Santiago de Jesus, J.P., Dierge, E., Martherus, R., Vander Linden, C., Doix, B., Degavre, C., Guilbaud, C., Petit, L., et al. (2020). TGFβ2-induced formation of lipid droplets supports acidosis-driven EMT and the metastatic spreading of cancer cells. Nat. Commun. *11*, 454. https://doi.org/10.1038/s41467-019-14262-3.

Cox, J., Neuhauser, N., Michalski, A., Scheltema, R.A., Olsen, J.V., and Mann, M. (2011). Andromeda: a peptide search engine integrated into the MaxQuant environment. J. Proteome Res. *10*, 1794–1805. https://doi.org/10.1021/pr101065j.

Cruz, A.L.S., Barreto, E.d.A., Fazolini, N.P.B., Viola, J.P.B., and Bozza, P.T. (2020). Lipid droplets: platforms with multiple functions in cancer hallmarks. Cell Death Dis. *11*, 105. https://doi.org/10.1038/s41419-020-2297-3.

Dalleau, S., Baradat, M., Guéraud, F., and Huc, L. (2013). Cell death and diseases related to oxidative stress: 4-hydroxynonenal (HNE) in the balance. Cell Death Differ. *20*, 1615–1630. https://doi.org/10.1038/cdd.2013.138.

DeBerardinis, R.J., and Chandel, N.S. (2016). Fundamentals of cancer metabolism. Sci. Adv. *2*, e1600200. https://doi.org/10.1126/sciadv.1600200.

DeVita, R.J., and Pinto, S. (2013). Current status of the Research and development of diacylglycerol O-acyltransferase 1 (DGAT1) inhibitors. J. Med. Chem. *56*, 9820–9825. https://doi.org/10.1021/jm4007033.

Dieterle, F., Ross, A., Schlotterbeck, G., and Senn, H. (2006). Probabilistic quotient normalization as robust method to account for dilution of complex biological mixtures. Application in 1H NMR metabonomics. Anal. Chem. *78*, 4281–4290. https://doi.org/10.1021/ac051632c.

Dreger, H., Westphal, K., Wilck, N., Baumann, G., Stangl, V., Stangl, K., and Meiners, S. (2010). Protection of vascular cells from oxidative stress by proteasome inhibition depends on Nrf2. Cardiovasc. Res. *85*, 395–403. https://doi.org/10.1093/cvr/cvp279.

Ehlers, J.P., Worley, L., Onken, M.D., and Harbour, J.W. (2005). DDEF1 is located in an amplified region of chromosome 8q and is overexpressed in uveal melanoma. Clin. Cancer Res. *11*, 3609–3613. https://doi.org/10.1158/1078-0432.CCR-04-1941.

Falomir-Lockhart, L.J., Cavazzutti, G.F., Giménez, E., and Toscani, A.M. (2019). Fatty acid signaling mechanisms in neural cells: fatty acid receptors. Front. Cell. Neurosci. *13*, 162. https://doi.org/10.3389/fncel.2019.00162.

Fischer, G.M., Vashisht Gopal, Y.N., McQuade, J.L., Peng, W., DeBerardinis, R.J., and Davies, M.A. (2018). Metabolic strategies of melanoma cells: mechanisms, interactions with the tumor microenvironment, and therapeutic implications. Pigment Cell Melanoma Res. *31*, 11–30. https://doi.org/10.1111/pcmr.12661.

Flavin, R., Peluso, S., Nguyen, P.L., and Loda, M. (2010). Fatty acid synthase as a potential therapeutic target in cancer. Future Oncol. *6*, 551–562. https://doi.org/10.2217/fon.10.11.

Francavilla, C., Papetti, M., Rigbolt, K.T.G., Pedersen, A.K., Sigurdsson, J.O., Cazzamali, G., Karemore, G., Blagoev, B., and Olsen, J.V. (2016). Multilayered proteomics reveals molecular switches dictating ligand-dependent EGFR trafficking. Nat. Struct. Mol. Biol. *23*, 608–618. https://doi.org/10.1038/nsmb.3218.

Gao, J., Aksoy, B.A., Dogrusoz, U., Dresdner, G., Gross, B., Sumer, S.O., Sun, Y., Jacobsen, A., Sinha, R., Larsson, E., et al. (2013). Integrative analysis of complex cancer genomics and clinical profiles using the cBioPortal. Sci. Signal. *6*, pl1. https://doi.org/10.1126/scisignal.2004088.

Garaeva, A.A., Kovaleva, I.E., Chumakov, P.M., and Evstafieva, A.G. (2016). Mitochondrial dysfunction induces SESN2 gene expression through activating transcription factor 4. Cell Cycle *15*, 64–71. https://doi.org/10.1080/15384101.2015.1120929.

Gaschler, M.M., and Stockwell, B.R. (2017). Lipid peroxidation in cell death. Biochem. Biophys. Res. Commun. *482*, 419–425. https://doi.org/10.1016/j.bbrc.2016.10.086.

Guri, Y., Colombi, M., Dazert, E., Hindupur, S.K., Roszik, J., Moes, S., Jenoe, P., Heim, M.H., Riezman, I., Riezman, H., and Hall, M.N. (2017). mTORC2 promotes tumorigenesis via lipid synthesis. Cancer Cell *32*, 807–823.e12. https://doi.org/10.1016/j.ccell.2017.11.011.

Henderson, F., Johnston, H.R., Badrock, A.P., Jones, E.A., Forster, D., Nagaraju, R.T., Evangelou, C., Kamarashev, J., Green, M., Fairclough, M., et al. (2019). Enhanced fatty acid scavenging and glycerophospholipid metabolism accompany melanocyte neoplasia progression in zebrafish. Cancer Res. *79*, 2136–2151. https://doi.org/10.1158/0008-5472.CAN-18-2409.

Jarc, E., Kump, A., Malavašič, P., Eichmann, T.O., Zimmermann, R., and Petan, T. (2018). Lipid droplets induced by secreted phospholipase A2 and unsaturated fatty acids protect breast cancer cells from nutrient and lipotoxic stress. Biochim. Biophys. Acta Mol. Cell Biol. Lipids *1863*, 247–265. https://doi.org/10.1016/j.bbalip.2017.12.006.

Juarez, J.C., Betancourt, O., Pirie-Shepherd, S.R., Guan, X., Price, M.L., Shaw, D.E., Mazar, A.P., and Doñate, F. (2006). Copper binding by tetrathiomolybdate attenuates angiogenesis and tumor cell proliferation through the inhibition of superoxide dismutase 1. Clin. Cancer Res. *12*, 4974–4982. https://doi.org/10.1158/1078-0432.CCR-06-0171.

King, A.J., Segreti, J.A., Larson, K.J., Souers, A.J., Kym, P.R., Reilly, R.M., Zhao, G., Mittelstadt, S.W., and Cox, B.F. (2009). Diacylglycerol acyltransferase 1 inhibition lowers serum triglycerides in the Zucker fatty rat and the hyperlipidemic hamster. J. Pharmacol. Exp. Ther. *330*, 526–531. https://doi.org/10.1124/jpet.109.154047.

Koundouros, N., and Poulogiannis, G. (2020). Reprogramming of fatty acid metabolism in cancer. Br. J. Cancer *122*, 4–22. https://doi.org/10.1038/s41416-019-0650-z.

Koves, T.R., Ussher, J.R., Noland, R.C., Slentz, D., Mosedale, M., Ilkayeva, O., Bain, J., Stevens, R., Dyck, J.R.B., Newgard, C.B., et al. (2008). Mitochondrial overload and incomplete fatty acid oxidation contribute to skeletal muscle insulin resistance. Cell Metabol. *7*, 45–56. https://doi.org/10.1016/j.cmet.2007.10.013.

Lee, S.Y., Ju, M.K., Jeon, H.M., Lee, Y.J., Kim, C.H., Park, H.G., Han, S.I., and Kang, H.S. (2018). Oncogenic metabolism acts as a prerequisite step for induction of cancer metastasis and cancer stem cell phenotype. Oxid. Med. Cell. Longev. *2018*, 1027453. https://doi.org/10.1155/2018/1027453.

Lengyel, E., Makowski, L., DiGiovanni, J., and Kolonin, M.G. (2018). Cancer as a matter of fat: the crosstalk between adipose tissue and tumors. Trends Cancer *4*, 374–384. https://doi.org/10.1016/j.trecan.2018.03.004.

Liao, Y., Smyth, G.K., and Shi, W. (2014). featureCounts: an efficient general purpose program for assigning sequence reads to genomic features. Bioinformatics *30*, 923–930. https://doi.org/10.1093/bioinformatics/btt656.

Libiseller, G., Dvorzak, M., Kleb, U., Gander, E., Eisenberg, T., Madeo, F., Neumann, S., Trausinger, G., Sinner, F., Pieber, T., and Magnes, C. (2015). IPO: a tool for automated optimization of XCMS parameters. BMC Bioinf. *16*, 118. https://doi.org/10.1186/s12859-015-0562-8.

Lowndes, S.A., Adams, A., Timms, A., Fisher, N., Smythe, J., Watt, S.M., Joel, S., Donate, F., Hayward, C., Reich, S., et al. (2008). Phase I study of copper-binding agent ATN-224 in patients with advanced solid tumors. Clin. Cancer Res. *14*, 7526–7534. https://doi.org/10.1158/1078-0432.CCR-08-0315.

Lyssiotis, C.A., and Kimmelman, A.C. (2017). Metabolic interactions in the tumor microenvironment. Trends Cell Biol. *27*, 863–875. https://doi.org/10.1016/j.tcb.2017.06.003.

Ma, C.-s., Lv, Q.-m., Zhang, K.-r., Tang, Y.-b., Zhang, Y.-f., Shen, Y., Lei, H.-m., and Zhu, L. (2021). NRF2-GPX4/SOD2 axis imparts resistance to EGFR-tyrosine kinase inhibitors in non-small-cell lung cancer cells. Acta Pharmacol. Sin. *42*, 613–623. https://doi.org/10.1038/s41401-020-0443-1.

Maan, M., Peters, J.M., Dutta, M., and Patterson, A.D. (2018). Lipid metabolism and lipophagy in cancer. Biochem. Biophys. Res. Commun. *504*, 582–589. https://doi.org/10.1016/j.bbrc.2018.02.097.

Mermel, C.H., Schumacher, S.E., Hill, B., Meyerson, M.L., Beroukhim, R., and Getz, G. (2011). GISTIC2.0 facilitates sensitive and confident localization of the targets of focal somatic copy-number alteration in human cancers. Genome Biol. *12*, R41. https://doi.org/10.1186/gb-2011-12-4-r41.

Molenaar, R.J., Maciejewski, J.P., Wilmink, J.W., and van Noorden, C.J.F. (2018). Wild-type and mutated IDH1/2 enzymes and therapy responses. Oncogene *37*, 1949–1960. https://doi.org/10.1038/s41388-017-0077-z.

Nguyen, T.B., Louie, S.M., Daniele, J.R., Tran, Q., Dillin, A., Zoncu, R., Nomura, D.K., and Olzmann, J.A. (2017). DGAT1-Dependent lipid droplet biogenesis protects mitochondrial function during starvation-induced autophagy. Dev. Cell *42*, 9–21.e5. https://doi.org/10.1016/j.devcel.2017.06.003.

Nieman, K.M., Kenny, H.A., Penicka, C.V., Ladanyi, A., Buell-Gutbrod, R., Zillhardt, M.R., Romero, I.L., Carey, M.S., Mills, G.B., Hotamisligil, G.S., et al. (2011). Adipocytes promote ovarian cancer metastasis and provide energy

for rapid tumor growth. Nat. Med. *17*, 1498–1503. https://doi.org/10.1038/nm.2492.

Nomura, D.K., Long, J.Z., Niessen, S., Hoover, H.S., Ng, S.-W., and Cravatt, B.F. (2010). Monoacylglycerol lipase regulates a fatty acid network that promotes cancer pathogenesis. Cell *140*, 49–61. https://doi.org/10.1016/j.cell.2009.11.027.

Olsen, J.V., Blagoev, B., Gnad, F., Macek, B., Kumar, C., Mortensen, P., and Mann, M. (2006). Global, in vivo, and site-specific phosphorylation dynamics in signaling networks. Cell *127*, 635–648. https://doi.org/10.1016/j.cell.2006.09.026.

Park, E.Y., and Rho, H.M. (2002). The transcriptional activation of the human copper/zinc superoxide dismutase gene by 2, 3, 7, 8-tetrachlorodibenzo-p-dioxin through two different regulator sites, the antioxidant responsive element and xenobiotic responsive element. Mol. Cell. Biochem. *240*, 47–55. https://doi.org/10.1023/a:1020600509965.

Parsons, H.M., Ludwig, C., Günther, U.L., and Viant, M.R. (2007). Improved classification accuracy in 1- and 2-dimensional NMR metabolomics data using the variance stabilising generalised logarithm transformation. BMC Bioinf. *8*, 234. https://doi.org/10.1186/1471-2105-8-234.

Pascual, G., Avgustinova, A., Mejetta, S., Martín, M., Castellanos, A., Attolini, C.S.O., Berenguer, A., Prats, N., Toll, A., Hueto, J.A., et al. (2017). Targeting metastasis-initiating cells through the fatty acid receptor CD36. Nature *541*, 41–45. https://doi.org/10.1038/nature20791.

Petan, T., Jarc, E., and Jusović, M. (2018). Lipid droplets in cancer: guardians of fat in a stressful world. Molecules *23*, 1941. https://doi.org/10.3390/molecules23081941.

Redman, B.G., Esper, P., Pan, Q., Dunn, R.L., Hussain, H.K., Chenevert, T., Brewer, G.J., and Merajver, S.D. (2003). Phase II trial of tetrathiomolybdate in patients with advanced kidney cancer. Clin. Cancer Res. *9*, 1666–1672.

Rhodes, D.R., Yu, J., Shanker, K., Deshpande, N., Varambally, R., Ghosh, D., Barrette, T., Pander, A., and Chinnaiyan, A.M. (2004). ONCOMINE: a cancer microarray database and integrated data-mining platform. Neoplasia *6*, 1–6. https://doi.org/10.1016/s1476-5586(04)80047-2.

Sajadimajd, S., and Khazaei, M. (2018). Oxidative stress and cancer: the role of Nrf2. Curr. Cancer Drug Targets *18*, 538–557. https://doi.org/10.2174/1568009617666171002144228.

Schindelin, J., Arganda-Carreras, I., Frise, E., Kaynig, V., Longair, M., Pietzsch, T., Preibisch, S., Rueden, C., Saalfeld, S., Schmid, B., et al. (2012). Fiji: an open-source platform for biological-image analysis. Nat. Methods *9*, 676–682. https://doi.org/10.1038/nmeth.2019.

Schlagbauer-Wadl, H., Griffioen, M., van Elsas, A., Schrier, P.I., Pustelnik, T., Eichler, H.G., Wolff, K., Pehamberger, H., and Jansen, B. (1999). Influence of increased c-Myc expression on the growth characteristics of human melanoma. J. Invest. Dermatol. *112*, 332–336. https://doi.org/10.1046/j.1523-1747.1999.00506.x.

Singh, A., Venkannagari, S., Oh, K.H., Zhang, Y.Q., Rohde, J.M., Liu, L., Nimmagadda, S., Sudini, K., Brimacombe, K.R., Gajghate, S., et al. (2016). Small molecule inhibitor of NRF2 selectively intervenes therapeutic resistance in KEAP1-deficient NSCLC tumors. ACS Chem. Biol. *11*, 3214–3225. https://doi.org/10.1021/acschembio.6b00651.

Skouta, R., Dixon, S.J., Wang, J., Dunn, D.E., Orman, M., Shimada, K., Rosenberg, P.A., Lo, D.C., Weinberg, J.M., Linkermann, A., and Stockwell, B.R. (2014). Ferrostatins inhibit oxidative lipid damage and cell death in diverse disease models. J. Am. Chem. Soc. *136*, 4551–4556. https://doi.org/10.1021/ja411006a.

Smith, C.A., Want, E.J., O'Maille, G., Abagyan, R., and Siuzdak, G. (2006). XCMS: processing mass spectrometry data for metabolite profiling using nonlinear peak alignment, matching, and identification. Anal. Chem. *78*, 779–787. https://doi.org/10.1021/ac051437y.

Stone, S.J., Myers, H.M., Watkins, S.M., Brown, B.E., Feingold, K.R., Elias, P.M., and Farese, R.V., Jr. (2004). Lipopenia and skin barrier abnormalities in DGAT2-deficient mice. J. Biol. Chem. *279*, 11767–11776. https://doi.org/10.1074/jbc.M311000200.

Sumner, L.W., Amberg, A., Barrett, D., Beale, M.H., Beger, R., Daykin, C.A., Fan, T.W.M., Fiehn, O., Goodacre, R., Griffin, J.L., et al. (2007). Proposed minimum reporting standards for chemical analysis chemical analysis working group (CAWG) metabolomics standards initiative (MSI). Metabolomics *3*, 211–221. https://doi.org/10.1007/s11306-007-0082-2.

Taylor, R.C., Acquaah-Mensah, G., Singhal, M., Malhotra, D., and Biswal, S. (2008). Network inference algorithms elucidate Nrf2 regulation of mouse lung oxidative stress. PLoS Comput. Biol. *4*, e1000166. https://doi.org/10.1371/journal.pcbi.1000166.

Venkatesan, A.M., Vyas, R., Gramann, A.K., Dresser, K., Gujja, S., Bhatnagar, S., Chhangawala, S., Gomes, C.B.F., Xi, H.S., Lian, C.G., et al. (2018). Ligand-activated BMP signaling inhibits cell differentiation and death to promote melanoma. J. Clin. Invest. *128*, 294–308. https://doi.org/10.1172/JCI92513.

Watt, M.J., Clark, A.K., Selth, L.A., Haynes, V.R., Lister, N., Rebello, R., Porter, L.H., Niranjan, B., Whitby, S.T., Lo, J., et al. (2019). Suppressing fatty acid uptake has therapeutic effects in preclinical models of prostate cancer. Sci. Transl. Med. *11*, eaau5758. https://doi.org/10.1126/scitranslmed.aau5758.

Wilfling, F., Wang, H., Haas, J.T., Krahmer, N., Gould, T.J., Uchida, A., Cheng, J.X., Graham, M., Christiano, R., Fröhlich, F., et al. (2013). Triacylglycerol synthesis enzymes mediate lipid droplet growth by relocalizing from the ER to lipid droplets. Dev. Cell *24*, 384–399. https://doi.org/10.1016/j.devcel.2013.01.013.

Zhang, M., Di Martino, J.S., Bowman, R.L., Campbell, N.R., Baksh, S.C., Simon-Vermot, T., Kim, I.S., Haldeman, P., Mondal, C., Yong-Gonzales, V., et al. (2018). Adipocyte-derived lipids mediate melanoma progression via FATP proteins. Cancer Discov. *8*, 1006–1025. https://doi.org/10.1158/2159-8290.CD-17-1371.

Zhou, Y., Zhou, B., Pache, L., Chang, M., Khodabakhshi, A.H., Tanaseichuk, O., Benner, C., and Chanda, S.K. (2019). Metascape provides a biologist-oriented resource for the analysis of systems-level datasets. Nat. Commun. *10*, 1523. https://doi.org/10.1038/s41467-019-09234-6.

STAR★METHODS

KEY RESOURCE TABLE

REAGENT or RESOURCE	SOURCE	IDENTIFIER
Antibodies		
Rabbit monoclonal anti-DGAT1	Abcam	RRID: ab181180
Mouse monoclonal anti-Vinculin	Proteintech	RRID: 66305-1-Ig
Rabbit monoclonal anti- GFP (D5.1) XP	Cell Signaling	RRID: Cat# 2956
Rabbit polyclonal anti-Beta Tubulin	Proteintech	RRID: 10094-1-AP
Rabbit polyclonal anti-Phospho-AMPKα (Thr172)	Cell Signaling	RRID: Cat# 50081
Rabbit polyclonal anti-Phospho-Raptor (Ser792)	Cell Signaling	RRID: Cat# 2083
Rabbit polyclonal anti-PINK1	Proteintech	RRID: 23274-1-AP
Rabbit polyclonal anti-Parkin	Proteintech	RRID: 14060-1-AP
Rabbit polyclonal anti-Caspase-3	Cell Signaling	RRID: Cat# 9662
Rabbit polyclonal anti-SOD1	Proteintech	RRID: 10269-1-AP
Rabbit polyclonal anti-SOD2	Proteintech	RRID: 24127-1-AP
Mouse monoclonal anti-GAPDH	Proteintech	RRID: 60004-1-Ig
Rabbit monoclonal anti-Phospho-Akt (Ser473)	Cell Signaling	RRID: Cat# 4060
Mouse monoclonal anti-ERK 1/2	Santa Cruz Biotechnology	RRID: sc-135900
Rabbit monoclonal phospho-ERK 1/2 (Thr202/Tyr204)	Cell Signaling	RRID: Cat# 4370
Rabbit polyclonal anti-4 Hydroxynonenal	Abcam	RRID: ab46545
Rabbit Sestrin 2 Polyclonal antibody	Proteintech	RRID: 10795-1-AP
Mouse monoclonal anti-γ-Tubulin	Sigma-Aldrich	RRID: T5326
Bacterial and virus strains		
NEB® 10-beta Competent E. coli (High Efficiency)	New England Biolabs	Cat. No: C3019H
Chemicals, peptides, and recombinant proteins		
Trypsin porcine pancreas (proteomics grade)	Sigma-Aldrich	T6567
Lysyl Endopeptidase	FUJIFILM Wako Chemicals	129-02541
Pre-cast gradient gel: Nu-PAGE 4–12% Bis-Tris Gel 1.0 mm 10 well	Invitrogen	NP0321BOX
Sep-Pak Classic C18 cartridges	Waters	WAT051910
Solid Phase Extraction Disk “Empore” C18 (Octadecyl) 3M	Agilent Technologies	2215
Solid Phase Extraction Disk “Empore” C8 (Octyl) 3M	Agilent Technologies	2214
L-ARGININE:HCL	Cambridge Isotope Laboratories	CLM-2265-H-0.25
L-ARGININE:HCL	Cambridge Isotope Laboratories	CNLM-539-H-0.5
L-ARGININE:HCL	Sigma-Aldrich	A6969
L-LYSINE:2HCL	Cambridge Isotope Laboratories	DLM-2640-0.5
L-LYSINE:2HCL	Cambridge Isotope Laboratories	CNLM-291-H-0.5
L-LYSINE:2HCL	Sigma-Aldrich	L8662
2,5-Dihydroxybenzoic acid	Sigma-Aldrich	85707
RPMI 1640 Medium for SILAC	ThermoFisher Scientific	88365

(*Continued on next page*)

Continued

REAGENT or RESOURCE	SOURCE	IDENTIFIER
TRIzol™ Reagent	ThermoFisher Scientific	Cat. No. 15596026
T863- DGAT1 Inhibitor	Sigma-Aldrich	SML0539-5MG
A922500- DGAT1 Inhibitor	Stratech Scientific Ltd	A4382-APE-50mg
AZD3988- DGAT1 Inhibitor	Tocris Bioscience	Cat. No. 4837
AZD7687- DGAT1 Inhibitor	Stratech Scientific Ltd	A3215-APE-5mg
PF 4708671	Generon	A11755-25
Polybrene Transfection Reagent	Millipore UK Limited	R-1003-G
PARAQUAT DICHLORIDE X-HYDRATE PESTANAL	Sigma-Aldrich	36541-100MG
Menadione	Fluorochem Limited	049845-25G
Oligomycin A	Sigma-Aldrich	75351
FCCP	Cambridge Bioscience Limited	2398-5
Oligomycin Complex	Stratech Scientific Ltd	C3007-APE-5mg
Rotenone, PESTANAL, analytical standard	Scientific Laboratory Supplies Ltd	45656-250MG
Antimycin A from Streptomyces sp.	Sigma-Aldrich	A8674
PF-06424439- DGAT2 Inhibitor	Sigma-Aldrich	PZ0233
Ebselen	Sigma-Aldrich	E3520-25MG
Tempol	Stratech Scientific Ltd	S2910-SEL-100mg
Etomoxir	Stratech Scientific Ltd	A3404-APE-10mg
Ferrostatin-1	Stratech Scientific Ltd	S7243
ML385	Selleckchem	S8790
ATN-224	Cayman Chemical	23553
Ammonium tetrathiomolybdate	Sigma-Aldrich	323446
BODIPY 581/591 C11 (Lipid Peroxidation Sensor)	ThermoFisher Scientific	D3861
MitoPerOx, fluorescent mitochondria-targeted lipid peroxidation probe	Abcam	ab146820
BODIPY™ 493/503 (4,4-Difluoro-1,3,5,7,8-Pentamethyl-4-Bora-3a,4a-Diaza-s-Indacene)	Life Technologies	D3922
HCS LipidTOX™ Green Neutral Lipid Stain	ThermoFisher Scientific	H34475
Molecular Probes MitoSOX Red Mitochondrial Superoxide Indicator	ThermoFisher Scientific	11579096
DIHYDROETHIDIUM	Cambridge Bioscience	12013-5mg-CAY
H2DCFDA	Tocris Bioscience	5935/100
Hoechst 33342	New England Biolabs	4082S
Lipofectamine RNAiMAX Transfection Reagent	ThermoFisher Scientific	10601435
Lipofectamine Transfection Reagent	Life Technologies	18324020
FuGENE HD Transfection Reagent	Promega UK	E2311
Seahorse XFe96 FluxPak mini	Agilent Technologies	102601-100
Seahorse XF DMEM medium, pH 7.4, 500 mL.	Agilent Technologies	103575-100
qPCRBIO SyGreen Mix Separate-ROX	PCR Biosystems	PB20.14
Crystal violet solution	Sigma-Aldrich	V5265-250ML
Critical commercial assays		
Click-iT EdU Alexa Fluor 488 Imaging Kit-1 kit	Life Technologies	C10337
MitoProbe JC-1 Assay Kit	Life Technologies	M34152
CellEvent Caspase-3/7 Green Detection Reagent	Life Technologies	C10423

(*Continued on next page*)

Continued

REAGENT or RESOURCE	SOURCE	IDENTIFIER
ProtoScript; II First Strand cDNA Synthesis Kit	New England Biolabs	E6560L
RNeasy RNA Extraction Kit	Qiagen	74136
Deposited data		
Proteome data	ProteomeXchange Consortium	PRIDE: PXD017487
Experimental models: Cell lines		
normal human melanocytes	Cascade Biologics	C0245C
888MEL	Gift from Claudia Wellbrock	CVCL 4632
Lenti-X^{TM} 293T Cell Line	Takara bio	632180
888MEL Clone 1–3 DGAT Overexpression	This study	N/A
SKMEL28	ATCC	HTB-72
SKMEL105	Memorial Sloan Kettering Cancer Center	N/A
A375	ATCC	CRL- 1619
MM485	Gift from Claudia Wellbrock	CVCL_2610
WM266-4	Gift from Claudia Wellbrock	CVCL_2765
LOX-IMVI	Sigma-Aldrich	SCC201
SKMEL5	ATCC	HTB-70
Experimental models: Organisms/strains		
tp53m214K/m214k; mitfa−/−	Leonard Zon	N/A
tp53m214K/m214k; mitfa−/−; braf V600E	Craig Ceol	N/A
tp53m214K/m214k; mitfa−/−; nras G12D	This study	N/A
Recombinant DNA		
pMDLg/pRRE	Gift from Angeliki Malliri	Addgene #12251
pRSV-Rev	Gift from Angeliki Malliri	Addgene #12253
pMD2.G	Gift from Angeliki Malliri	Addgene #12259
pCDH-EF1α-MCS*-T2A-GFP	Gift from Andrew Gilmore	Systems Bioscience CD526A-1
pCDH-EF1α-MCS*-T2A-mApple	This study	N/A
pCDH-EF1α-DGAT1-T2A-mApple	This study	N/A
pDONR221	ThermoFisher Scientific	12536017
pDest-mitfa:dgat1a-pA	This study	N/A
pDest-mitfa:dgat2-pA	This study	N/A
pCS2-TP	Gift from Koichi Kawakami	N/A
pRK7-HA-S6K1-WT	Addgene	#8984
pRK7-HA-S6K1- F5A-E389-deltaCT	Addgene	#8990
pcDNA3.1-mMaroon1	Addgene	# 83840
pcDNA3.1-DGAT1	This study	N/A
Software and algorithms		
Fiji- Image J	(Schindelin et al., 2012)	https://imagej.net/Fiji
GraphPad Prism version 8.0.0	GraphPad Software	www.graphpad.com
Enrichr	(Chen et al., 2013)	https://amp.pharm.mssm.edu/Enrichr/
WebGestalt	(Liao et al., 2014)	http://www.webgestalt.org/#
Metascape	(Zhou et al., 2019))	https://metascape.org/gp/index.html#/main/step1
MaxQuant	(Cox et al., 2011)	https://www.maxquant.org
Lipidomics analysis scripts	This study	https://github.com/computational-metabolomics
Proteome analysis scripts	This study	https://github.com/JoWatson2011/DGAT_2019
FlowJo	www.flowjo.com	N/A

RESOURCE AVAILABILITY

Lead contact

Further information and requests for resources and reagents should be directed to and will be fulfilled by the lead contact, Adam Hurlstone (adam.hurlstone@manchester.ac.uk).

Materials availability

- Plasmids and cell lines generated in this study can be requested from the lead contact.

Data and code availability

- The mass spectrometry proteomics data have been deposited with the ProteomeXchange Consortium PRIDE: PXD017487. Extended Lipidomics Data for zebrafish tumors and human melanoma cell lines can be found in Tables S2–S6.
- Code for lipidomics analysis can be found at https://github.com/computational-metabolomics and proteomics analysis at https://github.com/JoWatson2011/DGAT_2019.

EXPERIMENTAL MODEL AND SUBJECT DETAILS

Zebrafish models

Regulated procedures involving zebrafish were ethically approved by The University of Manchester Animal Welfare and Ethical Review Body (AWERB), or by the UMMS Institution Animal Care and Use Committee (A-2016, A-2171), and carried out under a license issued by the appropriate national regulatory authority. Zebrafish were housed at ~28°C under a 14 h light/10 h dark cycle. Transgenic zebrafish expressing *BRAF*^{V600E} or *NRAS*^{G12D} have been previously described (Casar et al., 2018; Ceol et al., 2011) and were crossed onto a *mitfa*$^{w2/w2}$ (*mitfa*$^{-/-}$) background to suppress melanocyte development and further onto a *tp53*$^{M214K/M214K}$ background to promote tumorigenesis. Melanocyte restoration and simultaneous over-expression of Dgat1a, Dgat2 or EGFP was then achieved by injection of embryos with a mitfa-minigene containing plasmid as previously described (Ceol et al., 2011). Briefly, zebrafish *dgat1a* and *dgat2* were amplified from cDNA of wild-type 48 h post-fertilization zebrafish embryos, and subcloned into the pDONR221 vector (see Table S7 for oligonucleotide sequences). The pDest-mitfa:dgat1a-pA and pDest-mitfa:dgat2-pA destination vectors were created using an LR clonase reaction consisting of p5E-mitfap, pME-dgat1a or pME-dgat2, p3E-pA and an empty destination vector. Expression plasmid was injected into zebrafish zygotes along with Tol2 mRNA. pCS2-TP plasmid for Tol2 mRNA generation was a kind gift from Dr Koichi Kawakami (National Institute of Genetics). Sufficient embryos for all experimental arms were generated simultaneously, pooled and then randomly assigned to a construct, although formal randomization techniques were not used. Zebrafish were group-housed according to the construct. Only zebrafish embryos with near complete melanocyte rescue at 5 days were retained for further analysis. Analysis of tumor formation was not performed blinded to the construct identity. Sample sizes were not predetermined based on statistical power calculations but were based on our experience with these assays. To assess the statistical significance of differences in overall survival, we used Mantel–Cox's log-rank tests.

Mouse xenograft assays

Mice were housed in the University of Manchester Biological Safety Unit. CD1® nude mice (female, 8 weeks of age) were injected subcutaneously into the left flank with 4×10^6 A375 cells or MM485 cells (in PBS). When animals had developed melanoma nodules of ~100 mm^3, animals were allocated randomly to a treatment group and drug administration was initiated (4–6 mice per group). Treatment was by oral gavage once daily with vehicle (1% Tween80 in PBS), DGAT1 inhibitor A922500 (90 mg/kg) or SOD1 inhibitor ammonium tetrathiomolybdate (5 mg/kg). After the indicated number of days, tumors were isolated and analyzed as described.

Cell lines

Human melanoma cell lines were cultured in High Glucose DMEM with 10% FBS, and penicillin–streptomycin (Sigma) at 37°C and 5% CO_2. Normal human melanocytes were purchased from Cascade Biologics and cultured according to the manufacturer's guidelines. Lenti-X cells were cultured in High Glucose DMEM with 10 %v/v FBS, and penicillin–streptomycin (Sigma) at 37°C and 5% CO_2. All cells tested negative for mycoplasma and cell lines were authenticated using STR profiling (See Table S8 for DGAT1 characteristics of cell lines used in this study.).

METHOD DETAILS

Compounds and antibodies

Compounds were used at the following concentrations unless otherwise noted: 50 μM AZD3988 (Tocris), 30 μM A922500 (Stratech), 50 μM AZD7687 (Stratech), 70 μM T863 (Sigma), 1 μM Oligomycin (Sigma), 0.5 μM FCCP (Sigma), 1 μM Antimycin-A (Sigma), 1 μM Rotenone (Sigma), 50 μM PF-06424439 (Sigma), 5 μM Ebselen (Tocris), 1 mM Tempol, 200 μM Paraquat (Sigma), Menadione (Sigma), 100 μM Etomoxir (Sigma), 2 μM Ferrostatin-1 (Sigma), 5 μM ML385 (Selleckchem), and 1 μM ATN-224 (Cayman Chemical).

Antibodies against DGAT1 (ab54037) and 4-Hydroxynonenal (ab46545) were purchased from abcam. Antibodies against Vinculin (66305-1-Ig), Beta-Tubulin (10094-1-AP), PINK1 (23274-1-AP), Parkin (14060-1-AP), SOD1 (10269-1-AP), SOD2 (24127-1-AP), SESN2 (10795-1-AP) and GAPDH (60004-1-Ig) were purchased from Proteintech. Antibodies against phospho-AMPK (50081), phospho-RAPTOR (2083), Caspase-3 (9662), phospho-ERK (4370) and GFP (2956) were purchased from Cell Signalling. The antibody against gamma-tubulin (T5326) was purchased from Sigma. The antibody against ERK2 (C-14) was from Santa Cruz Biotechnology.

Plasmid cloning and transfection & siRNA transfection

The following plasmid used was purchased from Addgene: pcDNA3.1-mMaroon1 (83840). The GFP and WPRE elements were excised from pCDH-MCS-T2A-copGFP (a kind gift from Andrew Gilmore, The University of Manchester) using BspEI and KpnI. mApple (BspEI and XhoI adapters) and WPRE (XhoI-KpnI adapters) were PCR amplified, digested and subcloned to create the pCDH-MCS-T2A-mApple vector. DGAT1 was further subcloned into the both the pCDNA3.1 vector and pCDH-MCS-T2A-mApple using the MCS. All plasmids were transfected using Lipofectamine (Invitrogen) following standard protocols. All siRNA was transfected using Lipofectamine RNAi Max (Invitrogen) following standard protocols.

Viral transduction

Briefly, Lenti-X^{TM} HEK293 cells (Takara Bio) were transfected with pMDLg/pRRE, pMD2.G, pRSV-Rev plasmids (all kind gifts from Angeliki Malliri, Cancer Research UK Manchester Institute) and pCDH-EF1α-DGAT1-T2A-mApple viral vectors using Fugene (Promega) following standard protocols. The viral containing supernatant was filtered using a 0.45 μm filter and frozen at -80°C prior to transduction of target cells. The supernatant containing the viral particles was added to target cells along with 10 ng/mL Polybrene (Millipore) for 24 h. Target cells were then grown and selected from single cell colonies.

Protein lysate preparation and western blotting

Cells were washed with PBS and lysed with sample buffer (62.5 mM TRIS pH 6.8, 2 %w/v Sodium dodecyl sulfate (SDS), 10 %v/v glycerol, 0.01%w/v bromophenol blue, 3 %v/v 2-mercaptoethanol). Lysates were then sonicated and heated to 95°C for 10 minutes prior to being evenly loaded onto SDS-polyacrylamide gels using the Mini Trans-Bot electrophoresis system (Biorad), followed by transfer to PVDF using standard western blotting procedures.

Lipid droplet staining and image analysis

BODIPY 493/503

Indicated cells were stained with 2 μM BODIPY 493/503 (ThermoFisher Scientific) and 5 ng/mL Hoecsht 3342 (Cell Signalling) for 30 minutes prior to fixing in 4 %w/v paraformaldehyde and imaging using a Leica microscope system. Images were processing using Fiji.

Lipidtox

Indicated cells were fixed in 4 %w/v paraformaldehyde and stained with LipidTox Green (ThermoFisher Scientific) according to manufacturer's instructions, and 5 ng/mL Hoecsht 3342 (Cell Signalling) for 15 minutes prior to imaging using a Leica microscope system. Images were processing using Fiji.

RNA isolation and real-time PCR analysis

RNA from cell lines was isolated with TRIZOL® (Invitrogen). After chloroform extraction and centrifugation, 5 μg RNA was DNase treated using RNase-Free DNase Set (Qiagen). 1 μg of DNase treated RNA was then taken for cDNA synthesis using the Protoscript I first strand cDNA synthesis kit (New England Biolabs). Selected genes were amplified by quantitative real time PCR (RT-qPCR) using Sygreen (PCR Biosystems). Relative expression was calculated using the delta-delta CT methodology and beta-actin was used as reference housekeeping gene. Sequences for primers used can be found in the Table S7.

Flow cytometry

Mitochondrial membrane potential

Indicated cell lines were trypzinized and pelleted by centrifugation at 500 g for 5 min, washed with PBS. For mitochondrial membrane potential cells were stained with 2 μM JC-1 (Life Technologies) for 30 minutes at 37°C. For positive control samples 0.5μM FCCP was added simultaneously with JC-1. Data was acquired by the BD BIOsciences Foretessa and quantified using the Flowjo software. A minimum of 10,000 cells were analyzed per condition.

Lipid peroxidation

Indicated cell lines were trypzinized and pelleted by centrifugation at 500 g for 5 min, followed by a PBS wash. For lipid peroxidation cells were stained with either 5 μM BODIPY^{TM} 581/591 C11 (ThermoFisher Scientific) or MitoPerOx (Abcam) for 30 minutes at 37°C. Data was acquired by the BD BIOsciences Foretessa and quantified using the Flowjo software. A minimum of 10,000 cells were analyzed per condition.

Mitochondrial ROS

Indicated cell lines were trypzinized and pelleted by centrifugation at 500 g for 5 min, followed by a PBS wash. For mitochondrial specific ROS detection, cells were stained with 2.5 μM Mitosox (ThermoFisher Scientific) for 30 minutes at 37°C. Data was acquired by the BD BIOsciences Foretessa and quantified using the Flowjo software. A minimum of 10,000 cells were analyzed per condition.

Proliferation assays

Crystal violet

Indicated cells were stained and fixed with 0.5 %w/v crystal violet (Sigma) in 4 %w/v paraformaldehyde/PBS for 30 minutes. Fixed cells were then solubilized in 2 %w/v SDS/PBS and absorbance measured at 595 nm using Synergy H1 microplate reader (BioTek).

EdU incorporation

Indicated cells were labelled with 20 μM 5-ethynyl-2′-deoxyuridine (EdU) for 4 h and processed following the manufacturer's protocol (Click-iT® EdU Alexa Fluor® 488 Imaging Kit, Thermo Fisher). Prior to imaging cells were then stained with 5 ng/mL Hoecsht 3342 for 15 minutes. Stained cells were analyzed using a using a Leica microscope system. Images were processing using Fiji.

Incucyte cell-proliferation assay and apoptosis assay

Indicated cell lines were seeded into 24-well plates at a density of 15,000–20,000 cells per well, depending on growth rate and the design of the experiment. After 24 h drugs or siRNA were added, and cells were imaged every hour using the Incucyte ZOOM (Essen Bioscience) Phase-contrast images were analyzed to detect cell proliferation based on cell confluence. For cell apoptosis, caspase-3 and caspase-7 green apoptosis-assay reagent (Life Technologies) was added to the culture medium following manufacturer's instructions. Cell apoptosis was analyzed based on green fluorescent staining of apoptotic cells.

Dihydroethidium assay

Cells were stained with 5 μM Dihydroethidium for 20 minutes in the dark at 37°C. Fluorescence was measured at excitation 480 nm emission 570 nm using Synergy H1 micro plate reader (BioTek). Fluorescence values were normalized to cell number by staining the cells with crystal violet after fluorescence read.

Cancer bioinformatics

We evaluated both point mutations and CNV in the TCGA SKCM firehose legacy, TCGA pan-cancer and Cancer Cell Line Encyclopedia datasets using the cBioPortal platform (Gao et al., 2013). The GISTIC2.0 algorithm was used to identify focal amplifications (Mermel et al., 2011). Gene Ontology analysis was carried out using both enrichR (Chen et al., 2013) and metascape software (Zhou et al., 2019). Association between mRNA expression in TCGA datasets and survival was evaluated using OncoLnc (Anaya, 2016). mRNA levels determined by microarray were accessed through the Oncomine platform (Rhodes et al., 2004).

QUANTIFICATION AND STATISTICAL ANALYSIS

Data was tested for normality using the Shapiro-Wilk test. Data was considered to be normally distributed if $p > 0.05$. Differences in the number of lipid droplets per cell, relative cell number and percentage EdU incorporation between DMSO and drug treated cells were assessed using an unpaired two-sided *t*-test, or Mann-Whitney test if data were not normally distributed. In comparing the differences in these same characteristics between cells transfected with either non-target or one of several siRNA oligonucleotides, a one-way ANOVA with Tukey's multiple comparisons test (or Friedman with Dunn's multiple comparisons test if data were not normally distributed) was used to measure significance. Differences were considered significant if $p < 0.05$. All data obtained were analyzed using Graphpad Prism 8.1.

Proteomics

SILAC labelling

For quantitative mass spectrometry, A375 cells were labelled in SILAC DMEM supplemented with 10 %v/v dialyzed fetal bovine serum (Sigma), 2 mM glutamine, 100 U/mL penicillin and 100 μg/mL streptomycin for 15 days to ensure complete incorporation of amino acids. Two cell populations were obtained: one labelled with natural variants of the amino acids (light label; Lys0, Arg0) and a second one with heavy variants of the amino acids (L-[13C6,15N4]Arg (+10) and L- [13C6,15N2]Lys (+8)) (Lys8,Arg10). The light amino acids were from Sigma, while their heavy variants were from Cambridge Isotope Labs.

Sample preparation for mass spectrometry analysis

Cells from the two SILAC conditions treated as indicated were lysed at 4°C in ice cold modified RIPA buffer (50 mM Tris, pH 7.5, 150 mM NaCl, 1 %v/v NP-40, 0.1 %w/v sodium deoxycholate, 1 mM EDTA, 5 mM β-glycerolphosphate, 5 mM sodium fluoride, 1 mM sodium orthovanadate, 1 complete inhibitor cocktail Tablet per 50 mL). Proteins were precipitated for two hours at −20°C in four-fold excess of ice-cold acetone. The acetone-precipitated proteins were solubilized in denaturation buffer (10 mM HEPES, pH 8.0, 6 M urea, 2 M thiourea) and the SILAC-labelled lysates were mixed 1:1 based on protein concentrations. Proteins were reduced with 1 mM dithiothreitol (DTT) for 60 min, alkylated with 5.5 mM chloroacetamide (CAA) for 60 min and digested first with endoproteinase Lys-C (Wako, Osaka, Japan) and then, after a five-fold dilution with 50 mM ammonium bicarbonate (ABC), with trypsin (modified sequencing grade, Sigma). The peptide mixture was desalted and concentrated on a C18-SepPak cartridge (Waters, USA) and eluted with 50 %v/v acetonitrile. Equal amounts of SILAC lysates were then mixed 1:1, reduced with DTT, and alkylated with CAA before being resolved on SDS-PAGE (8–12 %w/v, Invitrogen). Separated proteins were fixed in the gel and visualized with colloidal Coomassie staining (Invitrogen). Each gel lane was excised and separated into eight segments that were sliced, destained with 50 %v/v EtOH in 25 mM ABC and dehydrated with 100 %v/v EtOH. Proteins were digested with sequence-grade trypsin (Sigma) overnight. Trypsin activity was quenched by acidification with trifluoroacetic acid (TFA) and peptides were extracted

from the gel sections with increasing concentrations of acetonitrile. Organic solvent was evaporated in a vacuum centrifuge, as described (Francavilla et al., 2016).

Mass spectrometry analysis

Enriched in-gel digested peptides were desalted and concentrated on STAGE-tips with two C18 filters and eluted using 40 %v/v acetonitrile, dried and reconstituted in 5 %v/v acetonitrile in 0.1 %v/v formic acid prior to analysis by LC-MS/MS using an UltiMate 3000 Rapid Separation LC (RSLC, Dionex Corporation, Sunnyvale, CA) coupled to a QE HF (Thermo Fisher Scientific, Waltham, MA) mass spectrometer. Mobile phase A was 0.1 %v/v formic acid in water and mobile phase B was 0.1 %v/v formic acid in acetonitrile and the analytical column utilized was a 75 mm × 250 μm inner diameter 1.7 μm CSH C18 (Waters). Samples were transferred to a 5 μL loop before loading on to the column at a flow of 300 nL/min for 5 minutes at 5 %v/v B. The loop was subsequently taken out of line and the peptides separated using a gradient that went from 5 %v/v to 7 %v/v B and from 300 nL/min to 200 nL/min in 1 min followed by a shallow gradient from 7 %v/v to 18 %v/v B in 64 min, then from 18 %v/v to 27 %v/v B in 8 min and finally from 27 %v/v B to 60 %v/v B in 1 min. The column was washed at 60 %v/v B for 3 min before re-equilibration to 5 %v/v B in 1 min. At 85 min, the flow was increased to 300 nL/min until the end of the run at 90 min. Mass spectrometry data was acquired in a data dependent manner for 90 min in positive mode. Peptides were selected for fragmentation automatically by data dependent analysis on a basis of the top 8 (phospho-proteome) or top 12 (proteome) peptides with m/z between 300 to 1750 Th and a charge state of 2, 3 or 4 with a dynamic exclusion set at 15 sec. The MS Resolution was set at 120,000 with an AGC target of 3e6 and a maximum fill time set at 20 ms. The MS2 Resolution was set to 30,000, with an AGC target of 2e5, a maximum fill time of 45 ms, isolation window of 1.3 Th and a collision energy of 28.

Data analysis of quantitative MS data

Raw data were analyzed with the MaxQuant software suite, version 1.5.6.5, with the integrated Andromeda search engine (Cox et al., 2011). Proteins were identified by searching the HCD-MS/MS peak lists against a target/decoy version of the human Uniprot database, which consisted of the complete proteome sets and isoforms (2016 release) supplemented with commonly observed contaminants such as porcine trypsin and bovine serum proteins. Tandem mass spectra were initially matched with a mass tolerance of 7 ppm on precursor masses and 0.02 Da or 20 ppm for fragment ions. Cysteine carbamidomethylation was searched as a fixed modification. Protein N-acetylation, oxidized methionine and either deamidation of asparagine and glutamine (proteome analysis) were searched as variable modifications. Labelled lysine and arginine were specified as fixed or variable modification, depending on prior knowledge about the parent ion (MaxQuant SILAC identification). False discovery rate was set to 0.01 for peptides, proteins and modification sites. Minimal peptide length was six amino acids. Only peptides with Andromeda score >40 were included. Potential contaminants, reverse sequenced peptides and phosphorylation sites with a localization probability of less than 0.75 (class I) (Olsen et al., 2006) were filtered from the dataset. The remaining data were filtered to remove sites or peptides without quantification in at least two of the three replicates for each time point. The median of the replicates was taken. Sites or peptides with a SILAC ratio of greater than 1.5 were considered upregulated whilst those with a ratio less than 0.75 were considered down-regulated. Gene network visualization was performed using enrichR (Chen et al., 2013). For the proteome analysis a minimum of three to seven peptide identifications with at least two being uniquely assigned to the particular protein were required. Sequence coverage of the identified proteins was at least 5%. Gene Ontology analysis was carried out using enrichR (Chen et al., 2013) and metascape (Zhou et al., 2019).

Lipidomics

UHPLC-MS

The samples were maintained at 4°C and analyzed applying two Ultra-High-Performance Liquid Chromatography-Mass Spectrometry (UHPLC-MS) methods using a Dionex UltiMate 3000 Rapid Separation LC system (Thermo Fisher Scientific, MA, USA) coupled with a heated electrospray Q Exactive Focus mass spectrometer (Thermo Fisher Scientific, MA, USA). Lipid extracts were analyzed on a Hypersil GOLD column (100 × 2.1 mm, 1.9 μm; Thermo Fisher Scientific, MA, USA). Mobile phase A consisted of 10 mM ammonium formate and 0.1 %v/v formic acid in 60 %v/v acetonitrile/water and mobile phase B consisted of 10 mM ammonium formate and 0.1 %v/v formic acid in 90 %v/v propan-2-ol/water. Flow rate was set for 0.40 mL.min-1 with the following gradient: t = 0.0, 20 %v/v B; t = 0.5, 20 %v/v B, t = 8.5, 100 %v/v B; t = 9.5, 100 %v/v B; t = 11.5, 20 %v/v B; t = 14.0, 20 %v/v B, all changes were linear with curve = 5. The column temperature was set to 55°C and the injection volume was 2 μL. Data were acquired in positive and negative ionization mode separately within the mass range of 150–2000 m/z at resolution 70,000 (FWHM at m/z 200). Ion source parameters were set as follows: Sheath gas = 48 arbitrary units, Aux gas = 15 arbitrary units, Sweep gas = 0 arbitrary units, Spray Voltage = 3.2 kV (positive ion)/2.7 kV (negative ion), Capillary temp. = 380°C, Aux gas heater temp. = 450°C. Data dependent MS2 in 'Discovery mode' was used for the MS/MS spectra acquisition using following settings: resolution = 17,500 (FWHM at m/z 200); Isolation width = 3.0 m/z; stepped collision energies (stepped CE) = 20, 40, 100 [positive ion mode]/40, 60, 130 [negative ion mode]. Spectra were acquired in five different mass ranges: 150–510 m/z; 500–710 m/z; 700–860 m/z; 850–1010 m/z; 1000–2000 m/z. A Thermo ExactiveTune 2.8 SP1 build 2806 was used as instrument control software in both cases and data were acquired in profile mode. Quality control (QC) samples were analyzed as the first ten injections and then every seventh injection with two QC samples at the end of the analytical batch. Two blank samples were analyzed, the first as the sixth injection and then the second at the end of each batch.

Mass spectrometry raw metabolomics data processing

Raw data acquired in each analytical batch were converted from the instrument-specific format to the mzML file format applying the open access ProteoWizard (version 3.0.11417) msconvert tool (Chambers et al., 2012). During this procedure, peak picking and

centroiding, were achieved using vendor algorithms. Isotopologue Parameter Optimization (IPO - version 1.0.0, using XCMS - version 1.46.0) (Libiseller et al., 2015) was used to perform automatic optimization of XCMS (Smith et al., 2006) peak picking parameters. For centWave peak picking algorithm following parameters and ranges were used: min_peakwidth (from 2 to 10); max_peakwidth (from 20 to 60); ppm (from 5 to 15); mzdiff (−0.001 to 0.01); snthresh (10); noise (10,000); prefilter (3); value_of_prefilter (100); mzCenterFun (wMean); integrate (1); fitgauss (FALSE); verbose.columns (FALSE). Optimised XCMS parameters for raw data files deconvolution were: min_peakwidth (6); max_peakwidth (30); ppm (14); mzdiff (0.001); snthresh (10); noise (100); prefilter (3); value_of_prefilter (100); mzCenterFun (wMean); integrate (1); fitgauss (FALSE); verbose.columns (FALSE). For feature grouping method *density* was used with following: minfrac (0.5); minsamp (1); bw (0.25); mzwid (0.01); max (50); sleep (0). A data matrix of metabolite features (*m/z*-retention time pairs) versus samples was constructed with peak areas provided where the metabolite feature was detected for each sample.

Peak matrix processing

The data for pooled QC samples were applied to perform QC filtering. The first five QCs for each batch were used to equilibrate the analytical system and therefore subsequently removed from the data before the data was processed and analyzed. The data from the pooled QC samples were used to apply QC filtering. For each metabolite feature detected QC samples 1–8 were removed (i.e. leaving a blank and 2 QCs at the start of each batch) and the relative standard deviation and percentage detection rate were calculated using the remaining QC samples. Blank samples at the start and end of a run were used to remove features from non-biological origins. Any feature with an average QC intensity less than 20 times the average intensity of the blanks was removed. Any samples with >50% missing values were excluded from further analysis. Metabolite features with a RSD > 30% and present in less than 90% of the QC samples were deleted from the dataset. Features with a <50% detection rate over all samples were also removed. Prior to statistical analysis, the data was normalized using probabilistic quotient normalization (PQN) (Dieterle et al., 2006). For multivariate analysis missing values were replaced by applying *k* nearest neighbor (kNN) missing value imputation (k = 5) followed by log transformation (Parsons et al., 2007).

Lipid annotation

LipidSearch (version 4.2, Thermo Fisher Scientific) was used to annotate peaks based on their MS/MS fragmentation patterns. For lipid annotation, all experimental LC-MS/MS spectra data were searched against a MS/MS lipid library in the LipidSearch software database using the following potential ion forms: positive ion = $[M+H]^+$, $[M+NH_4]^+$, $[M+Na]^+$, $[M+K]^+$, $[M+2H]^{2+}$; negative ion = $[M-H]^-$, $[M+HCOO]^-$, $[M+CH_3COO]^-$, $[M+Cl]^-$, $[M-2H]^{2-}$. The quality of the annotation was graded as A-D. This is defined as: Grade A = all fatty acyl chains and class were completely identified; Grade B = some fatty acyl chains and the class were identified; Grade C = either the lipid class or some fatty acyls were identified; Grade D = identification of less specific fragment ions. Only peaks with an MS/MS identification were discussed in this manuscript. All lipid annotations are reported at a confidence of level 3 according to the Metabolomics Standards Initiative (Sumner et al., 2007).

Univariate statistics

For univariate statistics the normalized data was used to avoid including imputed values in the calculations. Fold changes were computed between all pairs of groups. For 2-group comparisons a t-test was applied to determine features showing a significant different between groups. For comparisons exploring two factors a 2-way ANOVA with an interaction term was applied to determine features showing a significant difference between factor levels. For features found to be significant, Tukey's Honest Significant Difference (HSD) was applied to determine between which levels the difference was significant ($p < 0.05$). A False Discovery Rate (FDR) correction (Benjamini-Hochburg) was applied to adjust for multiple testing and control the number of false positives ($q < 0.05$) for both t-test and ANOVA.

Software

All peak matrix processing, univariate and multivariate analyses were performed in the R environment using STRUCT (STatistics in R Using Class Templates) and STRUCTToolbox packages, which make use of PMP and SBCMS packages. These packages are maintained by Phenome Centre Birmingham and available on GitHub (https://github.com/computational-metabolomics).

Cell Reports, Volume *39*

Supplemental information

Oxidative stress from DGAT1 oncoprotein inhibition in melanoma suppresses tumor growth when ROS defenses are also breached

Daniel J. Wilcock, Andrew P. Badrock, Chun W. Wong, Rhys Owen, Melissa Guerin, Andrew D. Southam, Hannah Johnston, Brian A. Telfer, Paul Fullwood, Joanne Watson, Harriet Ferguson, Jennifer Ferguson, Gavin R. Lloyd, Andris Jankevics, Warwick B. Dunn, Claudia Wellbrock, Paul Lorigan, Craig Ceol, Chiara Francavilla, Michael P. Smith, and Adam F.L. Hurlstone

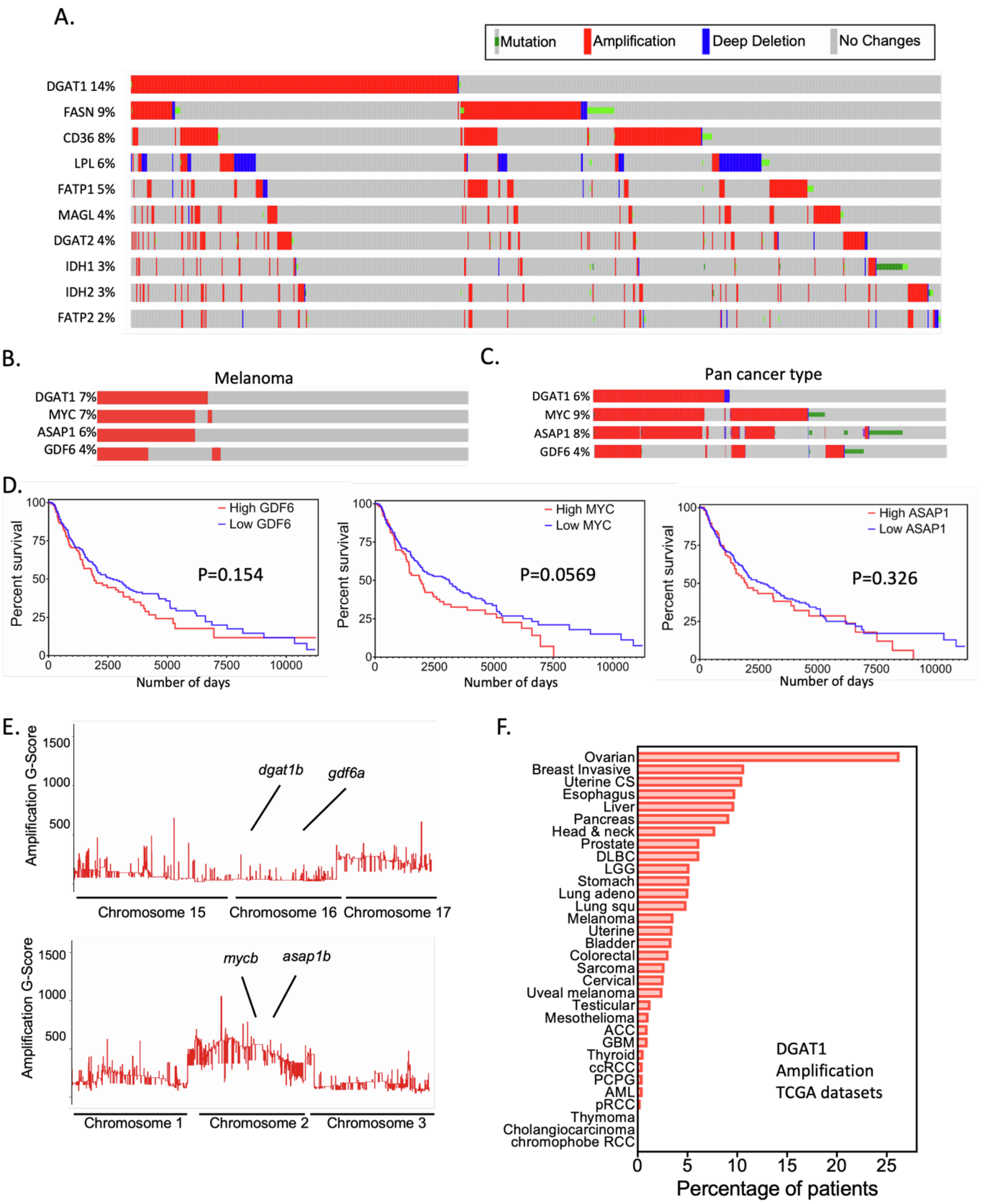

A.
Mutation
Amplification
Deep Deletion
No Changes
DGAT1 14%
FASN 9%
CD36 8%
LPL 6%
FATP1 5%
MAGL 4%
DGAT2 4%
IDH1 3%
IDH2 3%
FATP2 2%
B.
Melanoma
DGAT1 7%
MYC 7%
ASAP1 6%
GDF6 4%
C.
Pan cancer type
DGAT1 6%
MYC 9%
ASAP1 8%
GDF6 4%
D.
High GDF6
Low GDF6
P=0.154
High MYC
Low MYC
P=0.0569
High ASAP1
Low ASAP1
P=0.326
Percent survival
Number of days
E.
Amplification G-Score
dgat1b
gdf6a
Chromosome 15
Chromosome 16
Chromosome 17
mycb
asap1b
Chromosome 1
Chromosome 2
Chromosome 3
F.
Ovarian
Breast Invasive
Uterine CS
Esophagus
Liver
Pancreas
Head & neck
Prostate
DLBC
LGG
Stomach
Lung adeno
Lung squ
Melanoma
Uterine
Bladder
Colorectal
Sarcoma
Cervical
Uveal melanoma
Testicular
Mesothelioma
ACC
GBM
Thyroid
ccRCC
PCPG
AML
pRCC
Thymoma
Cholangiocarcinoma
chromophobe RCC
DGAT1
Amplification
TCGA datasets
Percentage of patients

Figure S1. *DGAT1* amplification and up-regulation associates with poor prognosis in melanoma (related to Figure 1)

(A) Alterations in indicated genes in the TGCA pan-cancer data set accessed via cBioPortal.

(B) Alterations in indicated genes in the TCGA firehose legacy melanoma data set accessed via cBioPortal.

(C) TGCA pan-cancer data set accessed via cBioPortal quantifying alterations in indicated genes.

(D) Kaplan-Meier survival plots for co-amplified genes found on chromosome 8 in melanoma (top 25% *vs* bottom 75%, TCGA database).

(E) G-Score of amplified regions of zebrafish chromosomes found in $BRAF^{V600E}$-positive; *tp53* mutant tumors, locations of human chromosome 8 zebrafish paralogues identified.

(F) TGCA pan-cancer data set accessed via cBioPortal quantifying amplification of *DGAT1* per cancer type.

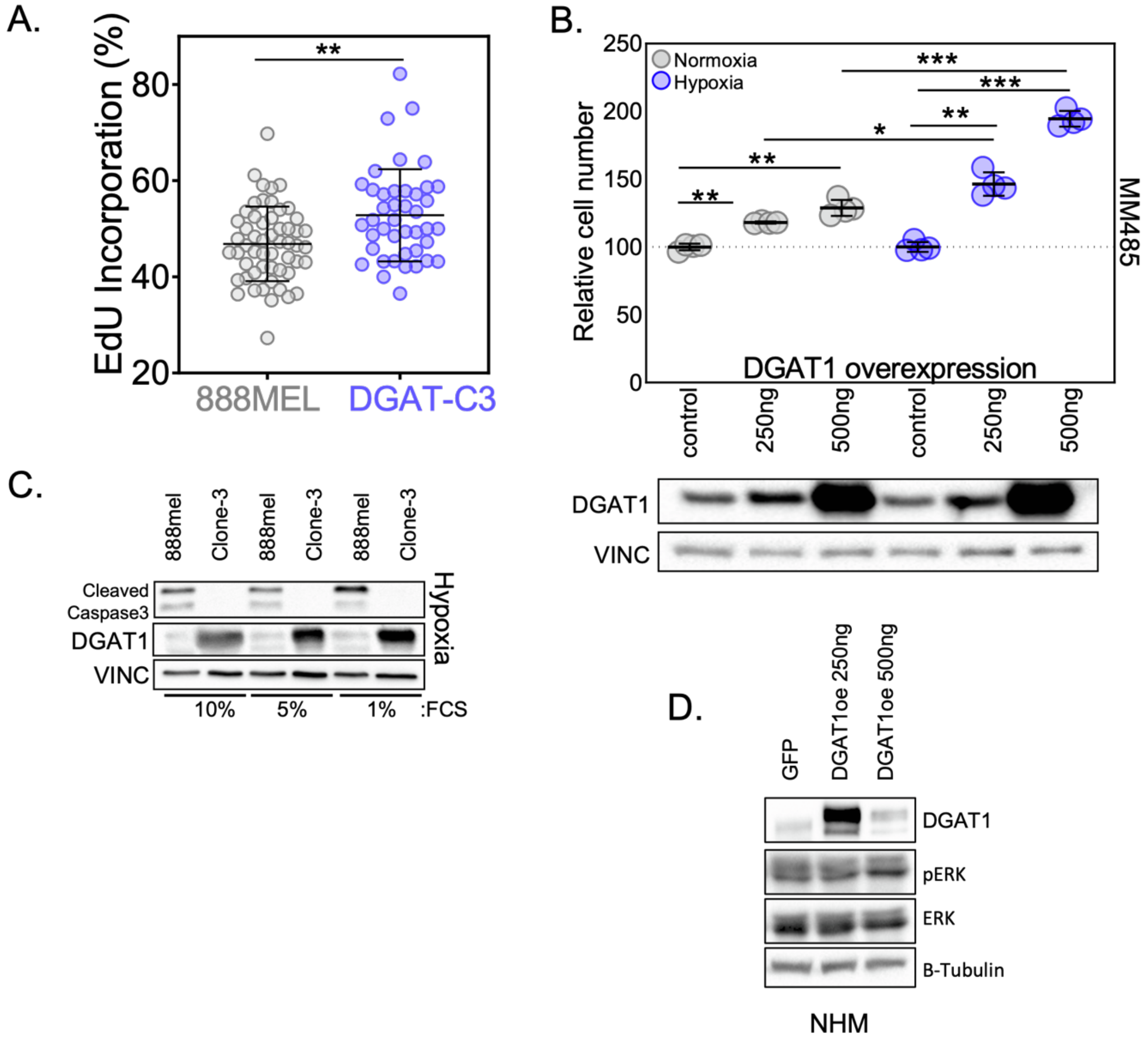

Figure S2. DGAT1 functions as an oncoprotein in zebrafish and human melanocytes (related to Figure 2)

(A) Quantification of the percentage of 888MEL parental cells or Clone 3 DGAT1 over-expressing cells in S-phase following EdU incorporation (Mean ± SD, n>40).

(B) Relative cell number determined by crystal violet following transfection with DGAT1 overexpression vector or an empty vector control under normoxic or hypoxic conditions for 48 h. (1 % O_2) (upper) (Mean, n>3). Corresponding protein expression of DGAT1 (lower).

(C) Protein expression in 888mel parental or Clone 3 DGAT1 overexpressing cells of DGAT1 and cleaved caspase 3 in indicated FCS serum levels under hypoxic (1% O2) conditions.

(D) Protein expression of DGAT1, phospho-ERK and ERK in NHM following overexpression of DGAT1 or GFP.

(A and B) For significance: $*p < 0.05$, $**p < 0.01$, and $***p < 0.001$

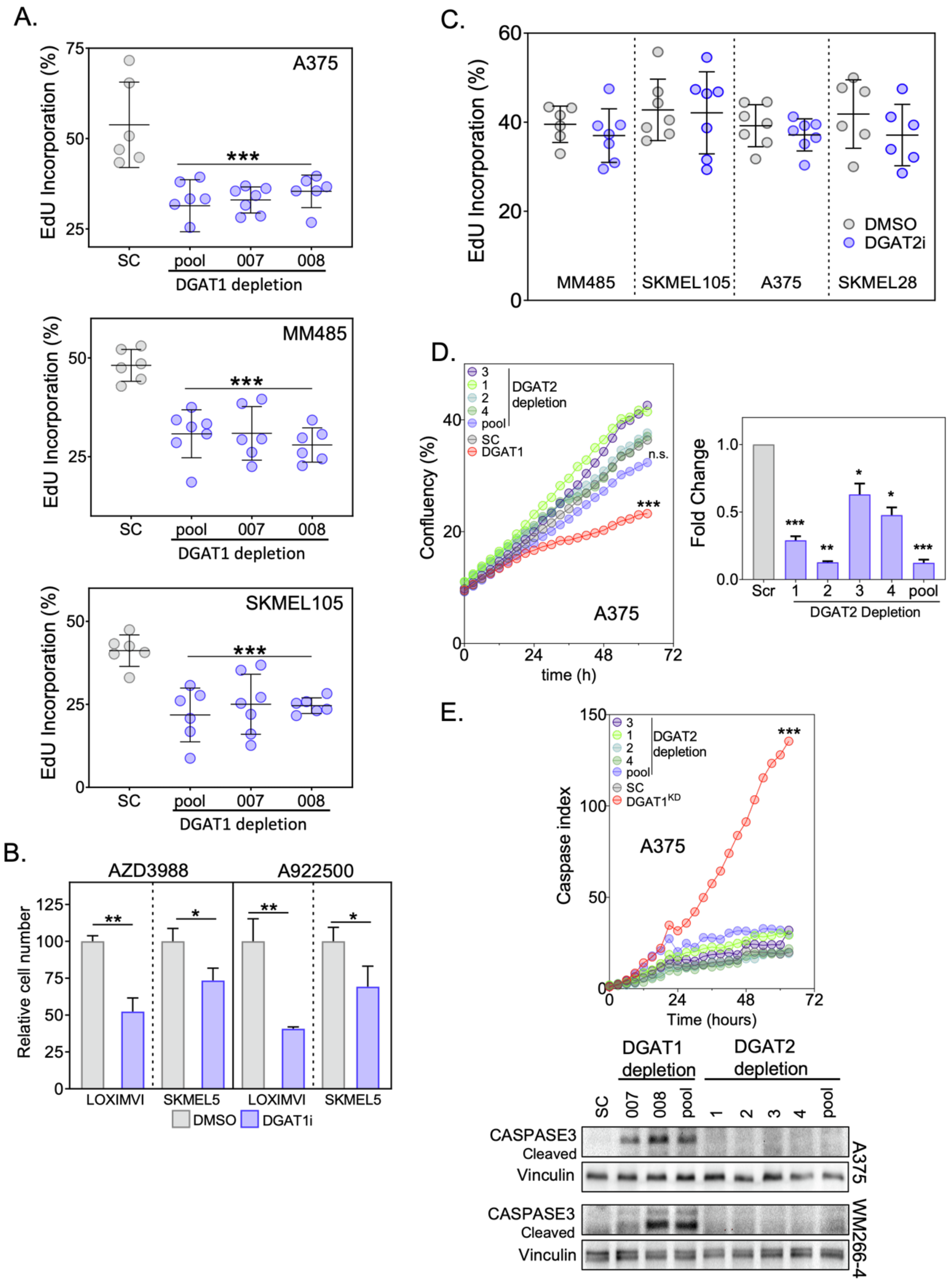
A.
A375
EdU Incorporation (%)
75
50
25
***
SC
pool
007
008
DGAT1 depletion
MM485
EdU Incorporation (%)
50
25
***
SC
pool
007
008
DGAT1 depletion
SKMEL105
EdU Incorporation (%)
50
25
0
***
SC
pool
007
008
DGAT1 depletion
B.
AZD3988
A922500
Relative cell number
125
100
75
50
25
0
**
*
**
*
LOXIMVI
SKMEL5
LOXIMVI
SKMEL5
DMSO
DGAT1i
C.
EdU Incorporation (%)
60
40
20
0
DMSO
DGAT2i
MM485
SKMEL105
A375
SKMEL28
D.
3
1
2
4
pool
DGAT2 depletion
SC
DGAT1
Confluency (%)
40
20
0
n.s.
***
A375
0
24
48
72
time (h)
Fold Change
1.0
0.5
0.0
***
**
*
*
***
Scr
1
2
3
4
pool
DGAT2 Depletion
E.
150
100
50
0
3
1
2
4
pool
DGAT2 depletion
SC
DGAT1^KD
***
Caspase index
A375
0
24
48
72
Time (hours)
DGAT1 depletion
DGAT2 depletion
SC
007
008
pool
1
2
3
4
pool
CASPASE3 Cleaved
Vinculin
A375
CASPASE3 Cleaved
Vinculin
WM266-4

Figure S3. DGAT1 antagonism decreases melanoma cell proliferation and survival (related to Figure 3)

(A) Quantification of the population of cells in S-phase using EdU incorporation following transfection with DGAT1 targeting siRNA (007, 008, pool) or scrambled control for 48 h (Mean ± SD, n>5).

(B) Relative cell number determined by crystal violet staining following 72 h treatment with/without AZD3988 or A922500 (Mean ± SD, n>3).

(C) Quantification of the percentage of cells in S-phase using EdU incorporation following 24 h treatment with/without DGAT2 inhibitor (Mean ± SD, n>5).

(D) Confluency curves determined by time-lapse microscopy in A375 cells following transfection with DGAT2 (001, 002, 003, 004, pool) or DGAT1 targeting scrambled siRNA using an Incucyte zoom system (Mean, n>3) (left). RT-qPCR analysis of DGAT2 gene expression in A375 cells following transfection with DGAT2 targeting siRNA (001, 002, 003, 004, pool) or scrambled control for 48 h (Mean ± SD, n=3) (right).

(E) Cleaved caspase index in A375 cells following transfection with either a DGAT2 targeting (001, 002, 003, 004, pool), DGAT1 targeting or a scrambled siRNA determined by time-lapse microscopy using an Incucyte zoom system (Mean, n=3) (upper). Corresponding protein expression of cleaved caspase3 following transfection with either a DGAT2 targeting siRNA (001, 002, 003, 004, pool), DGAT1 targeting siRNA (007, 008, pool) or a scrambled control (lower).

For significance: $*p < 0.05$, $**p < 0.01$, and $***p < 0.001$

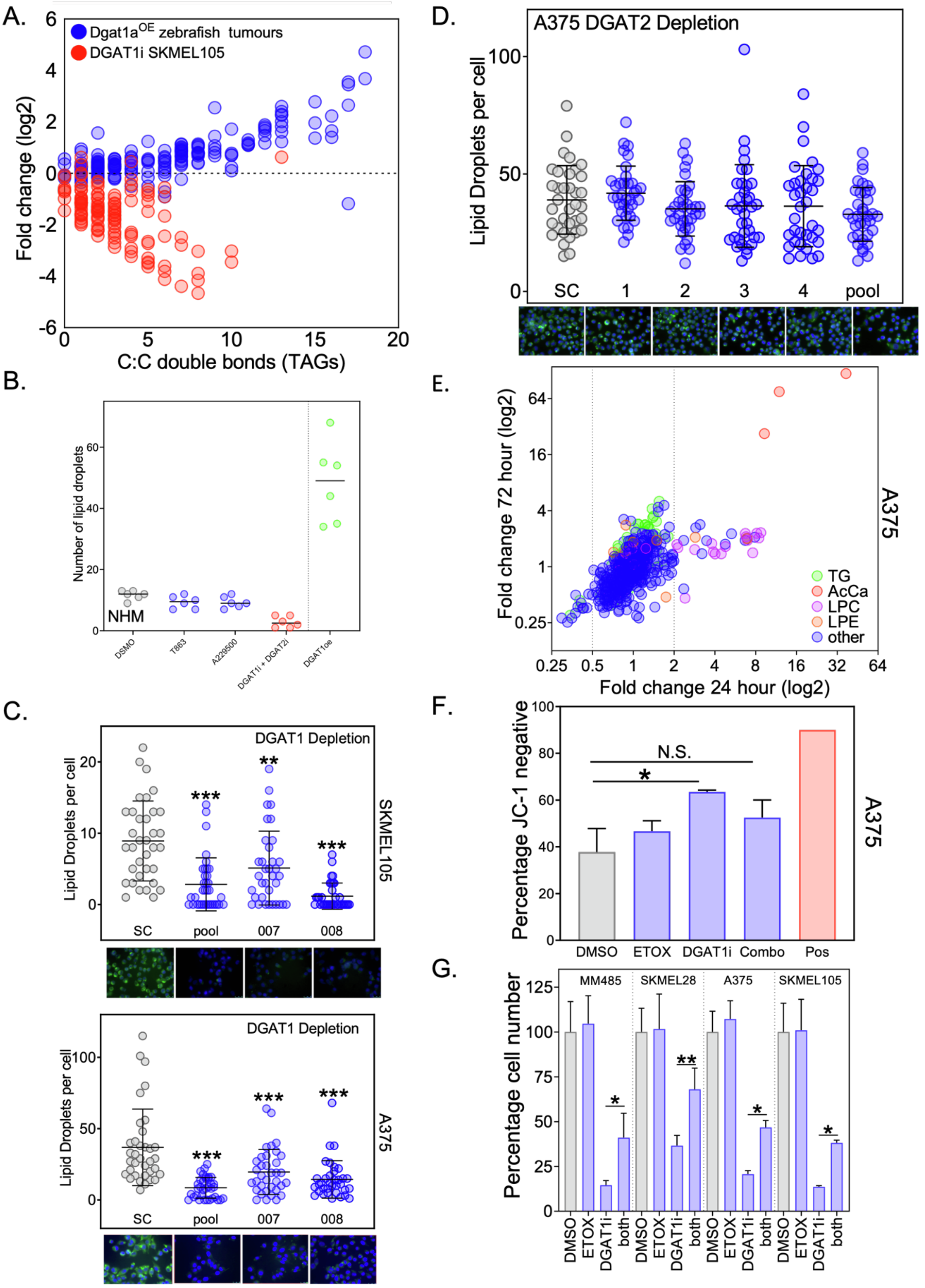

A.
Dgat1a^OE zebrafish tumours
DGAT1i SKMEL105
Fold change (log2)
C:C double bonds (TAGs)
B.
Number of lipid droplets
NHM
DSMO
T863
A229500
DGAT1i + DGAT2i
DGAT1oe
C.
DGAT1 Depletion
Lipid Droplets per cell
SC
pool
007
008
SKMEL105
DGAT1 Depletion
Lipid Droplets per cell
SC
pool
007
008
A375
D.
A375 DGAT2 Depletion
Lipid Droplets per cell
SC
1
2
3
4
pool
E.
Fold change 72 hour (log2)
Fold change 24 hour (log2)
TG
AcCa
LPC
LPE
other
A375
F.
Percentage JC-1 negative
N.S.
DMSO
ETOX
DGAT1i
Combo
Pos
A375
G.
Percentage cell number
MM485
SKMEL28
A375
SKMEL105
DMSO
ETOX
DGAT1i
both

Figure S4. DGAT1-formed lipid droplets act as caretakers of mitochondrial health (related to Figure 4)

(A) UHPLC- lipidomic analysis of NRASG12D-positive Dgat1a-over-expressing (n=6) tumors and SKMEL105 cells following treatment with A922500 for 72 h and showing the number of carbon-carbon double bonds in TAG species compared to the fold increase relative to control samples.

(B) Number of lipid droplets per cell in NHM treated with DGAT1 inhibitors alone or combined with DGAT2 inhibitor or in NHM overexpressing DGAT1 quantified using BODIPY staining (Mean ± SD, n = 6 replicates).

(C) Representative images and quantification of the number of lipid droplets per cell following BODIPY staining. Indicated cell lines were transfected with either DGAT1 targeting siRNA (007, 008, pool) or scrambled control for 48 h (Mean ± SD, n>30).

(D) Representative images and quantification of the number of lipid droplets per cell following BODIPY staining. A375 cells were transfected with either DGAT2 targeting siRNA (001, 002, 003, 004, pool) or a scrambled control for 48 h (Mean ± SD, n>30).

(E) UHPLC- lipidomic analysis of A375 cells following treatment with/without A922500 for 24 or 72 h (showing lipid species that were annotated using MS/MS). Fold-change calculated relative to DMSO treated control (All conditions n=3).

(F) A375 cells were stained with JC-1 dye following treatment with/without A922500 for 48 h and with/without Etomoxir for 4 h. The percentage of cells that lost red J-aggregates was calculated by using 1 μM CCP as a positive control for loss of mitochondrial membrane potential and comparing this to untreated cells to create two populations of cells in the flow cytometry analysis (Mean± SD, n>3).

(G) Relative cell number as determined by crystal violet staining, indicted cell 24 lines were treated with/without A922500 for 48 h and with/without Etomoxir for 24 h (Mean± SD, n=3).

(C, F and G) For significance: $*p < 0.05$, $**p < 0.01$, and $***p < 0.001$

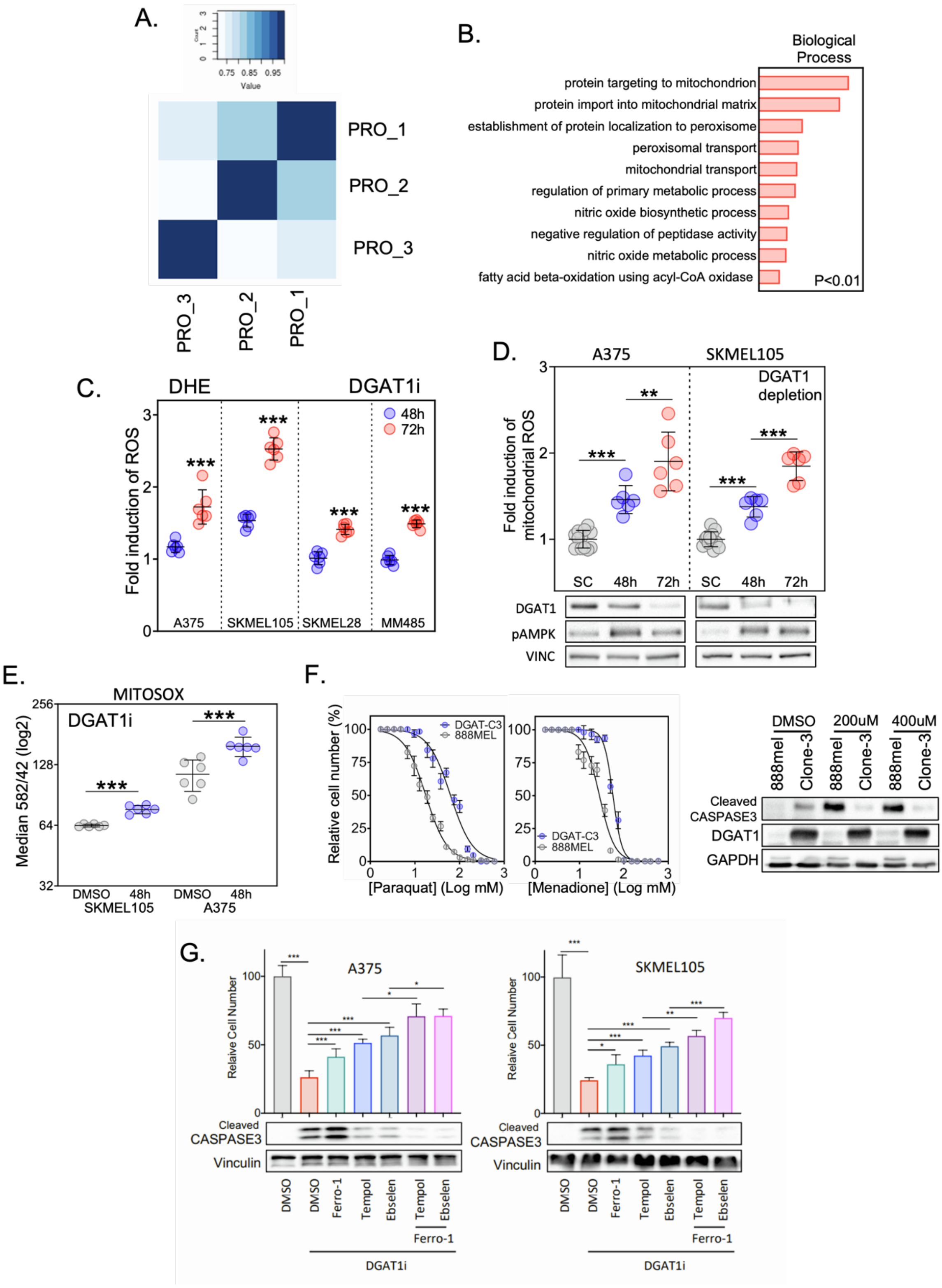
A.
PRO_1
PRO_2
PRO_3
PRO_3
PRO_2
PRO_1
Value
B.
Biological Process
protein targeting to mitochondrion
protein import into mitochondrial matrix
establishment of protein localization to peroxisome
peroxisomal transport
mitochondrial transport
regulation of primary metabolic process
nitric oxide biosynthetic process
negative regulation of peptidase activity
nitric oxide metabolic process
fatty acid beta-oxidation using acyl-CoA oxidase
P<0.01
C.
DHE
DGAT1i
Fold induction of ROS
48h
72h
A375
SKMEL105
SKMEL28
MM485
D.
A375
SKMEL105
DGAT1 depletion
Fold induction of mitochondrial ROS
SC
48h
72h
DGAT1
pAMPK
VINC
E.
MITOSOX
DGAT1i
Median 582/42 (log2)
DMSO
48h
SKMEL105
A375
F.
Relative cell number (%)
DGAT-C3
888MEL
[Paraquat] (Log mM)
[Menadione] (Log mM)
DMSO
200uM
400uM
888mel
Clone-3
Cleaved CASPASE3
DGAT1
GAPDH
G.
A375
SKMEL105
Relaive Cell Number
Relative Cell Number
Cleaved CASPASE3
Vinculin
DMSO
Ferro-1
Tempol
Ebselen
DGAT1i

Figure S5. DGAT1 promotes survival of melanoma cells through suppressing ROS generation (related to Figure 5)

(A) Pearson correlation between total proteome samples following treatment of A375 cells with/without A922500 for 72 h (n=3).

(B) Gene Ontology analysis of up-regulated genes (114) in A375 cells following treatment with A922500 for 72 h compared to DMSO treated control. Gene Ontology analysis was carried out using biological processes (enrichR) ranked by combined score.

(C) ROS levels quantified using dihydroethidium (DHE) fluorescence following treatment with/without A922500 for 48-72 h. Fold change calculated relative to DMSO treated control (Mean, n>4).

(D) ROS levels quantified using mitosox fluorescence following transfection with either DGAT1 targeting siRNA or a scrambled control for 48-72 h. Fold-change calculated relative to scrambled control (Mean ± SD, n=3) (upper). Corresponding protein expression of DGAT1 and phospho-AMPK (lower).

(E) ROS levels quantified using mitosox fluorescence following treatment with A922500 for 48 h (Mean ± SD, n=6).

(F) Drug-dose response curve after 72 h treatment with ROS inducers (left) (Mean ± SD, n=3). Protein expression of cleaved-caspase3 and DGAT1 following 72 h paraquat treatment (right).

(G) Relative cell number following crystal violet staining, indicated cells were either with/without A922500, Ferrsostatin-1 (Ferro-1), Ebselen and Tempol (Mean ± SD, n=3) (Upper). Corresponding protein expression of cleaved caspase-3 in indicated cell lines.

For significance: $*p < 0.05$, $**p < 0.01$, and $***p < 0.001$

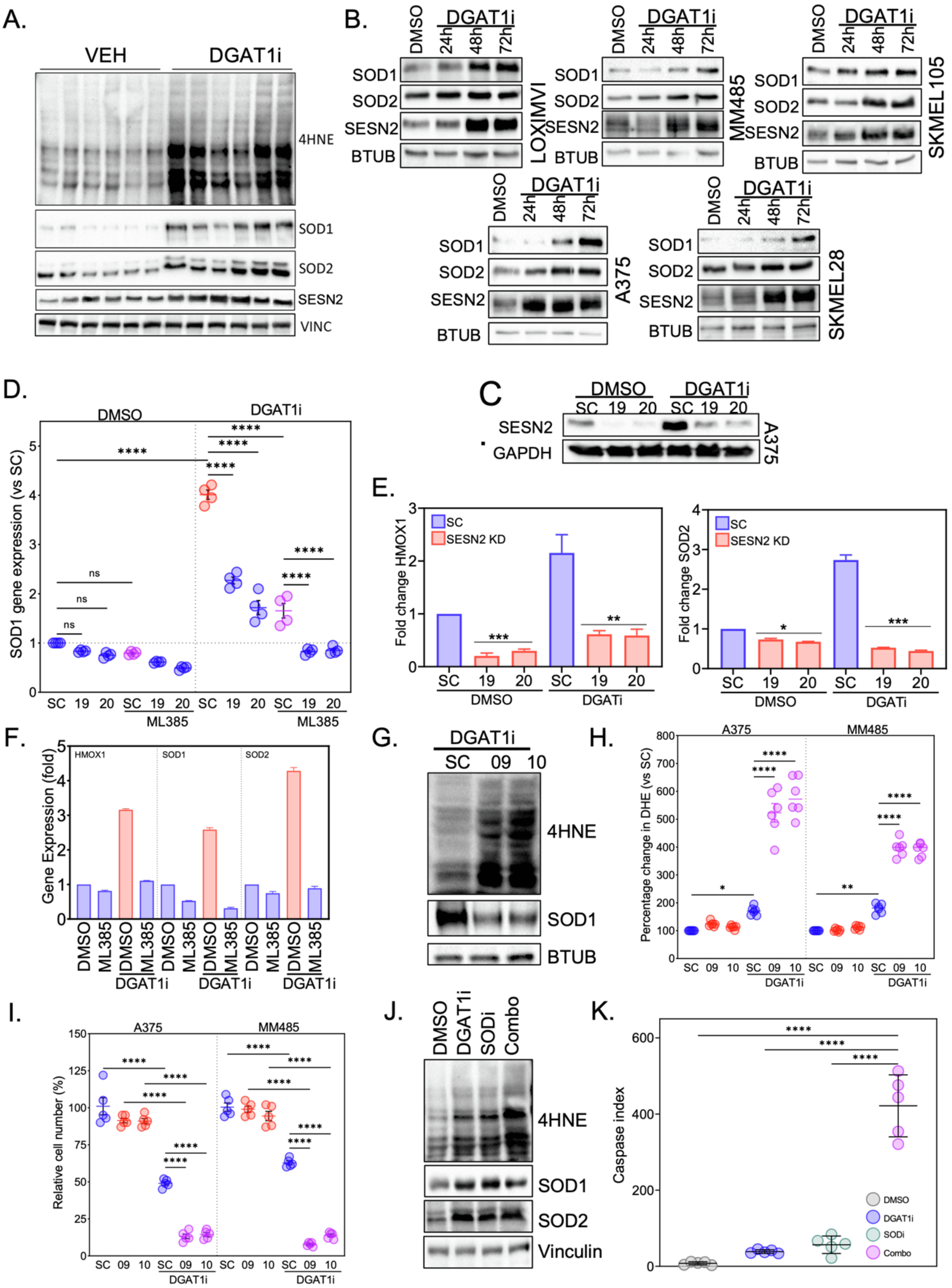

A.
VEH
DGAT1i
4HNE
SOD1
SOD2
SESN2
VINC
B.
DMSO
DGAT1i
24h
48h
72h
SOD1
SOD2
SESN2
BTUB
LOXIMVI
MM485
SKMEL105
A375
SKMEL28
C
DMSO
DGAT1i
SC 19 20 SC 19 20
SESN2
GAPDH
A375
D.
DMSO
DGAT1i
SOD1 gene expression (vs SC)
ns
****
SC 19 20 SC 19 20 SC 19 20 SC 19 20
ML385
E.
Fold change HMOX1
Fold change SOD2
SC
SESN2 KD
***
**
*
DMSO
DGATi
F.
Gene Expression (fold)
HMOX1
SOD1
SOD2
DMSO
ML385
DGAT1i
G.
DGAT1i
SC 09 10
4HNE
SOD1
BTUB
H.
A375
MM485
Percentage change in DHE (vs SC)
SC 09 10 SC 09 10
DGAT1i
I.
A375
MM485
Relative cell number (%)
SC 09 10 SC 09 10
DGAT1i
J.
DMSO
DGAT1i
SODi
Combo
4HNE
SOD1
SOD2
Vinculin
K.
Caspase index
DMSO
DGAT1i
SODi
Combo

Figure S6 Combined DGAT1 and SOD1 inhibition halts tumor growth (related to Figure 6)

(A) 4-Hydroxynonenal (4HNE) protein conjugate abundance and protein expression of SOD1, SOD2 and SESN2 in A375 tumors treated with either vehicle or A922500 (DGAT1i).

(B) Protein expression of SOD1, SOD2 and SESN2 following A922500 treatment for 24-72 hours in a panel of melanoma cell lines.

(C) Protein expression of SESN2 in A375 cells following treatment with/without A922500 and with either scrambled control (SC) or SESN2 targeting siRNA (19 or 20) for 48 hours.

(D) SOD1 gene expression in A375 cells following treatment with/without A922500 and ML385 and treatment with either scrambled control (SC) or SESN2 targeting siRNA (19 or 20) for 48 hours. (Mean ± SD, n=3)

(E) HMOX (left) & SOD2 (right) gene expression in A375 cells following treatment with/without A922500 and with either scrambled control (SC) or SESN2 targeting siRNA (19 or 20) for 48 hours. (Mean ± SD, n=3)

(F) HMOX, SOD1 & SOD2 gene expression in A375 cells following treatment with/without A922500 and with/without ML385 for 48 hours. (Mean ± SD, n=3)

(G) 4HNE protein conjugate abundance and protein expression of SESN2 in A375 cells following treatment with A922500 and with either scrambled control (SC) or SOD1 targeting siRNA (09 or 10) for 48 hours.

(H) Quantification of ROS levels using dihydroethidium (DHE) fluorescence in A375 cells following treatment with/without A922500 and with either scrambled control (SC) or SOD1 targeting siRNA (09 or 10) for 48 hours. (Mean ± SD, n=6)

(I) Relative cell number following crystal violet staining in A375 cells following treatment with/without A922500 and with either scrambled control (SC) or SOD1 targeting siRNA (09 or 10) for 48 hours. (Mean ± SD, n=5)

(J) 4HNE protein conjugate abundance and protein expression of SOD1 and SOD2 in A375 cells following treatment with either A922500 or ATN-224 (SODi) alone or in combination.

(K) Cleaved-caspase index in A375 cells following treatment with either A922500 or ATN-224 alone or in combination (combo). Fold-change relative to DMSO (Mean ± SD, n=5). For significance: $*p < 0.05$, $**p < 0.01$, and $***p < 0.001$

Cell line	Genetic drivers	DGAT1 status
888MEL	$BRAF^{V600E}$	DGAT1 low
SKMEL28	$BRAF^{V600E}$	DGAT1 medium
SKMEL2	$NRAS^{Q61R}$	DGAT1 over expressed
MM485	$NRAS^{Q61R}$	DGAT1 over expressed
A375	$BRAF^{V600E}$	DGAT1 over expressed
SKMEL105	$BRAF^{V600E}$	DGAT1 over expressed
LOXIMVI	$BRAF^{V600E}$	DGAT1 amplified
SKMEL5	$BRAF^{V600E}$	DGAT1 amplified

Supplementary Table 8 DGAT1 status of cell lines used in this study (related to STAR Methods)
